# In situ diffraction monitoring of nanocrystals structure evolving during catalytic reaction at their surface

**DOI:** 10.1038/s41598-023-28557-5

**Published:** 2023-01-26

**Authors:** Maciej Zieliński, Zbigniew Kaszkur, Wojciech Juszczyk, Janusz Sobczak

**Affiliations:** 1 NOMATEN Centre for Excellence - National Centre for Nuclear Research, A. Soltana 7, 05-400 Otwock-Swierk, Poland; 2 Institute of Physical Chemistry, Polish Academy of Sciences, M. Kasprzaka 44/52, 01-224 Warszawa, Poland

**Keywords:** Catalytic mechanisms, Characterization and analytical techniques

## Abstract

With decreasing size of crystals the number of their surface atoms becomes comparable to the number of bulk atoms and their powder diffraction pattern becomes sensitive to a changing surface structure. On the example of nanocrystalline gold supported on also nanocrystalline ${\text{CeO}}_2$ we show evolution of (a) the background pattern due to chemisorption phenomena, (b) peak positions due to adsorption on nonstoichiometric ${\text{CeO}}_{2-x}$ particles, (c) Au peaks intensity. The results of the measurements, complemented with mass spectrometry gas analysis, point to (1) a multiply twinned structure of gold, (2) high mobility of Au atoms enabling transport phenomena of Au atoms to the surface of ceria while varying the amount of Au in the crystalline form, and (3) reversible ${\text{CeO}}_2$ peaks position shifts on exposure to He–X–He where X is O_2_, H_2_, CO or CO oxidation reaction mixture, suggesting solely internal alternations of the ${\text{CeO}}_2$ crystal structure. We found no evidence of ceria lattice oxygen being consumed/supplied at any stage of the process. The work shows possibility of structurally interpreting different contributions to the multi-phase powder diffraction pattern during a complex physico-chemical process, including effects of physi-, chemisorption and surface evolution. It shows a way to structurally interpret heterogeneous catalytic reactions even if no bulk phase transition is involved.

## Introduction

During the last decades activity of nanocrystalline gold in CO oxidation^1,2^ has been covered by numerous publications in attempt to explain its origin^3,4^. The quest for understanding involved evolution of views on the role played in the catalytic reaction by high gold dispersion^5^. Also formation of unstable gold oxides was suggested as a key factor in CO oxidation^6,7^. Presence of water in the environment may have considerable effect on the reaction rates^8^. It appears that Au may significantly affect electronic properties of ceria^9,10^, or form active complexes with ${\text{CeO}}_2$^11^ that may result in surface reconstruction^12,13^ or bulk structural rearrangement in reaction conditions. For the low temperature reaction the question of involvement of the ceria lattice oxygen is a subject of a long standing and not resolved debate^14,15^ in spite of the evidence provided by the isotope exchange experiments^16–18^, showing no lattice oxygen exchange at temperatures lower than 570K.

All measurements of ceria lattice swelling in catalytic reactions are understood as rising population of oxygen vacancies apparently supporting conclusions on the lattice oxygen exchange and Mars-van Krevelen reaction mechanism. However it is well known that the real cause of the lattice swelling is rising population of ${\text{Ce}}^{3+}$ ions occupying larger volume in the lattice. In some situations it is possible to increase this population not affecting number of oxygen vacancies. It is e.g. considered in DFT description of CO adsorption on ceria when formation of surface COO species leads to charge transfer and a net reduction of the surface with electron localized on Ce, creating ${\text{Ce}}^{3+}$ surface or subsurface ions^19^. This process can be detected by XRD peak shift or Raman spectroscopy $460 \, {\text{cm}}^{-1}$ peak shift^20^ but does not create oxygen vacancy if no ${\text{CO}}_2$ desorption occurs. If the ceria is subsequently flushed with He, the new equilibrium will be achieved and collisions with atoms from high energy tail of Maxwell distribution may quickly (in the real time) dissociate COO with desorption of CO and restore the original state of the surface. A similar phenomenon has been observed by us when adsorbing hydrogen on platinum^21^. Such scenario agrees with results of the techniques of isotope exchange, directly addressing this problem. They clearly show no oxygen exchange in CeO_2_ below 350 °C^16–18^. Gold supported catalysts can slightly promote the exchange (by 40%) but the promotion is insignificant if the exchange does not run at all^16^. Considering rather small turnover frequency achieved during the CO oxidation reaction, at many surface sites CO adsorption occurs without ${\text{CO}}_2$ evolution and results in the net ceria lattice swelling.

To our best knowledge all used in the literature experimental arguments supposed to proof the lattice oxygen exchange during low temperature CO oxidation on ceria based catalyst, can be rationalized assuming no oxygen exchange in agreement with the isotope exchange experiments^16–18^. Especially Raman peak shift information comes from the catalyst surface and is more sensitive to appearance of the induced ${\text{Ce}}^{3+}$ ions. In our opinion the Mars-van Krevelen mechanism of these low temperature reactions is a multiply repeated myth and our results provide arguments supporting our claim. Slightly different mechanism may play role in hydrogen adsorption on ceria. It adsorbs dissociatively on Au and spills over ceria. The bonding with the surface has to involve electron transfer increasing surface concentration of ${\text{Ce}}^{3+}$ ions. This should cause the lattice swelling. A number of experimental studies^22–24^ report data consistent with uptake of hydrogen into the ceria lattice and formation of hydroxy group. Theoretical DFT calculations predict then appearance of a characteristic IR band close to the experimentally observed one at $3510 \, {\text{cm}}^{-1}$ confirming the hydrogen uptake^25^. In this case one lattice oxygen takes over one electron leaving one electron on the neighbor Ce that is transformed into ${\text{Ce}}^{3+}$. This process again increases ceria lattice parameter. The lattice expansion predicted theoretically^25^ − 1.5% - compares well with the values observed experimentally at different temperatures − 0.6 to 2.1%^24^. Again, similarly like for CO adsorption, flushing the sample with He shifts the equilibrium leading to quick hydrogen desorption, association on Au and evolution restoring the original lattice parameter.

The gold-support interface appears to play important role, in literature attributed to strain related to gold epitaxy on oxide surface^26^ or to charge distribution. It is growing consent that the activity is mostly shaped by the gold-support perimeter^27^ or small Au islands on ceria. Connecting dispersed Au islands with the reaction active sites is not a new idea. The experiments with leaching of Au, leave at the ceria surface only the strongest bound Au with no change in activity^28^. Our experiment suggests extension of the perimeter to even wider area. Theoretical investigation of a possible reaction path for small Au clusters by Kim^11^ proposes several reaction mechanisms where the active sites are ${\text{Au}}-{\text{Ce}}^{3+}$ complexes available in the neighborhood of oxygen vacancies. The proposed direct mechanism involves ${\text{O}}_2$ adsorption at the ${\text{Au}}-{\text{Ce}}^{3+}$ bridge site, CO adsorption at Au and conversion to ${\text{CO}}_2$ with low energy barrier (0.08 eV) with Au−O* left for another cycle of CO oxidation. No lattice oxygen is involved so this mechanism is considered in opposition to Mars-van Krevelen one. ${\text{CO}}_2$ desorption involves surpassing another energy barrier of 0.22 eV but still can proceed spontaneously at relatively low temperature^11^. This mechanism involves ${\text{O}}_2$ and CO not competing for adsorption site.

Considering structural changes a fundamental question about the initial Au nanoparticles morphology has to be asked first. It is usually answered by TEM or ETEM study^29–31^ but also attempted by AFM/STM^32,33^ or diffraction using Debye function analysis^34^ or PDF method^35^. During the catalytic reaction, formation of some transient non-stable phases can be also expected, and they can be noted and accounted for only with an experimental in situ technique. In literature, the activity was attributed to the catalyst storage capacity of active oxygen which was considered in terms of storage capacity of the support as well as of gold alone e.g. by formation of unstable gold oxides.

To get structural insight into the running reaction we attempted to reveal the full power of in situ powder diffraction by very careful and detailed analysis of the pattern evolution. The conclusions were drawn on the basis of differences between the patterns when composition of the gas atmosphere was the only varying parameter. We have shown by monitoring a long series of measurements with good counting statistics, that the pattern repeatability surpass markedly the level of systematic diffractometer errors. As those errors are well repeatable in a series of measurements, the differences between the patterns can be analyzed in fine detail, limited mostly by the counting statistics. Such an analysis had already allowed e.g. in situ observation of a subtle surface reconstruction of Pt nanoparticles^36^ never seen before by diffraction. The proposed tools (unlike e.g. Rietveld refinement) are able to detect small peak shifts and intensity evolution correlated with the number and arrangement of the scattering atoms building the average nanoparticle. In this way powder diffraction offers deep insight into structural changes at atomic level and into the reaction mechanism, not exploited before. We show that the gold morphology is disordered quasicrystalline and is not significantly altered during the reaction but the Au atoms are highly mobile and, depending on chemical environment, migrate to ceria support and back, what prompts the suggested reaction mechanism. The observed subtle changes of ceria lattice parameter (LP) are suggested to result from the lattice oxygen diffusion to or from the nanoparticle’s surface or from formation of subsurface ${\text{Ce}}^{3+}$ species with no change of the overall stoichiometry^37^. The mobile oxygen does not seem, however, to be active. The experimental data seem to connect catalyst activity in CO oxidation to Au anchored next to the oxygen vacancy and the lattice oxygen is unlikely to participate in the reaction. The data support well the mechanism of a direct CO oxidation reaction proposed by Kim^11^ that differ from the Mars-van Krevelen scheme, usually suggested for Au similarly like for Pt/${\text{CeO}}_2$^38^. The observed Au dynamics can be supported by further DFT calculations evaluating stability of Au clusters bound to ${\text{Ce}}^{3+}$ during adsorption of gases. It is known to be sensitive to the environment^39,40^.

## Results

**Figure 1:**
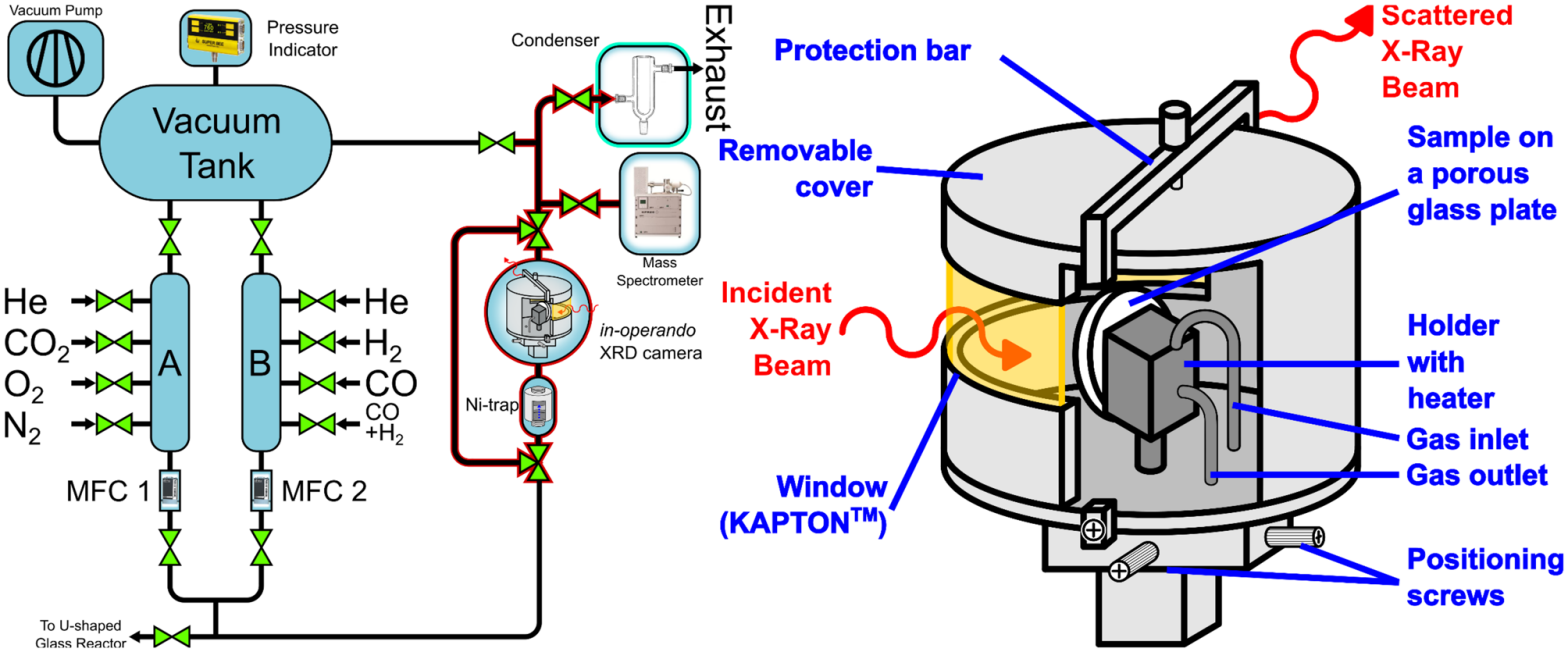
Scheme of experimental setup and of PXRD environmental chamber. For the detailed description refer to Electronic Supplementary Information.

XRD monitoring of catalytic reactions requires data collection during stationary state of the reaction when the pattern should be strictly repeatable. In our case, the XRD measurement with good counting statistics is the most time consuming stage, the monitoring takes usually a long time, even using efficient detectors. The setup used in the reported experiments is presented in Fig. 1. The setup allows to control fed gas flow, purity/contents, and sample temperature.

The most critical issues in in situ diffraction monitoring of catalytic reactions are summarized in the “Methods” section. They include sample selection and diffraction pattern (DP) repeatability, gas purity, camera tightness, absorption correction and temperature monitoring.

### Samples and data collection

The study has been performed on 9.4 wt% ${\text{Au/CeO}}_2$ catalyst. Its preparation is described in Electronic Supplementary Information (ESI). For the sake of comparison we synthesized and analyzed also catalysts of 20 wt% Au/C and 7.2 wt% ${\text{Au/SiO}}_2$ . Their synthesis and characterization is described in ESI and occasional reference to these data is used to fortify conclusions appearing from analysis of the ${\text{Au/CeO}}_2$ sample.

Sample preparation started from its milling in the mortar to obtain fine-ground material and to facilitate sample deposition on the sintered porous glass plate. Additionally, in the PXRD experiments, an aliquot of polycrystalline quartz was mixed with the sample. It was used as an internal reference material to calculate corrections for systematic errors (especially specimen off-axis displacement) and as an internal probe of the temperature of the catalyst (details on temperature dependence of quartz structure are described in ESI). The off-axis shift was determined independently for each pattern and used as correction for LP estimation.

The gas sequence was designed so that it enabled investigation of physical and/or chemical adsorption of gases on the catalyst’s surface as well as phenomena accompanying the stoichiometric CO oxidation (abbrev.: sCOOX) or its preferential oxidation in the ${\text{H}}_2$-rich stream (abbrev. PROX). For PROX, an nonstoichiometric ratio of CO and ${\text{O}}_2$ was used (2-fold excess of oxygen, CO : ${\text{O}}_2$ = 1 : 1 by volume) to check readily the catalysts’ selectivity. The sequence was as follows: He1, ${\text{H}}_2$, He2, CO, He3, ${\text{O}}_2$, He4, sCOOX, He5, PROX, sCOOX, PROX, He6, ${\text{CO}}_2$, He7 (marking of a subsequent He exposure as He# is applied in figures). The measurements were run at heating block temperature of 160 °C. The Au/CeO_2_ catalyst was found to reach nearly 100% conversion of CO to ${\text{CO}}_2$ already slightly above 80 °C. If any water is formed in competition with CO oxidation when hydrogen was added to the gas mixture, the setup was required to stay at least at 100 °C to avoid ${\text{H}}_{2} {\text{O}}$ condensation. Finally, the experiment set temperature of 160 °C hindered formation of ${\text{Ni(CO)}}_4$^41^. The estimated temperature of the catalyst layer in He was 150 °C.

As shown in the “Methods” section, the use of not diluted stoichiometric CO oxidation mixture (sCOOx) together with measurement of the catalyst grain temperature via internal standard, allowed estimation of a turnover frequency per catalyst grain. However the temperature increase does not seem to affect detectable effects of the reaction mechanism.

### Catalyst activity and temperature

Activity of the 9.4 wt% Au/${\text{CeO}}_2$ catalyst was tested twice: in a standard U-shaped glass reactor with fixed catalyst bed, and in the XRD camera during DP acquisition following the designed gas exposure sequence. Catalytic activity was monitored each time only qualitatively by MS. The obtained MS calibration occurred to be unstable and not fully reliable during a-few-day in-operando experiment, so, consequently, we resigned from detailed interpretation of the chemical output of reaction. The general conclusions were drawn by comparison of MS signals on continuous MS spectra.

**Figure 2:**
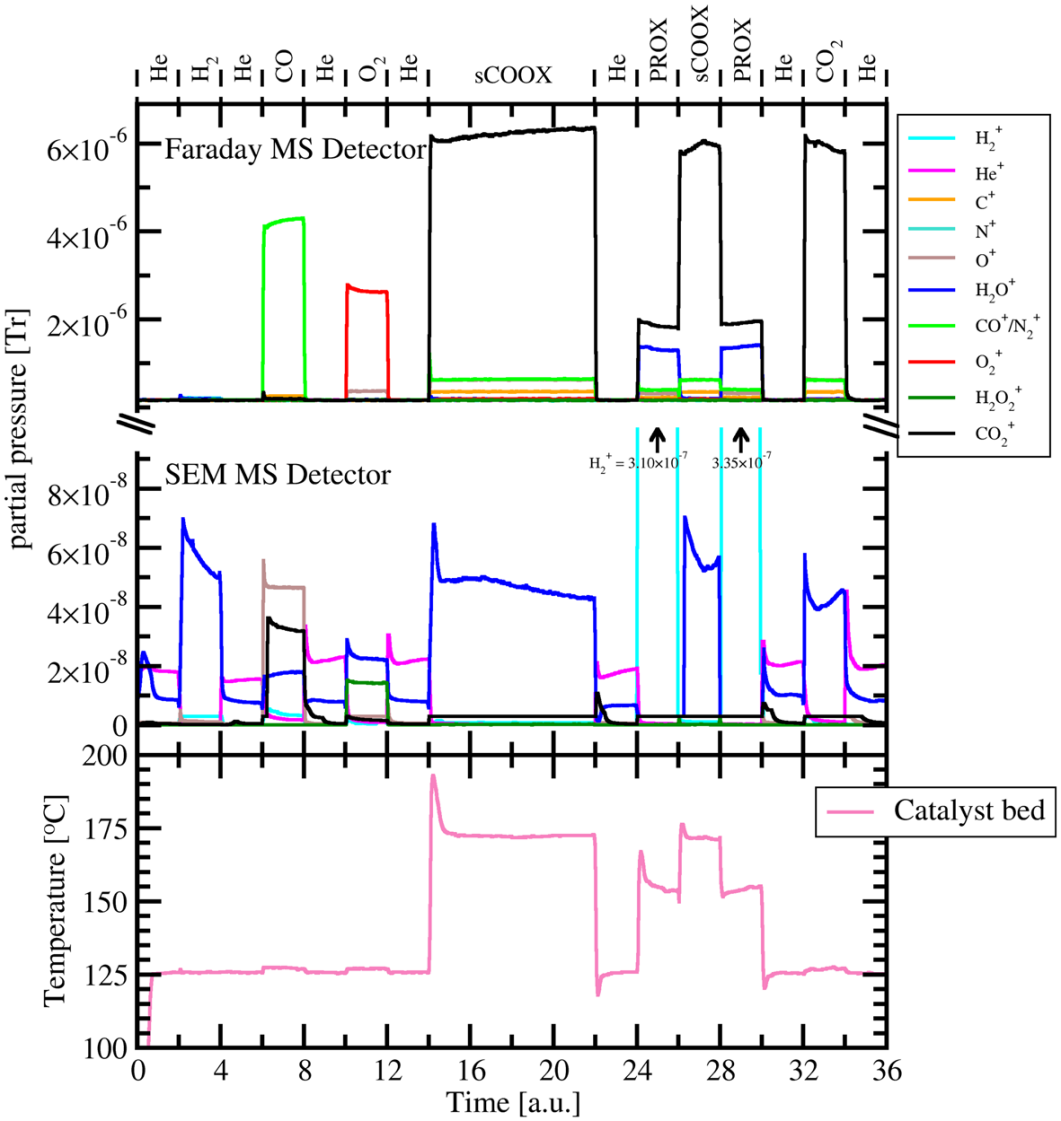
MS probed outlet gas composition and thermocouple measured catalyst bed temperature for the sequential experiment with Au/${\text{CeO}}_2$ catalyst in the glass reactor. The time scale corresponds to 30 min per unit.

**Figure 3:**
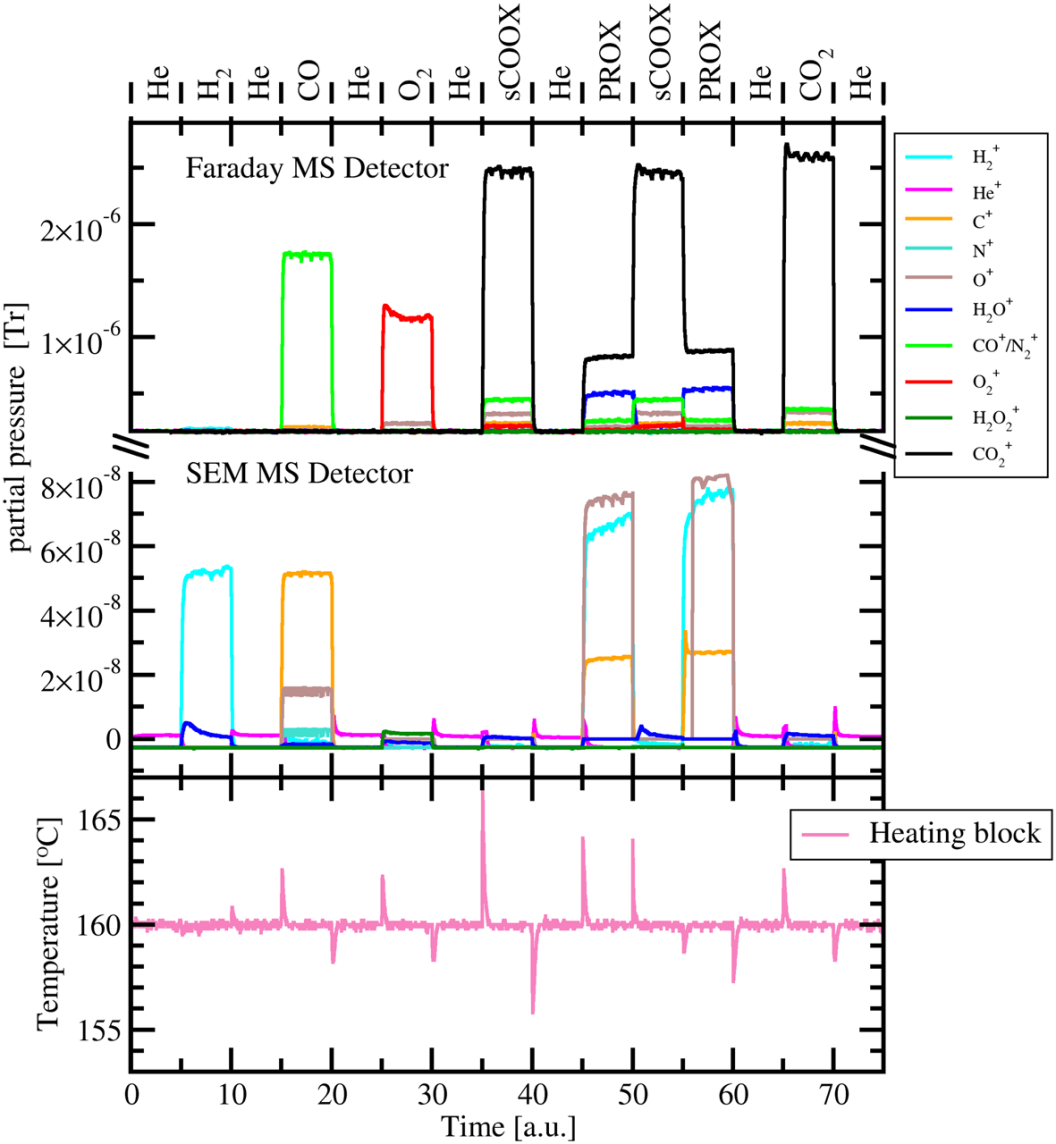
MS probed outlet gas composition and thermocouple measured heating block temperature for the sequential experiment with Au/${\text{CeO}}_2$ catalyst in the PXRD chamber. The time scale corresponds to DP number, with 5 DPs per each gas exposure. One DP measurement lasted about 60 min.

Under studied conditions, pure ${\text{CeO}}_2$ proved to be rather inactive in CO oxidation. On the contrary, Au/${\text{CeO}}_2$ catalyst reached close-to-full conversion of oxygen to carbon dioxide in both sCOOX and PROX reactions. For the glass reactor we used 212 mg of 9.4 wt% Au/${\text{CeO}}_2$ and the temperature was set to 125 °C. For the XRD camera we used 50 mg of 9.4 wt% Au/${\text{CeO}}_2$ catalyst and the temperature set to 160 °C. The catalytic activity in stoichiometric and selective (preferential) CO oxidation was studied under programmed sequence of gases. The chemical performance results are presented in Fig. 2 for the glass reactor, in Fig. 3 for the XRD camera and in Fig. S3 (in ESI) to be compared to Figs. S39 and S40 of ESI presenting data for blank experiments with no sample.

### Diffraction pattern analysis

**Figure 4:**
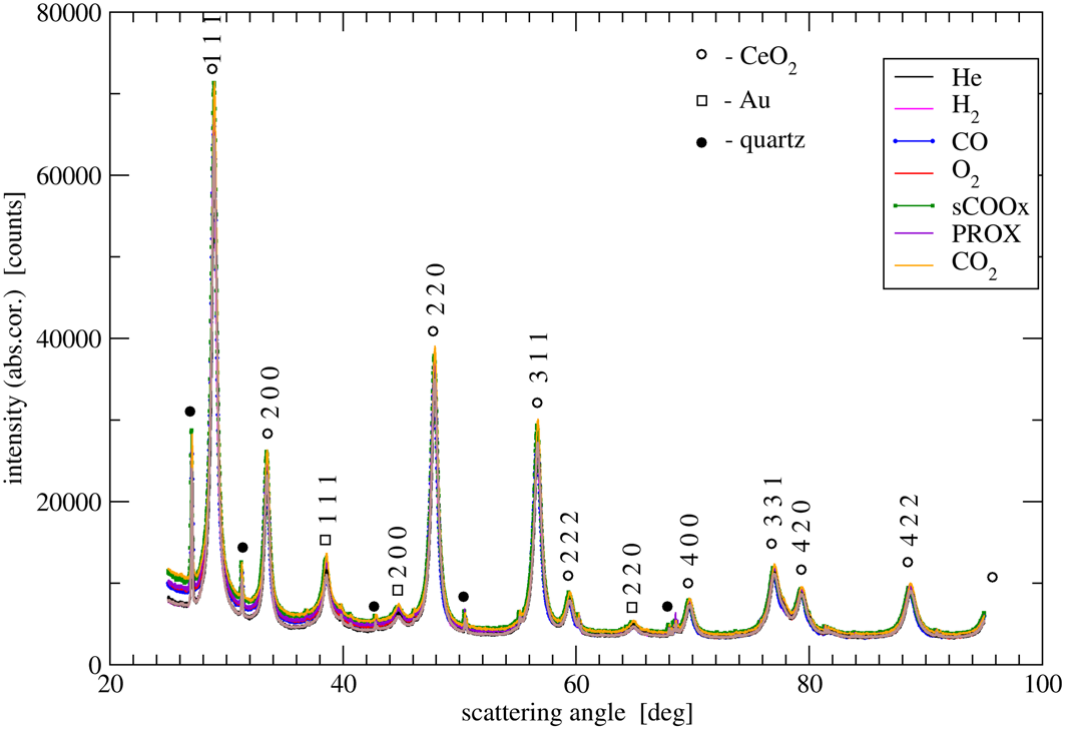
DPs of the ${\text{Au/CeO}}_{2}$ catalyst exposed to a sequence of gases.

DPs, corrected for absorption of X-rays by the gas, collected during the experiment and averaged over the last 4 (out of 5) patterns measured during each gas exposure, are presented in Fig. 4.

The powder DPs were analyzed following steps discussed in the next section. They included correction for gas absorption of X-rays in the chamber (camera), background analysis and subtraction and fitting (Fityk 1.3.0^42,43^) of peaks of ceria phase (FCC, JCPDS-ICDD 81-0792) to doublets of Voigt profiles corresponding to $K_{\alpha _1}$ and $K_{\alpha _2}$ components of the $K_{\alpha }$ spectral line of X-ray tube Cu anode. The peaks analysis involved estimation of crystal size and microstrain following Williamson-Hall approach^44,45^ and LP estimation from Nelson-Riley extrapolation scheme^46^.

**Figure 5:**
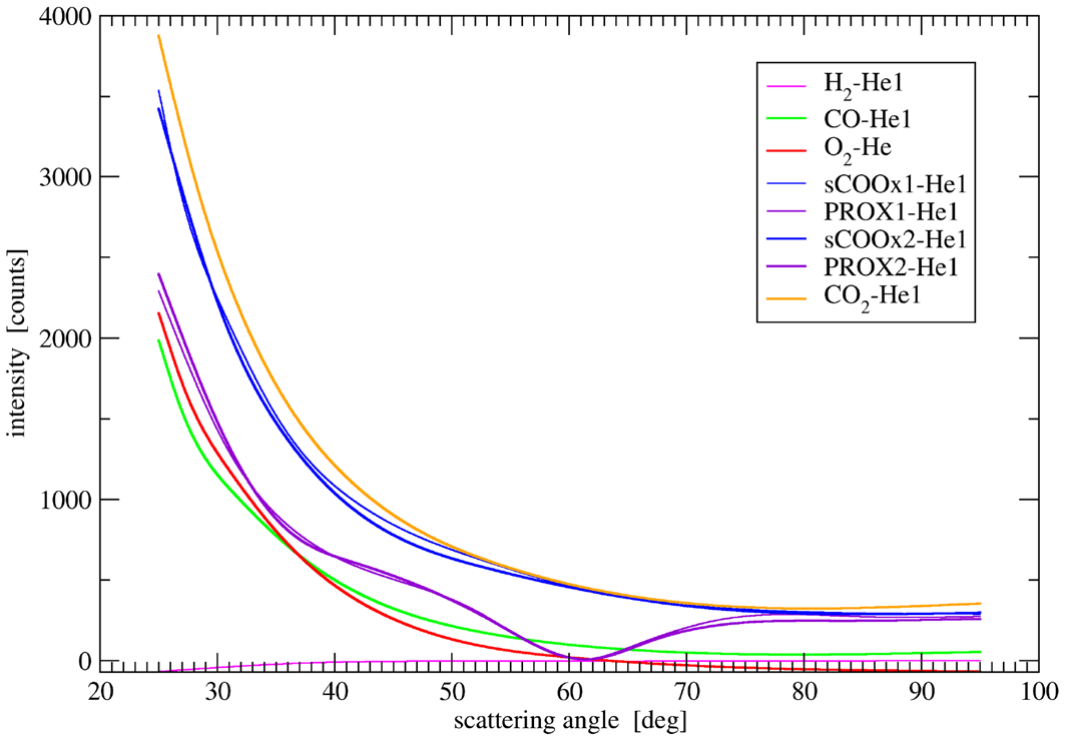
Background difference of the catalyst DPs measured at several gases and of the initial pattern in He. All patterns in He overlap with good accuracy.

The background subtraction required careful consideration of (a) the contributions of the specimen holder (sintered glass plate), (b) sample amorphous component and, (c) the scattering from the adsorption layer on the sample surface as well as from the content of the pores in the sample. The background subtraction could be performed precisely starting from estimation of the background profile for the sample in He (the He1 experiment stage is considered) by fitting its polycrystalline peaks to pairs (reflecting Cu $K_{\alpha _1}$ and $K_{\alpha _2}$ lines) of Voigt profiles following rules described before^47^. The residual scattering profile resulting from the best fit was accepted as a background for He. Due to small scattering power of He atoms, this background can be assumed to cover (a) and (b) contributions. Hence, this profile has been considered to be the common background contribution for all other DPs collected in a non-inert environment. The scattering contribution from the non-noble gas molecules has been assessed through evaluation from the gain in the background level. After subtracting the He1 DP from the DPs collected under a particular gas, the differential curve is approximated by a smooth sp-line function. These sp-line functions, covering the component (c) (presented in Fig. 5), added to the common background contribution (mentioned above) have been regarded as the true total background profiles for each non-inert gas atmosphere. It encompassed all three components of the background and further analyzed was only the background differences between DPs that include the last component, i.e. (c) component. It is interesting to note that the background during sCOOx reaction is much higher than the initial background in He1 and similar to the background in ${\text{CO}}_{2}$ atmosphere suggesting ${\text{CO}}_{2}$ molecules filling the material pores. Also the wavy background during PROX reaction resembles pattern of water with characteristic minimum at 60 deg. corresponding to  4 Å$^{-1}$ (see e.g.^48^, Fig. 9), and indeed water production was detected. Intensity of the sp-line approximated differencial backgrounds is roughly proportional to the scattering factor of the actual adsorbate in line with its interpretation as above, i.e. (c) contribution. After subtraction of the true total background profile, each pattern was decomposed onto doublets of Voigt profiles corresponding to all diffraction peaks. It allowed the analysis mentioned above.

### Lattice parameters evolution

All parameters resulted from linear regression analysis of the ceria peak positions and widths. The listed error corresponds to statistical regression error. These parameters are listed for the catalyst (Table 2) and for the ceria support alone (Table 1).

**Table 1:** Lattice parameters for ${\text{CeO}}_2$ blank experiment (Nelson–Riley extrapolation from 6 peaks).

The time scale gas atmosphere	Cryst. size (nm)	Microstrain	Lattice par. (Å)
He1	14 (3)	0.004 (1)	5.413 (1)
${\text{H}}_{2}$	15 (3)	0.004 (1)	5.413 (1)
1 He2	15 (3)	0.004 (1)	5.413 (1)
CO	15 (3)	0.004 (1)	5.419 (1)
He3	15 (3)	0.004 (1)	5.413 (1)
${\text{O}}_{2}$	15 (4)	0.004 (1)	5.413 (1)
He4	15 (3)	0.004 (1)	5.413 (1)
sCOOx	15 (2)	0.004 (1)	5.4138 (9)
He5	15 (3)	0.004 (1)	5.4132 (9)
PROX	16 (4)	0.004 (2)	5.414 (1)
sCOOx	15 (3)	0.004 (1)	5.413 (1)
PROX	14 (3)	0.004 (1)	5.4136 (8)
He6	14 (3)	0.004 (1)	5.4129 (9)

The last figure regression error given in parenthesis.

**Table 2:** Fit parameters of $\text{CeO}_2$ structure for $\text {Au/CeO}_2$ sample from 9 diffraction peaks.

Gas atmosphere	Cryst. size (nm)	Microstrain	Lattice par. a (Å)
He1	16 (3)	0.0044 (8)	5.4161 (4)
${\text{H}}_{2}$	18 (4)	0.0051 (9)	5.4233 (5)
He2	16 (3)	0.0044 (8)	5.4161 (4)
CO	17 (3)	0.0047 (9)	5.4216 (5)
He3	16 (3)	0.0044 (8)	5.4161 (4)
${\text{O}}_{2}$	17 (3)	0.0044 (9)	5.4156 (4)
He4	16 (3)	0.0044 (8)	5.4161 (4)
sCOOx	16(3)	0.0040 (8)	5.416 (1)^a^
He5	17 (4)	0.0043 (9)	5.4147 (4)
PROX	15 (3)	0.0041 (9)	5.417 (1)^a^
sCOOx	16 (4)	0.0040 (9)	5.416 (1)^a^
PROX	16 (3)	0.0040 (9)	5.418 (1)^a^
He6	16 (3)	0.0041 (8)	5.4153 (4)
${\text{CO}}_{2}$	16 (4)	0.0040 (9)	5.4163 (3)
He7	16 (3)	0.0040 (9)	5.4163 (4)

The last figure regression error is given in parenthesis. All values correspond to temperature 150 °C.

^a^The value corrected for the light-off temperature effect.

**Figure 6:**
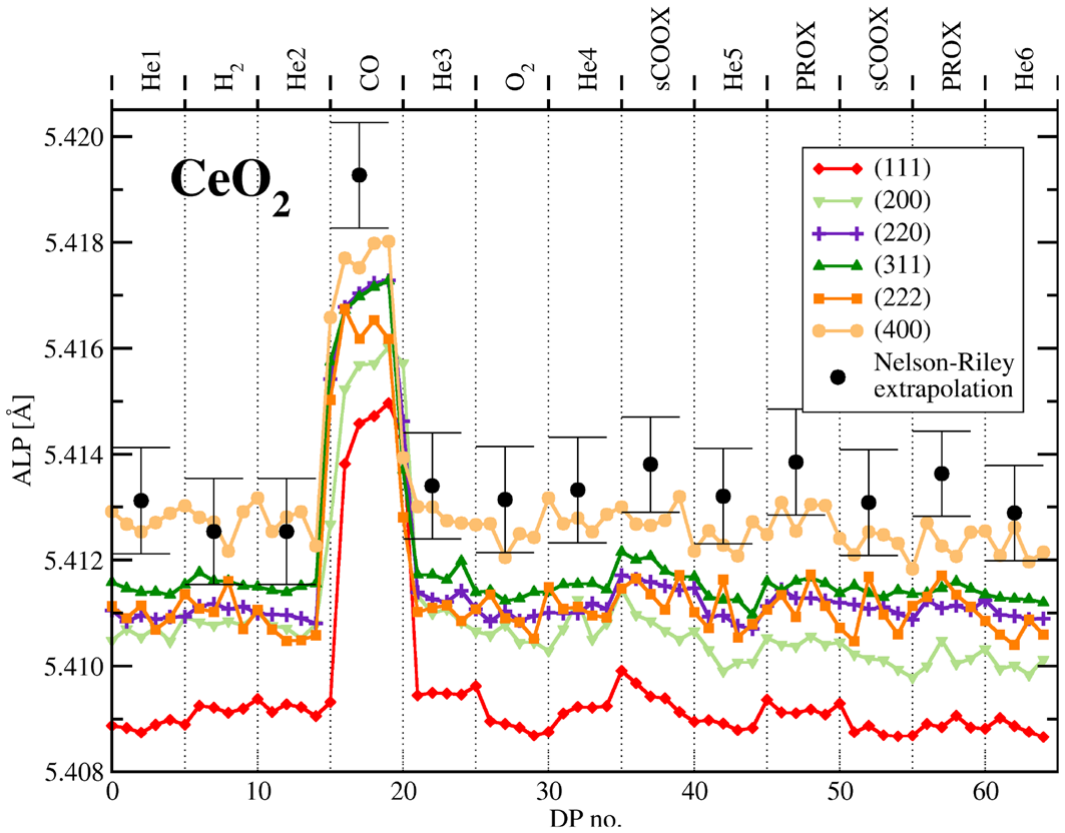
Apparent lattice parameter (ALP) as derived from a number of ${\text{CeO}}_2$ peaks.

**Figure 7:**
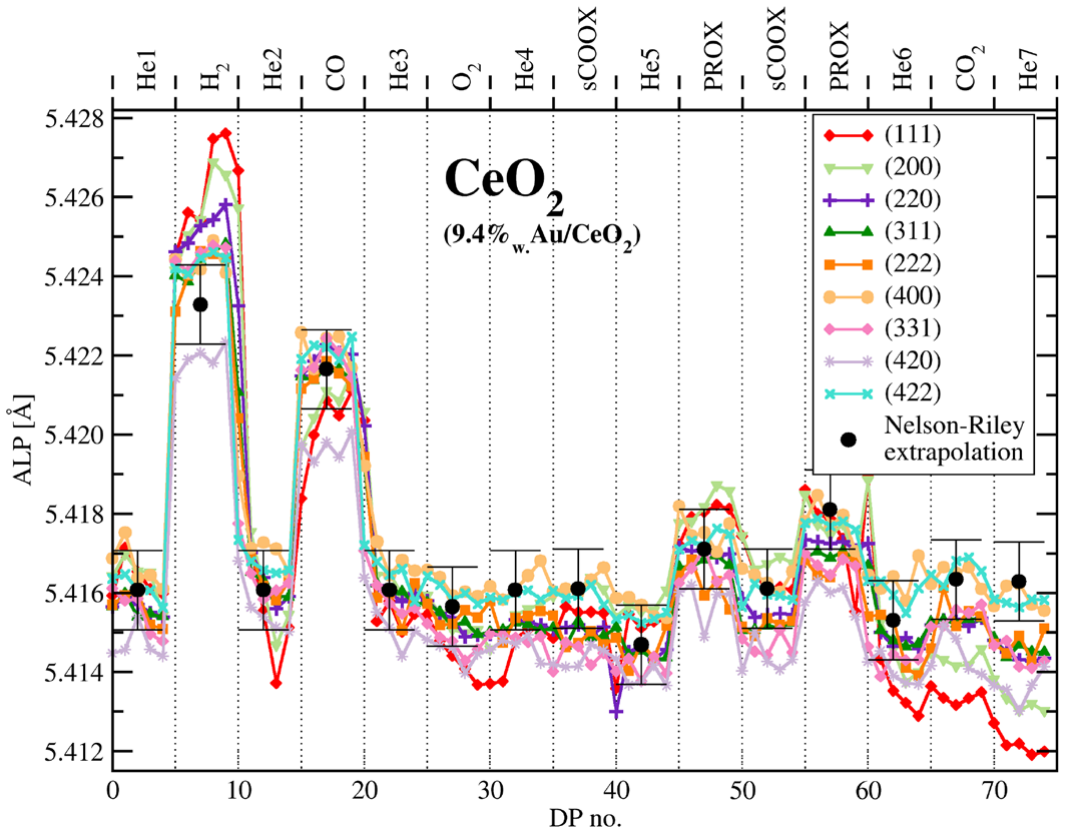
Apparent lattice parameter (ALP) as derived from a number of ceria peaks of the catalyst corrected for the temperature light-off effect.

Differences between the support of the catalyst and the pure ceria mostly concern LPs. The differences in ceria LPs are illustrated in the Figs.  6 and  7.

According to Brauer and Gingerich, and Bishop et al.^49,50^, FCC structure of ${\text{CeO}}_2$^51^ expands when the oxygen content is decreased and ceria becomes a nonstoichiometric oxide, ${\text{CeO}}_{2-x}$. The effect of the changing LP of ceria is usually interpreted in terms of its oxidation state, relating stoichiometry parameter x of ${\text{CeO}}_{2-x}$ to the LP^52^: $a=a_{{\text{CeO}}_{2}} +0.4612*x$. In the case of ceria, this LP change results from ${\text{Ce}}^{3+}$ ion occupying larger volume in the structure (i.e. it has larger ion radius) than ${\text{Ce}}^{4+}$ ion. It has been found that the effective volume of the oxygen vacancy is smaller than that of the oxygen anion^53–57^, but the difference of cerium cation radius is huge enough to compensate even this effect. A change in the observed lattice parameter of ceria is thus mostly indicative of the change of ${\text{Ce}}^{3+}$ population and not directly of oxygen vacancies. Earlier, in 2000, Tsunekawa et al.^58^ has also found that transformation from ${\text{Ce}}^{4+}$ to ${\text{Ce}}^{3+}$ reduces the electrostatic forces inside ${\text{CeO}}_{2-x}$ clusters.

It is clear from Figs.  6 and  7 that gold affects response of ceria to hydrogen whereas ceria response to CO atmosphere remains unchanged. The surface of ceria nanoparticles should respond to adsorption phenomena forming double layer with electron transfer leading to its reduction. This will always result in creation of ${\text{Ce}}^{3+}$ ions or coordinately unsaturated ${\text{Ce}}^{4+}$ sites^59^. For CO adsorption on ceria alone DFT calculations suggest charge transfer and appearance of ${\text{Ce}}^{3+}$ ions even if the temperature is too low for CO oxidation to run^19,60^. This should cause increase of the LP without creating new oxygen vacancies. On desorption the electron transfer restores the former state of the surface and the extra ${\text{Ce}}^{3+}$ ions turn back to ${\text{Ce}}^{4+}$. If CO oxidation is not running, no oxygen vacancy is formed and there is no oxygen exchange in agreement with the isotope exchange experiments^16,17^. For small nanocrystals an additional effect on the diffraction peak position and LP can have the actual distribution of ${\text{Ce}}^{3+}$ species and vacancies. This can be proved considering a FCC particle, in which the surface shell accommodates half of the total atoms number. One can observe a shift of the diffraction peaks positions, hence also the apparent LP (ALP) value, upon altering the inter-atomic distances to be vaguely smaller or larger in the shell than in the core. Indeed, ALP shift is affected mostly by the state of the core part of the particle. It is shown in ESI on the example of a simulated binary FCC alloy when the surface shell of one kind of atoms, accommodates half of the total number of atoms. More general, this phenomenon was theoretically predicted and experimentally observed for PdAg alloy during reversible surface segregation^61^. It is driven by difference in the unit cell size and the diffraction peak position is mostly affected by the unit cells located close to the crystal center.

In hydrogen the simplest and widely accepted explanation of the role of gold is its ability to dissociate dihydrogen that spills over the ceria surface^62,63^. The ALP shift in hydrogen is commonly attributed to ceria reduction but this explanation is in opposition to immediate canceling of the effect after switch to He and to lack of oxygen available for re-oxidation (MS data monitoring oxygen content down to 10 ppm - see comment in section “Method”). We thus suggest alternative explanation via the reversible appearance of ${\text{Ce}}^{3+}$ ions on adsorption, hydrogen uptake^25^ or via changing arrangement of the oxygen vacancies. On exposure to helium the shift of equilibrium leads to desorption, with electron transfer causing oxidation of the newly appeared ${\text{Ce}}^{3+}$ ions back to ${\text{Ce}}^{4+}$ form. Such desorption, although energetically unfavorable is driven by high energy collisions with gas molecules at the far tail of the energy distribution and runs quickly in the real time. All these mechanisms do not involve oxygen exchange that was proven not to run below 570 K by isotope exchange experiments^16,17^. Besides, the LP increase on adsorption alone is expectable, the effect can be small and we observe in Figs.  6 and  7 LP rise by only 0.01 Angstrom corresponding to the apparent change in stoichiometry by 0.022. It is much less than the dispersion of the ceria particles estimated from their size (close to 0.1 depending on the shape model).

The oxygen vacancies can form during ceria synthesis and at high temperature. Theoretical treatment of oxygen-surface vacancy equilibrium^64^ suggests rather high vacancy formation temperatures at experimentally reasonable oxygen pressures. This suggests that pure ceria exposed to gases at temperatures of our experiment, should conserve the overall number of oxygen vacancies. The formation energies for vacancies at the surface and subsurface sites are, however, similar what suggest rather easy vacancy migration in experiment time regardless of relatively high migration barrier (0.87 eV^64^). The energy gain related to chemisorption of CO (or hydrogen) is thus likely to be enhanced by capturing of a subsurface oxygen ion migrating between the vacancy sites. Shift of equilibrium due to decrease of CO (or ${\text{H}}_2$) partial pressure (as result of flushing with He) has then to cause desorption even though the total energy is rising. In line with theoretical considerations above, our results confirm that in our experiments the lattice oxygen is not exchanged with the ${\text{O}}_2$ gas phase. As discussed in section “Method”, the level of oxygen contamination above 10 ppm would be noticed in MS monitoring using SEM detector, but the lower level of oxygen would cause much slower shift of LP than observed and during the exposure to He we would register its gradual evolution.

In our experiments the exposure to ${\text{O}}_2$ does not change LP of ceria as well as of the catalyst. It seems that at temperature of our measurements the superoxide or peroxide species^59^ are either not forming on the reduced surface, or their effect is hardly measurable.

The above results confirm lack of oxygen exchange at temperatures for which many ceria based catalysts are reported to perform well. If no exchange is noticeable on exposure to ${\text{H}}_2$ or CO, there is no point to assume Mars-van Krevelen mechanism of the CO oxidation reaction. This is however suggested in most papers devoted to the subject. The offered support to this claim does not withstand criticism, silently assuming that the measured lattice swelling has to be due to the rising oxygen vacancy population and not to appearing ${\text{Ce}}^{3+}$ ions when the surface is polarized by adsorbate.

### Real temperature, lattice parameter and intensity evolution

The actual specimen temperature in the PXRD chamber was estimated from the peak positions of the quartz dopant using its thermal expansion coefficient that had been determined (see ESI).

**Figure 8:**
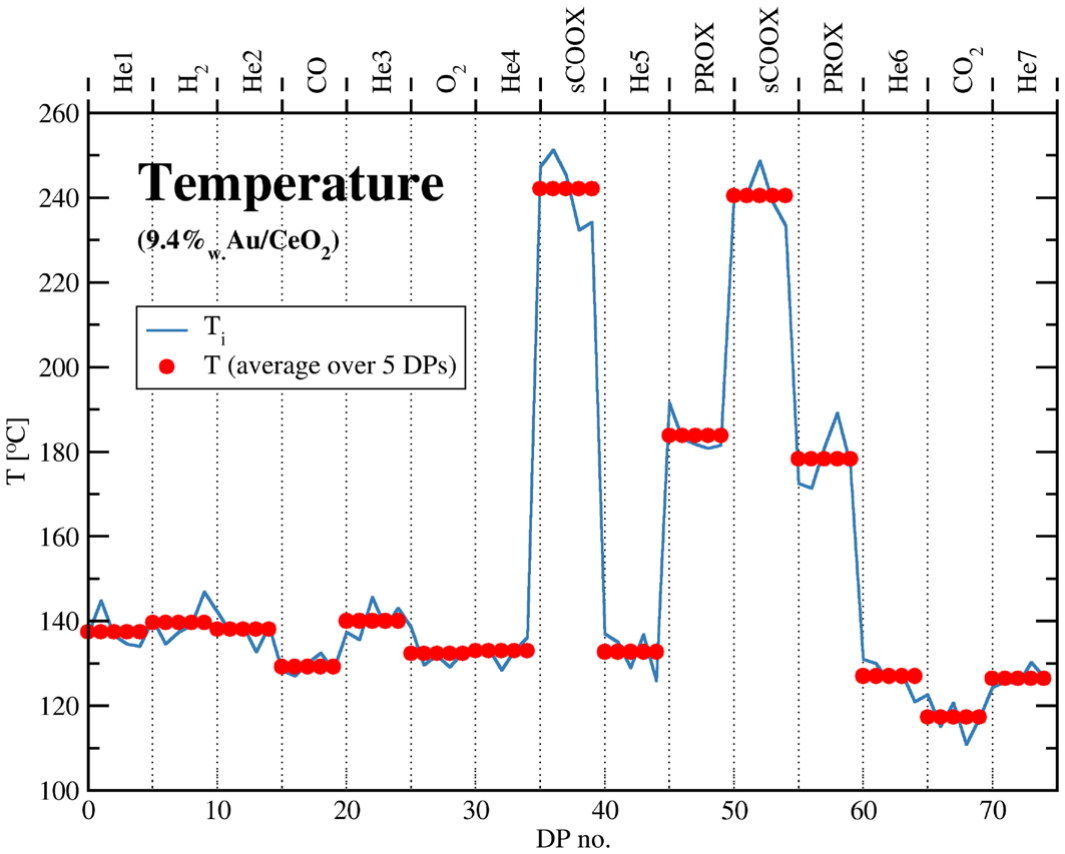
The estimated sample local temperature versus DP number.

**Figure 9:**
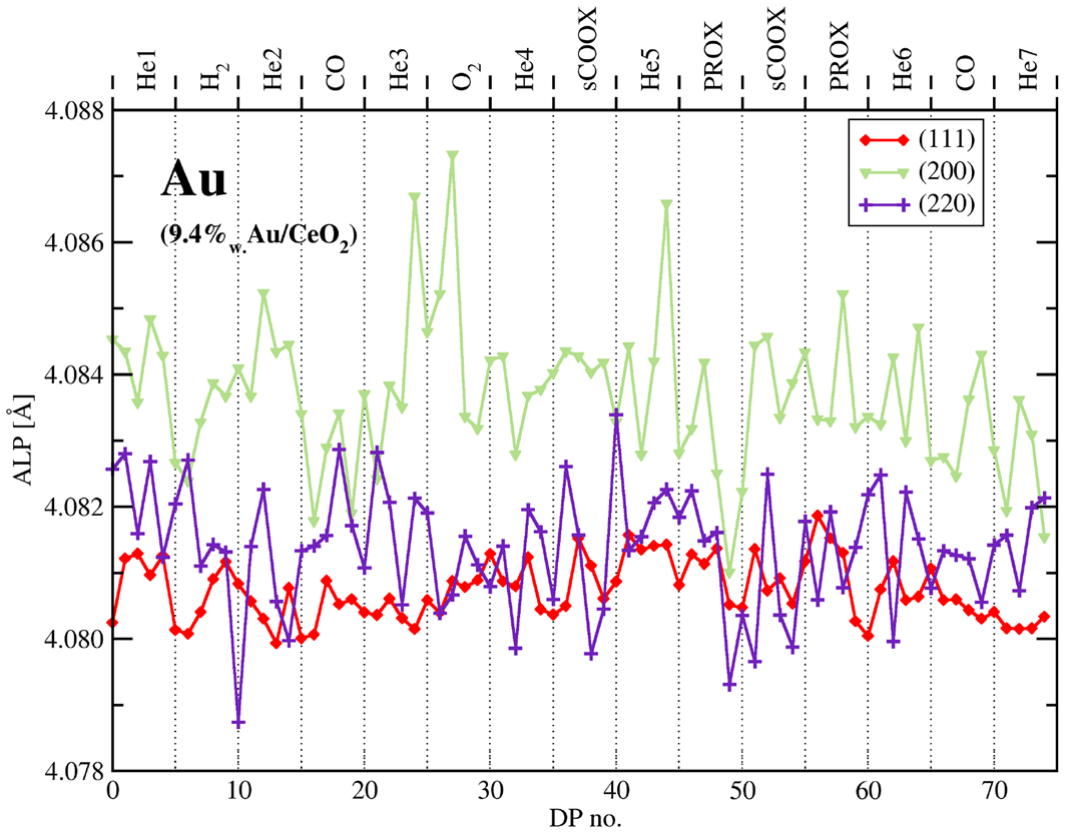
Apparent lattice parameter of gold of ${\text{Au/CeO}}_2$ during the experimental sequence recalculated to the constant temperature.

It is presented in Fig. 8 showing light-off effect during stages of exothermic reaction. The determined values were used to correct LPs in Table  2, Figs. 7 and 9.

The LPs of Au calculated from several peaks visible in a particular DP are different what is characteristic for nanocrystals^65^. It is clear that the apparent lattice parameter (ALP - as calculated directly from the peak position using Bragg’s law) of gold is fairly constant during the experiment. An analysis of a change of gold peak intensity points to changing mobility of gold atoms exposed to different gases. It is clear that peak heights differ together with some changes in the peak width. A hardly noticeable peak narrowing during the experimental sequence is evidently due to slow gold sintering accelerated during steps of exothermic reaction. The peak intensities (sum of 4 measured patterns) were approximated by the profile integral between 35.5 and 42 deg. (111). To estimate the possible change in Debye-Waller factor (DWF) during the gas treatment sequence, the 220 Au peak was also integrated between 63 and 67.5 deg. Table  3 lists integrated 111 intensities as well as a ratio of 220 to 111 intensities assumed to be indicative of DWF changes.

**Table 3:** Au 111 integral peak intensity and a ratio of I(220)/I(111) at different gas atmospheres for ${\text{Au/CeO}}_2$ catalyst.

Gas atmosphere	111 Au integral int.	R = I(220)/I(111) Au
He	12,047	0.232
${\text{H}}_{2}$	12,357	0.230
He	11,991	0.238
CO	12,395	0.252
He	11,765	0.242
${\text{O}}_{2}$	11,988	0.242
He	12,065	0.244
sCOOx	13,023	0.228
He	12,111	0.244
PROX	12,968	0.242
sCOOx	12,977	0.231
PROX	12,881	0.236
He	11,903	0.249
${\text{CO}}_{2}$	12,953	0.251
He	11,815	0.244

Unit of the integral intensity is [counts * deg]. The upper limit of the integral 111 peak intensity error is estimated as 20, the error of the ratio as less than 0.0015.

**Figure 10:**
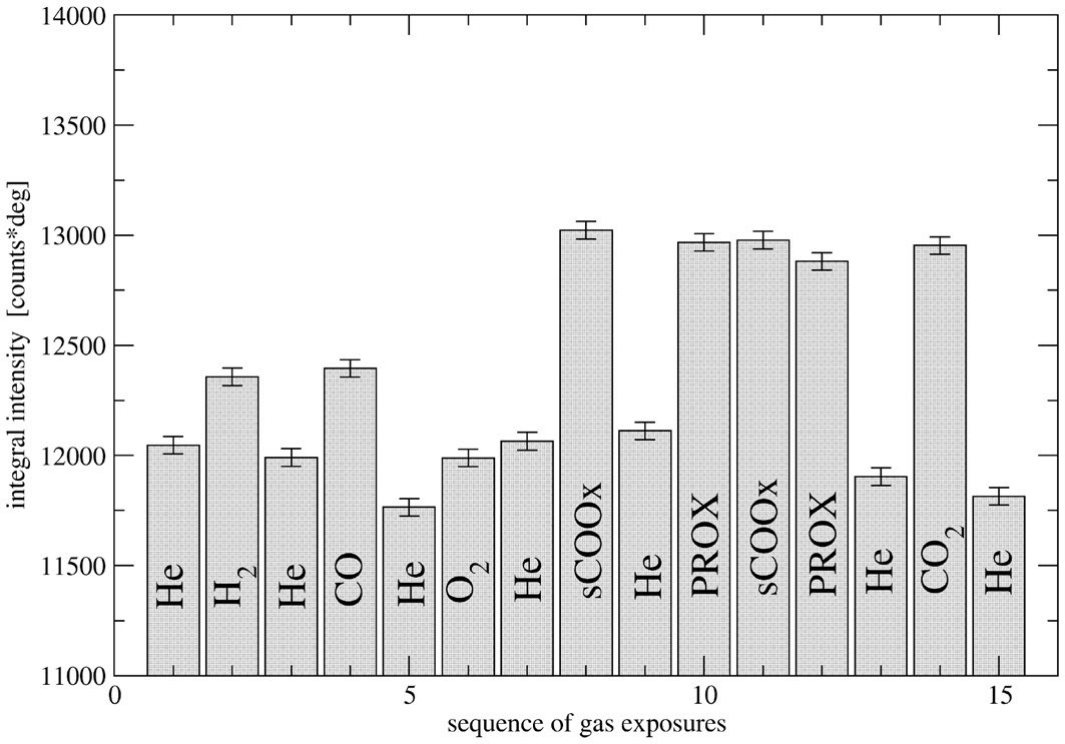
Summary of the Au 111 peak intensity changes during the experimental sequence. Error bars show statistical error.

The integrals include residual contributions from the neighbor ceria peaks (small) as well as peaks of quartz dopant (small of fairly constant intensity). They were deliberately not subtracted to avoid systematic method errors. The upper limit of integration for 111 is arbitrary. The resulting values should, however, reflect changes of the intensity during structure evolution. The peak tails overlap and are nearly flat so the major source of error should origin from statistics of counts. Then the mean squared error of the integral intensity $\sum _{i=1}^{n} I_{i}\Delta$ , where $\Delta$ is the measurement angular step, should be equal to the error of sum of the intensities within the integration limits (above half a million counts with standard deviation of 800) multiplied by $\Delta$= 0.0211 deg. and is less than the assumed overestimate $\sigma ^{2} =I_{aver}\Delta \simeq 20$. The corresponding error of I(220), overestimated for the increased contribution within the integration limits of tails of ceria peaks, equals 13.5 and the error of I(220)/I(111) ratio can then be approximated as less than 0.0015. The Au 111 peak intensity changes exceeding estimated error are thus real. They are displayed in Fig. 10.

In the next section we argue that the Au phase is highly disordered and its surface is mobile. The surface reconstruction and relaxation of Au as a rule changes interplanar distances in the surface layer and affects the ALP^66^ what is not observed. Varying strain in response to various gases is also not likely to occur because it manifests itself in diffraction pattern of multiply twinned crystals (see the next section) by shift of 111 and 200 peaks decreasing their mutual distance. It is not observed as seen from the LP evolution corrected for the temperature on Fig. 9. Then the intensity change cannot be accounted for by surface reconstruction, relaxation or strain and has to be understood in terms of changing DWF and change in the number of atoms contributing to the Au phase. As seen from the Table 3 intensity changes to small degree are related to varying DWF. Maximum change of intensity due to DWF change occurs for CO and ${\text{CO}}_2$. For ${\text{CO}}_2$, out of 7.5% intensity rise, the ordering effect is responsible for the change by 4.8%. Similar degree of ordering can be noted for CO. For PROX, DWF accounts for 2.6% out of 7.6% intensity rise. Minimum DWF effect appears for sCOOX where the intensity rises by 8.1% in spite of 1% disordering. The major factor affecting intensity variation is thus likely a number of Au atoms contributing to the average Au crystal. During the sequence of gas exposures more than 9% of Au atoms are migrating from the Au crystals and back - the number by the order of magnitude exceeding number of Au atoms at the perimeter of average Au crystal epitaxially attached to the ceria support.

### Structural description of gold phase of Au/CeO_2_ catalyst

**Figure 11:**
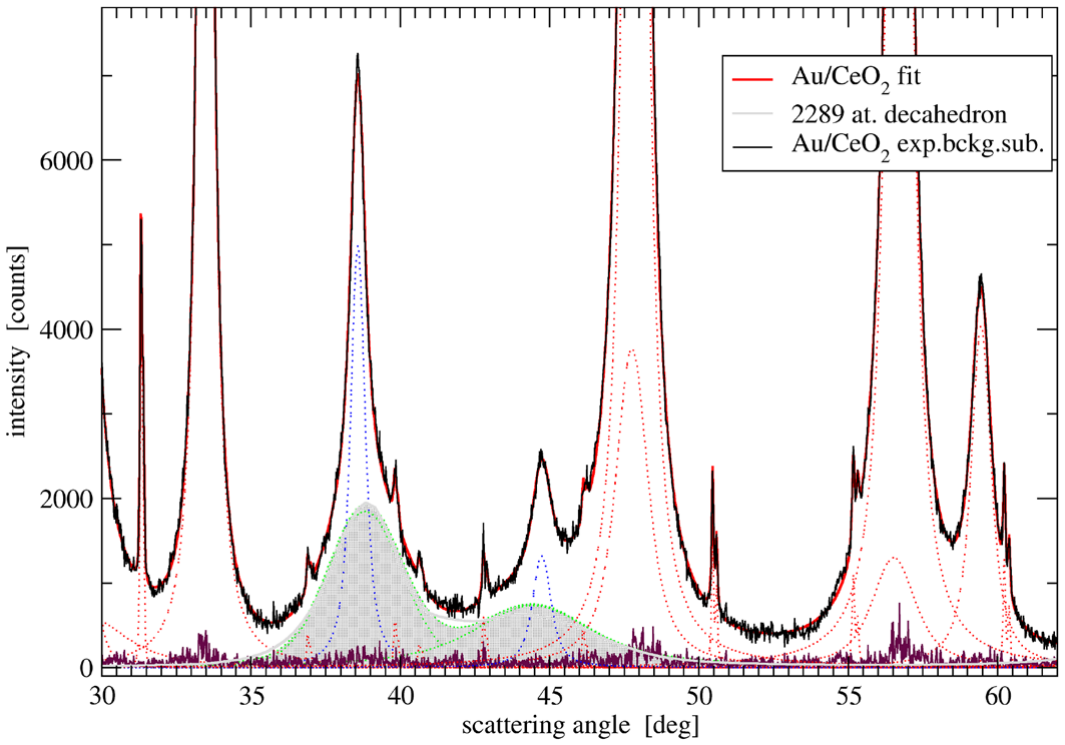
Fragment of DP of ${\text{Au/CeO}}_2$ catalyst (mixed with quartz marker) with ceria peaks fitted by pairs of Voigt function. Au phase could be described by pairs of peaks with broad components (grey) closely resembling calculated pattern of a model decahedron and narrower fcc-like component (dotted blue line). The noisy ‘zero line’ corresponds to error of the overall fit.

The gold 111 and 200 peaks can be fitted also by pairs of profiles but their broad component is shifted in characteristic way—that of 111 to higher, and that of 200 to lower angles as shown in Fig. 11.

This is a typical feature of nanoparticles with high density of stacking faults and twin planes^67^ as well as of pentatwinned uniaxial quasicrystals of which regular or Marks decahedra^68,69^ are the low energy representatives. The characteristic deviations of the peak positions from that expected for fcc structure, rise from relaxation of the strain of twin planes (parallel to [220]). The effect is pronounced for small decahedra and fades for the large ones. The persisting feature is a decreased height and increased broadening of 200 reflection in comparison to the regular FCC DP. ESI (Figs. S14, S15, S16) shows a series of decahedra DPs in comparison to cubooctahedra and icosahedra as well as comparison of Wiliamson-Hall plots for the first two (Fig. S13). Suggestion that small Au crystals can have quasicrystalline decahedral structure was supported by TEM observations presented in ESI.

**Figure 12:**
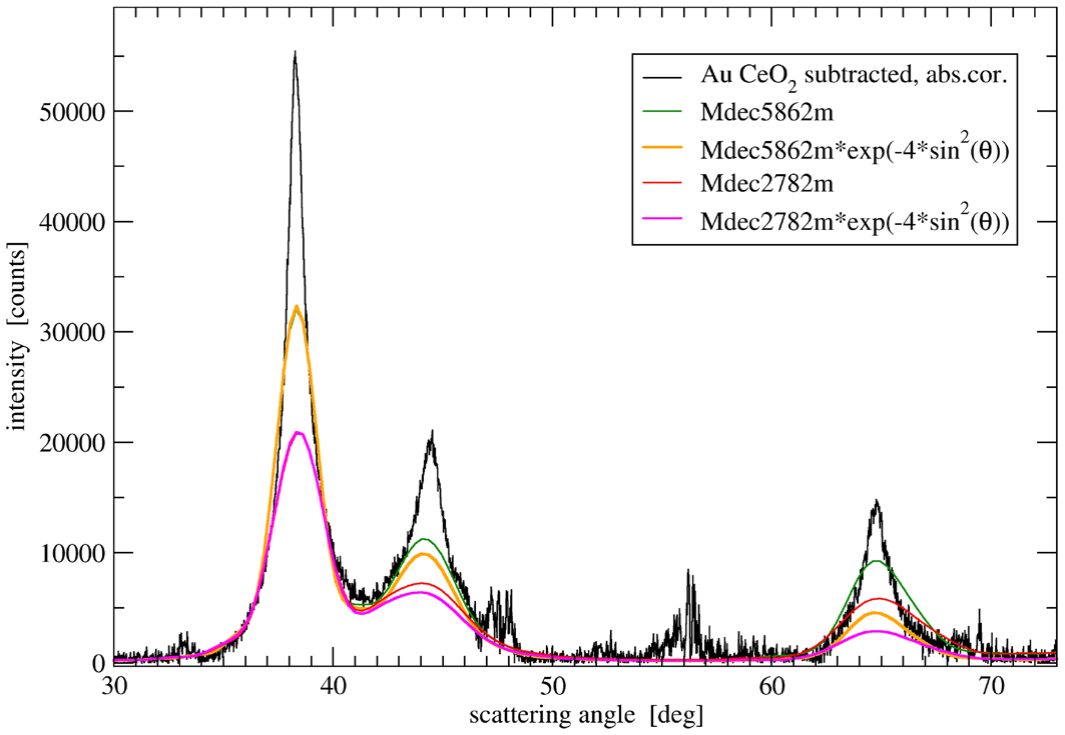
Background- and ceria-subtracted DP of gold phase corrected for sample absorption (black line) compared to two model DPs showing necessary degree of intensity dumping to achieve fit. The models are energy relaxed Marks decahedra.

Following the above line of reasoning we attempted to fit the Au DP profile with a number of model DPs in the wider available angular range. It encompassed three Au peaks (corresponding for fcc structure to 111, 200 and 220) as the further ones strongly overlapped with ceria peaks and could not be clearly determined. The attempt to fit in Fig. 12 shows necessity of the model intensities to be significantly attenuated using Debye-Waller like factor to consequently describe all three peaks of Au. The DWF is, however, correlated with sample absorption factor^70^, and the evaluated degree of peak attenuation has to account for correction for absorption of X-rays in the sample. This is why the subtracted Au DP of Fig. 12 has been corrected for the sample absorption as discussed in ESI.

The DP is composed of at least two components representing small crystallites (D < 5 nm) and large ones (D > 20 nm) responsible for narrower peak tops with positions already close to that of the FCC structure. The narrow peaks component shows decreased height and increased width of 200 peak characteristic also for large multiply twinned crystals. The broad peak component fitted to DP of Marks decahedra requires large DWF corresponding to the mean square atom displacement value of $\sim$ 0.18 Å$^{2}$. It points to a significant disorder of this component of Au phase. In atomistic simulations via Molecular Dynamics (MD) such value of DWF can be obtained at 700–800 K and is accompanied by signs of surface melting (see ESI). The fraction of larger Au crystals on the other hand, does not require strong DWF dumping and suggests less disordered structure. Ratio of peak intensities of this fraction is close to the model ratio for large Marks decahedra. ESI provides example of structure analysis for another synthesized sample Au/C (Figs. S1, S7). The sample luckily has DP very closely resembling the ceria subtracted Au phase of the ${\text{Au/CeO}}_2$ sample. It appeared to be practically inactive in CO oxidation but due to higher intensity and simpler background subtraction its fit to decahedra models supports with higher accuracy the above conclusions on Au disorder and surface mobility. Those properties do not seem, however, to be responsible for the catalyst activity. The overall Au DP of ${\text{Au/CeO}}_2$ can be fully reproduced by sum of model DPs as presented in ESI Fig. S23. The model fit is, however, arbitrary and barely proves ability of several theoretical cluster models to approximate the Au patterns obtained from the experiment. It was not refined and the model clusters were selected intuitively. However, the crystal size distribution resulting from the fit quite well agrees with that resulted from TEM and its volume weighted average agrees with the crystal size deduced from XRD.

## Discussion

We have shown experimental data from monitoring of gas atmosphere and DP during programmed physico-chemical treatment of the ${\text{Au/CeO}}_2$ catalyst at 150 °C. It involved conditions of CO oxidation reaction as well as conditions of preferential oxidation in the ${\text{H}}_2$-rich stream.

A supplementary ex situ characterization of the catalyst (TEM, XPS, TPR) is provided in ESI.

The literature on the mechanism of CO oxidation is abundant covering many recipes for the catalyst preparation and different reaction conditions. It is likely that the general mechanism is complex, involving many partial reaction steps, role of water and/or of carbonaceous deposits. Our conclusions concern structure evolution of our particular model catalyst designed specially to make various subtle effects detectable. All derived conclusions base on a long time sequence of measurements in each gas atmosphere and correspond to stationary state of the catalyst. It is then hard to explain the change of ceria LP in ${\text{H}}_2$ by ceria reduction knowing that it returns immediately to previous value in He atmosphere with no oxygen supply. Lack of the lattice oxygen exchange with the gas phase at our recorded temperatures was already shown in literature in isotope exchange experiments^16,17^.

Our analysis employs full diffraction data measured in situ with good statistical accuracy. This, together with characterization of the material by a range of techniques allowed deep insight into its atomistic structure and dynamics during the observed processes. Interpretation of these data strongly suggests that in our experimental conditions:

- lattice oxygen of ceria is not exchanged with a gas phase and the overall stoichiometry of ceria remains constant,
- gold supported on ceria activates hydrogen allowing its spillover and chemisorption causing charge transfer, formation of additional ${\text{Ce}}^{3+}$ ions, or migration of ceria lattice oxygen to the surface. The latter process follows for CO chemisorption on both - the catalyst and ceria alone,
- the supported gold has epitaxial contact with ceria and adopt quasicrystalline, highly disordered structure,
- Au atoms are likely to be highly mobile migrating to the surface of ceria or, depending on the chemical environment, in part migrating back to the gold crystal.

The outcome of this work gives two consistent observations derived from two independent analyses. Changes in the ALP of ceria suggest decrease of the ${\text{Ce}}^{3+}$ ion population or migration of oxygen vacancies from the surface to the grain interior at the same time when some gold atoms migrate onto the gold crystals increasing its diffraction peaks intensity. It clearly suggests that some of gold atoms weakly and reversibly anchor at ${\text{Ce}}^{3+}$ sites (next to oxygen vacancies), what was proposed as active site in DFT studies. The decrease in ${\text{Ce}}^{3+}$ population or redistribution of ${\text{Ce}}^{3+}$ towards the NPs core releases those Au atoms and forces them to temporarily build up the bigger AuNPs. The structure changes during catalytic reaction agree well with the mechanism suggested by Kim et al.^11^ assuming ${\text{Ce}}^{3+}-\text{Au}$ pairs as active sites and direct oxidation involving no ceria lattice oxygen - in opposition to Mars-van Krevelen mechanism. The gold mobility can be interpreted as formation of small unstable Au clusters bound to ${\text{Ce}}^{3+}$ ions in proximity of the surface oxygen vacancies. Significant gold transport phenomena of a catalyst supported on ceria modified with ${\text{Eu}}^{3+}$ during the CO oxidation reaction have been already suggested on the base of in situ Raman study^71^. Our work supplies similar conclusions based on structural method. We prove that in situ powder XRD can be refined to provide for nanocrystals deep insight into their surface dynamics.

Our results suggest complex and highly dynamic reaction picture non consistent with a concept of stable active sites. It can be further corroborated by DFT calculations of the effects of chemisorption on bonding stability of such small Au clusters.

## Methods

### Sample selection and diffraction pattern repeatability

The technique can be applied to nanocrystalline phase which has to be observed with good statistical accuracy. This is why we use decent amount of the sample and long time measurements. With nanocrystalline samples a resolution poses no longer problem as the instrumental peak broadening is by far smaller than size peak broadening. For the purpose of the study we successfully synthesized a series of catalysts with high Au loading and good dispersion. With high scattering power the gold nanocrystals in the size range 2–20 nm can be analyzed via diffraction and monitored during the in situ treatment. Monitoring of diffraction peaks during the catalyst exposure to various chemical environments as well as to CO oxidation reaction and PROX (preferential oxidation of CO in excess of hydrogen) reaction provides data sensitive to the surface state of gold as well as to subtle modifications of support structure (e.g. ceria). When a time scale of the monitored processes is long enough, the analysis concerns stationary states. To account for the support effect we compare experimental results for pure ceria and for nanocrystalline gold supported on a number of supports including that considered to enhance the catalyst activity (${\text{CeO}}_2$) as well as more inert (${\text{SiO}}_2$, C). The measured data following the chemical treatment point to small variations of a crystal overall LP being a consequence of the surface changes. Also absorption corrected evolution of the peak intensity points to the ordering or mass transport phenomena that have to be understood as a surface processes. This information can be extracted from a running chemical process applying right methodology. As the process monitoring comprises 5 DPs during each exposure of the sample to the gas environment, this sequence allows estimation of the repeatability and improves the average precision.

### Gas purity and camera tightness

For monitoring of the sample structure over long time in flow XRD reactor/camera one has to be warned against gas escape, has to control gas purity, and a possible reaction products. It is done using mass spectrometer with SEM detector covering partial pressures in the camera down to $10^{-6}$ atm. The available commercial pressured bottles may be contaminated with small amounts (at the level of ppm) of adsorbing gases e.g. oxygen. Simple calculation assuming the sample weight of 50 mg and flow of gas of 20 ml/min during 5 hours exposure, shows that for the measured ratio of surface to bulk atoms close to 0.1 the surface atoms amount to about $3 \times 10^{-5}$ mole and the possibly supplied oxygen is about $2 \times 10^{-7}$ mole for 1 ppm of oxygen or $2 \times 10^{-6}$ mole for 10 ppm of oxygen. In the last, the worse case it would cover only 10% of the available surface. As our gas sampling rate is one per minute, and DP sampling is one per hour, during 5 h exposure we should clearly record any LP evolution if the process happens. The higher contamination with oxygen would be spotted on the MS data measured with SEM detector (data deposited with the manuscript) sensitive down to $10^{-12}$ mbar. Typically atmospheric pressure pumped via capillary corresponds to slightly more than $10^{-6}$ mbar, thus $10^{-12}$ level corresponds to ppm of the camera pressure. Although this ppm level is noisy, the tens of ppm are detectable with a reasonable dwell time. The observed in the experiments LP sharp decrease in He after its rise in CO or ${\text{H}}_2$ cannot be thus caused by He gas contamination with oxygen. With similar setup we could register via diffraction chemisorption of oxygen on Pd nanoparticles and its subsequent desorption via shifting equilibrium in argon atmosphere or flushing with hydrogen^72^. Each process modifies nanocrystal surface contraction observed for metals and causes detectable peak shift. The purity of argon was comparable to that of He used in the current study and, during long exposure, no contamination of the surface by oxygen was observed.

### Absorption correction and temperature monitoring

The issues concerning X-ray absorption include absorption by gas within the camera and an angle dependent absorption by the sample. The first one can be effectively accounted for assuming gas composition and available mass absorption coefficients (International Tables for X-ray Crystallography) for the gas density dependent on temperature. At higher temperatures, for cylindrical camera one has to assume gas temperature radial profile (logarithmic) and integrate the correction along the X-ray path. Such correction works excellent when checked on inert samples with small surface when gas adsorption is negligible.

The X-ray absorption by the sample is usually described by Milberg correction^70^, that for a highly absorbing material like ${\text{Au/CeO}}_{2}$ is negligible for samples of thickness exceeding several tens of microns. When working with the sample smeared over porous glass mounted vertically, the effective sample thickness is usually less than that and the correction should be applied. It is evident when comparing the fitted Debye–Waller (D–W) factor with that measured for a thick sample. The D–W factor is correlated with Milberg correction (see ESI) and the resulting mean square atom displacement values can be quite different.

In the current study to monitor DP of gold we used high loading of Au supported on ${\text{CeO}}_2$ (9.4 wt% is a 3–100 fold excess comparing to literature reports, e.g.^73^). CO oxidation is a highly exothermic reaction ($\Delta H ^{298 \, K} = -283 \, {\text{kJ}} \, {\text{ mo}}l^{-1}$)^74^ and this is why the phenomenon called ”catalyst light-off”^75^ was always observed for this sample. During the catalytic reaction the sample temperature measured by thermocouple was always lower than that indicated by the quartz internal standard temperature expansion peak shift. The difference reached nearly 100 °C. Evidently, around the local heaters we face substantial temperature gradient. Simple calculation assuming perfect mixing of gases and our experimental conditions (see ESI) reveals that our catalyst works as a heater of a power of 2.44 W. Assuming that within the considered temperatures, the dominating heat transfer mechanism is convection, we can estimate its rate as $5 \times 10^{-4} \, {\text{W/(m}}^{2} \, {\text{K}})$. The heat conduction via the crystal lattice is by several orders of magnitude quicker than the gas phase mediated energy exchange. One phonon oscillation in crystal lattice lasts about $1 \times 10^{-13}$ sec. The gas phase atoms hit surface about $1 \times 10^9$ times per second. Even assuming the surface of the catalyst crystal as consisting of few thousand atoms, it should ensure temperature equilibration before onset of the convection cooling (see ESI). As gold is in epitaxial contact with ceria (as indicated by TEM data, see ESI Figs. S3, S4), the gas phase heat dissipation should depend on the overall surface of ${\text{Au/CeO}}_2$ and not on the Au surface (and loading) alone. The local temperature rise should depend mostly on CO and ${\text{O}}_2$ contents in the gas stream and not on the Au loading. Carrying CO oxidation with gas mixture containing 1% of CO and assuming convection from the whole catalyst surface should result in the temperature gradient 50 times less than that measured by us. However mechanism of the reaction is unlikely to be different at the temperature increased by 100 K and Au mobility is not caused by the local hot spots—as it was shown, the amount of gold diffusing to the support increases in He when there is no temperature rise.

## Supplementary Information

Supplementary Information.

## Data Availability

All data generated or analyzed during this study are included in this published article and its supplementary information files.
