# Supplementary material for: In situ diffraction monitoring of nanocrystals structure evolving during catalytic reaction at their surface
Source: Sci Rep. 2023 Jan 26;13:1469. doi: 10.1038/s41598-023-28557-5 (PMC9879985; doi:10.1038/s41598-023-28557-5)
Supplement: Supplementary file 1 — Supplementary Information. [file 41598_2023_28557_MOESM1_ESM.pdf]

Supplementary Information to "In situ diffraction monitoring of nanocrystals structure evolving during catalytic reaction at their surface " by M.Zieliński, Z.Kaszkur, W.Juszczyk, J.Sobczak.

Preparation of Au/CeO2 catalyst.

The study has been performed on the Au/CeO_2 catalyst prepared the following way. The reagents and materials used during the synthesis process are: cerium dioxide (CeO_2 , ppa, Austranal Praeparate), hydrogen tetrachloroaurate(III) trihydrate ($HAuCl_4 \cdot 3 H_2O$, 99.9%, Alfa Aesar), sodium borohydride ($NaBH_4$, 99%, Sigma-Aldrich), sodium hydroxide (NaOH, ppa, Chempur) and double-distilled water (also referred to as redistilled water). 20 ml 0.0254 M of tetrachloroauric acid solution diluted with 330 ml of redistilled water was vigorously stirred at 500 rpm in darkness. Under constantly monitored pH value which never exceed pH = 11, it was alkalized dropwise with approx. 51 ml of 0.05M $NaOH_{aq}$ and was left overnight under stirring. The initially yellow solution turned into transparent. This indicated the tetrahydroxyaurate ($Au(OH)_4^-$) complex was formed successfully. Then, 900 mg of CeO_2 which was suspended in 50 ml of redistilled water via ultrasonication aided by mechanical stirring was added to the tetrahydroxyaurate complex solution. The mixture was further stirred for 2 h at 80-85°C. After cooling the mixture down to 0°C, 58 mg of $NaBH_4$, dissolved in 20 ml of 0.05M $NaOH_{aq}$, was added dropwise. It resulted in immediate colour change into purple. The mixture was subsequently stirred for another 12 h in room temperature. Then, the catalyst was filtered and rinsed 15 times with redistilled water until no chlorine anions were detected. It was finally dried overnight under vacuum in 40°C. A ready-for-use catalyst was stored in darkness in a desiccator. The Au contents as determined by XRF (MiniPal4, Panalytical) was 9.4% wt.

Besides Au/CeO2, the following samples were used as a reference :

1. Au/C

The 20%wt. Au/C catalyst synthesis recipe originates from J. Turkevich method [1] appropriately modified according to K. C. Grabar [2]. This particular method, which allowed to obtain stable gold colloids with gold crystals of the mean size around 5 nm, was successfully applied to the synthesis of gold based catalysts by N. Kristian et al. [3] and E. Frota [4].

The following reagents and materials were used during the synthesis process: carbon Vulcan XC 72 (Cabot Corporation), hydrogen tetrachloroaurate(III) trihydrate ($HAuCl_4 \cdot 3 H_2O$, 99.9%, Alfa Aesar), sodium borohydride ($NaBH_4$, 99%, Sigma-Aldrich), trisodium citrate dihydrate ($Na_3C_6H_5O_7$, ppa, Chempur), propan-2-ol (ppa, Stanlab) and double-distilled (redistilled) water.

In order to prepare 500 mg of the catalyst, 400 mg of carbon was suspended (ultrasonication aided by mechanical stirring) in 85 ml mixture of propan-2-ol and water (volume ratio 3:1). 20 ml (0.0254 M) tetrachloroaurate solution was diluted under vigorous stirring (~600 rpm) at room temperature with 1725 ml of water and then 17 ml of 4%wt. trisodium citrate solution was added. In a separate beaker a reducing agent solution was prepared. It consisted of sodium borohydride (15 mg) dissolved in 4% wt. aqueous trisodium citrate (17 ml). Addition of the reducing agent solution to citrate-protected tetrachloroaurate resulted in immediate colour change to deep red which is characteristic for gold colloids containing approx. 5 nm particles. After 5 minutes, the carbon suspension was purged into the red gold colloid. The mixture was stirred for 48 h in darkness. Then, it was filtered and rinsed with water until no chloride anions were detected. Finally, the catalyst was dried overnight under vacuum in temperatures not exceeding 40°C. A ready-for-use catalyst was stored in darkness in a desiccator.

2. Au/SiO_2

The 7.16%wt. Au/SiO_2 catalyst (composition assessed by XRF measurements) synthesis recipe was based on the idea of Y. L. Lam and M. Boudart [5] which was applied so that only gold containing catalyst was obtained (originally Au-Pd alloyed catalyst's active phase was obtained). Gold precursor was prepared according to original work by B. P. Block and J. C. Bailar [6] which was re–examined by W. J.Louw and W. Robb [7] and optimised by K. Kitada [8].

The following reagents and materials were used during the synthesis process: hydrogen tetrachloroaurate(III) trihydrate ($HAuCl_4 \cdot 3\,H_2O$, 99.9%, Alfa Aesar), 1,2-ethylenediamine ($H_2NCH_2CH_2NH_2$, will be abbreviated by "en", ≥99.5%, Roth), silica (SiO_2 silica, Davison 62), diethyl ether ($(CH_3CH_2)_2O$, will be abbreviated by "Et_2O", ppa, Chempur), propan-2-ol (ppa, Stanlab), 25% ammonia solution (NH_3 aq, ppa, POCH) and double-distilled (redistilled) water.

To prepare the gold precursor - di(ethylenediamine)gold chloride ($Au(H_2N(CH_2)_2NH_2)_2Cl_3$ = $Au(en)_2Cl_3$) solutions of 1 g of hydrogen tetrachloroaurate(III) trihydrate in 10 ml of diethyl ether and 1 ml of 1,2-ethylenediamine in 5 ml of Et_2O were mixed. A gummy yellow precipitate was formed. Following 30 min of refluxing, the solvent was evaporated under diminished pressure. An orange solution of the product was obtained after addition of approx. 10 ml of water. Following addition of approx. 90 ml of propan-2-ol a yellowish precipitate was formed and subsequently filtered. Crystallization from water and propan-2-ol mixture was repeated resulting in white powder which was dried overnight under vacuum at 40°C. Recrystallization was applied every time before using the gold complex in catalyst's synthesis.

All subsequent steps were performed in darkness. In order to prepare 1000 mg of the catalyst, 900mg of SiO2 silica was suspended in 0.4 ml of 25% ammonia solution diluted with 50 ml of water resulting in pH~11. The mixture was stirred at 500 rpm. 215 mg of fresh di(ethylenediamine)gold chloride dissolved in 50 ml of water (transparent solution) was added dropwise to silica suspension. The slurry was heated up to 70°C. Following 1 h when the temperature never dropped below 68°C, the mixture, which turned into yellow, was filtered under diminished pressure, rinsed 10 times with water and dried overnight under vacuum. A yellow powder was obtained.

Before performing any experiments, the catalyst was activated in 20 ml/min flow of hydrogen for 80 min in 50°C, then 160 min in 100°C and finally 400 min in 150°C. The activation procedure aimed at reducing the gold cation complex anchored to SiO_2 surface.

The experimental setup.

The setup consisted of a U-shaped glass reactor with a porous sintered glass disk where a thin (~1 mm) catalyst bed was formed. The design of this non-fluidized bed reactor provided uniform flow of gas through the bed and thus uniform performance of the whole loaded sample. Therefore, model of a differential Plug Flow Reactor (PFR) could be assumed.

The reactor was heated by a tube furnace controlled by its inner thermocouple. Another thermocouple indicated the temperature of the catalyst bed. Some deviations between the temperatures of the furnace and the catalyst bed were experienced, so any references are always made to the temperature of the sample. At the inlet of the reactor there was a nickel carbonyl ($Ni(CO)_4$) trap made of stainless steel cylinder tightly filled with steel mesh, which improved heat transfer. It was heated up to 200°C or more in order to decompose $Ni(CO)_4$ and to avoid catalyst poisoning by the nickel deposits as well as to avoid the possible influence of chemical properties of nickel on the monitored chemical performance of the studied sample. All parts of the setup, between the Ni-Trap and inlet of the mass spectrometer (MS), which were placed outside the tube furnace, were heated by a heating mantel. Its major role was to prevent condensation of any vapours (e.g. H_2O which might be a side product) in the tubes before

the gas stream reached the MS inlet and the vapor condenser afterwards. Formation of droplets could be harmful for the MS ionization, for the quadrupole chambers or for the turbomolecular pump and could cause undesirable spikes on MS spectra being the result of oversaturation of Faraday cup or Secondary Electron Multiplier (SEM) detectors.

Gas stream was supplied from a separate vacuum–gas system, which was used as a source of gas for other experimental setups as well. Briefly, the vacuum-gas system allowed supply of the setup with a gas stream of desired composition, which was adjusted by two mass flow controllers (MFC). MFCs were connected to two ducts which allowed to choose the proper gas from the pressurised bottle. Ducts were evacuated 3 times when a change of gas was needed. This allowed a quick and efficient gas exchange.

The chamber was equipped with removable aluminum cap that had a Kapton™ window which allowed for crystal structure studies by means of XRD. There was a Viton™ gasket fitted between the cap and basement to ensure air-tight closing. The basement was a stainless steel plate with several other parts and fittings soldered to it. The most important piece was the heating block which acted also as the sample holder and outlet for the gases from the chamber. Catalyst sample was spread onto and pressed into a sintered porous glass plate, and formed a thin layer on the plate. The glass disk was mounted on the heating block so that it covered the gas outlet which was embedded in the block. Right next to the chamber's outlet there was the inlet to the MS probing capillary via 3-way connector (see fig.1). Due to such arrangement, all alternations in the gas stream composition could be instantly detected by MS. Before the chamber's inlet, there was a Ni-Trap. Identically to chemical reaction setup, it was heated to at least 200°C and it prevented catalyst poisoning by decomposing $Ni(CO)_4$.

Tubing and manual valves were the Swagelok stainless steel parts. The vacuum-gas supply unit was equipped with an oil pump, pressurised gas bottles, pressure gauges by InstuTech Inc., electromagnetic on/off valves by REGADA and MKS® mass flow controllers (MFC). MFCs were calibrated using a bubble meter of 5 ml or 10 ml volume. Apart from gas bottles, all parts are operated automatically by the computer script according to the programmed sequence. Data was stored on a network attached storage (NAS).

Transmission Electron Microscopy.

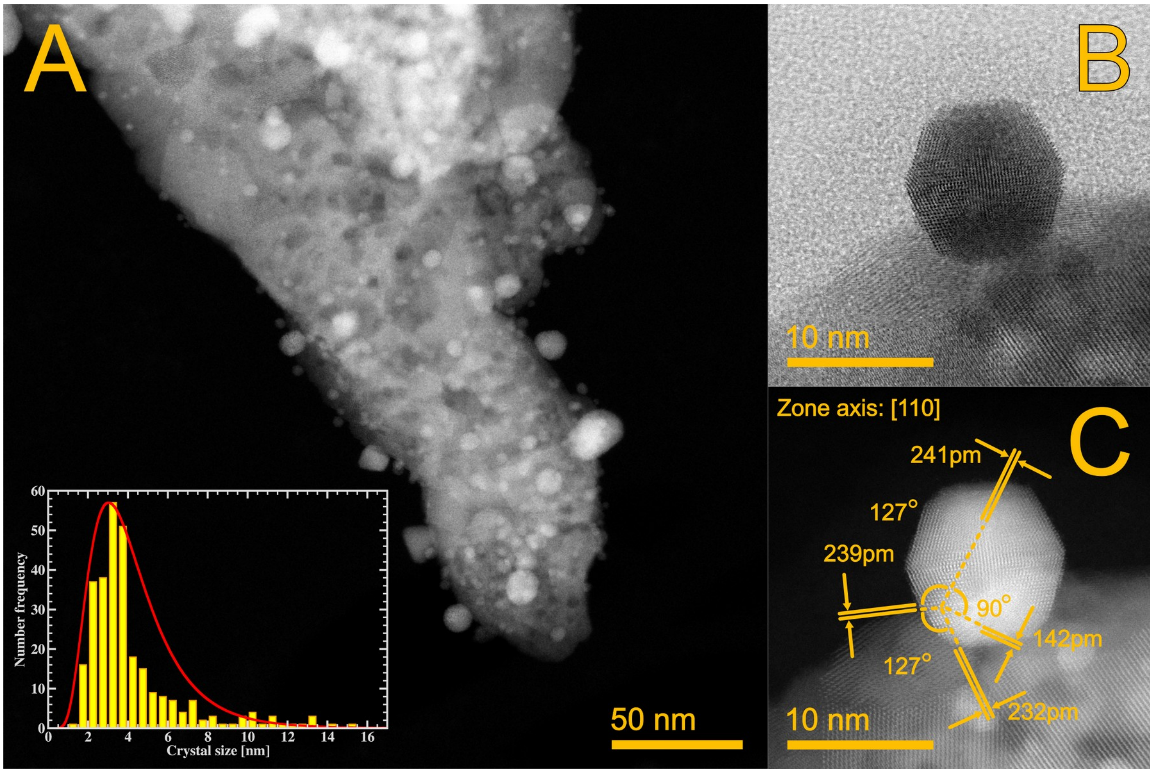

Fig. S1. A dark field STEM overview (A), bright (B) and dark (C) field STEM image of an AuNP deposited on the surface of CeO_2. The inner structure projection is characteristic for an ”edge”-oriented icosahedral quasicrystal [27-34] . The inset shows histogram of the gold nanocrystals size distribution in the measured HR TEM images and the log-normal function fitted to the experimental data. The X axis has been intentionally cut at 17 nm to improve the graph legibility - there were just a few single nanocrystals with bigger diameter spotted in the analysed set of images.

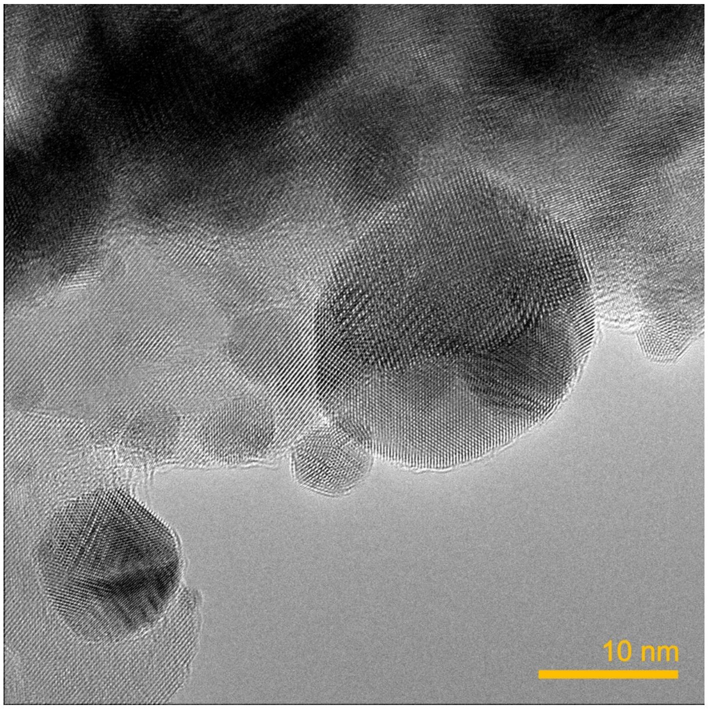

Fig.S2. Illustration of morphology of the Au/CeO_2 catalyst as well as of distribution of Au NP with diameters starting from 2 nm and not exceeding approx. 25 nm.

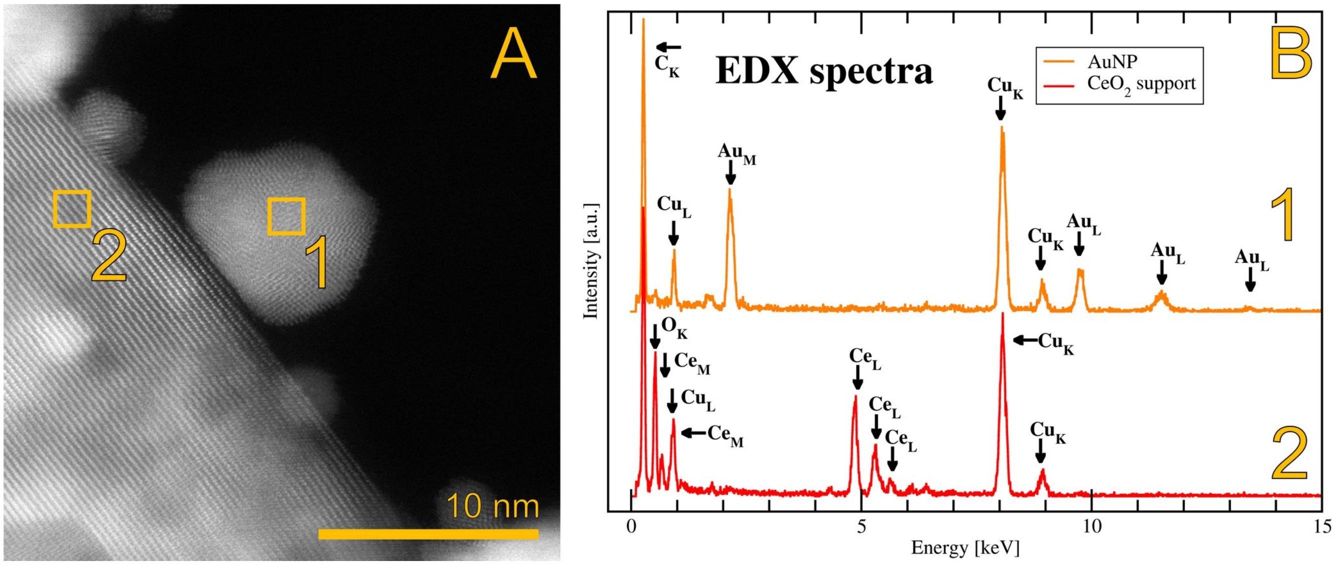

Fig. S3. A STEM dark field image (A) of a AuNP (place 1) attached to the CeO_2 (place 2) surface. The elemental composition was confirmed by the EDX spectra (B) collected from places 1 and 2.

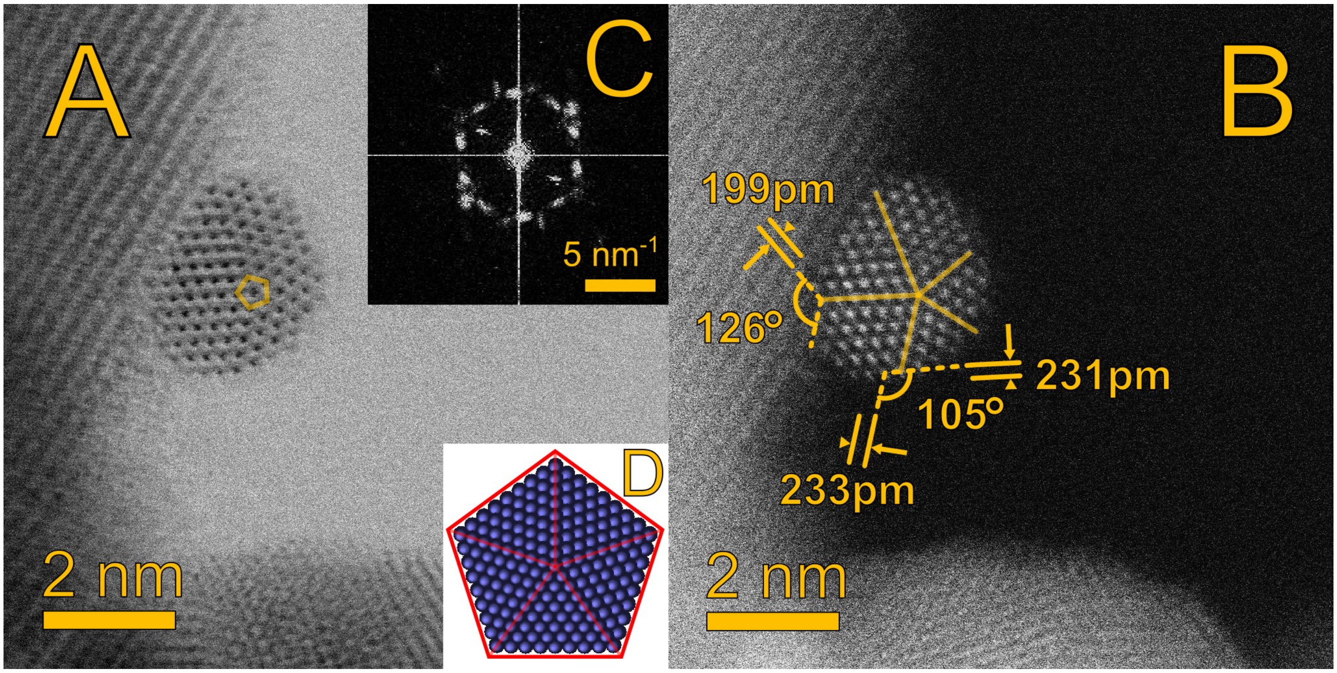

Fig.S4. The bright (A) and dark (B) field STEM image of an AuNP deposited on the surface of CeO_2 . The inner structure projection is characteristic for a decahedron with the 5-fold symmetry axis oriented perpendicularly to the image plane [65, 66, 68, 69, 73–75] .

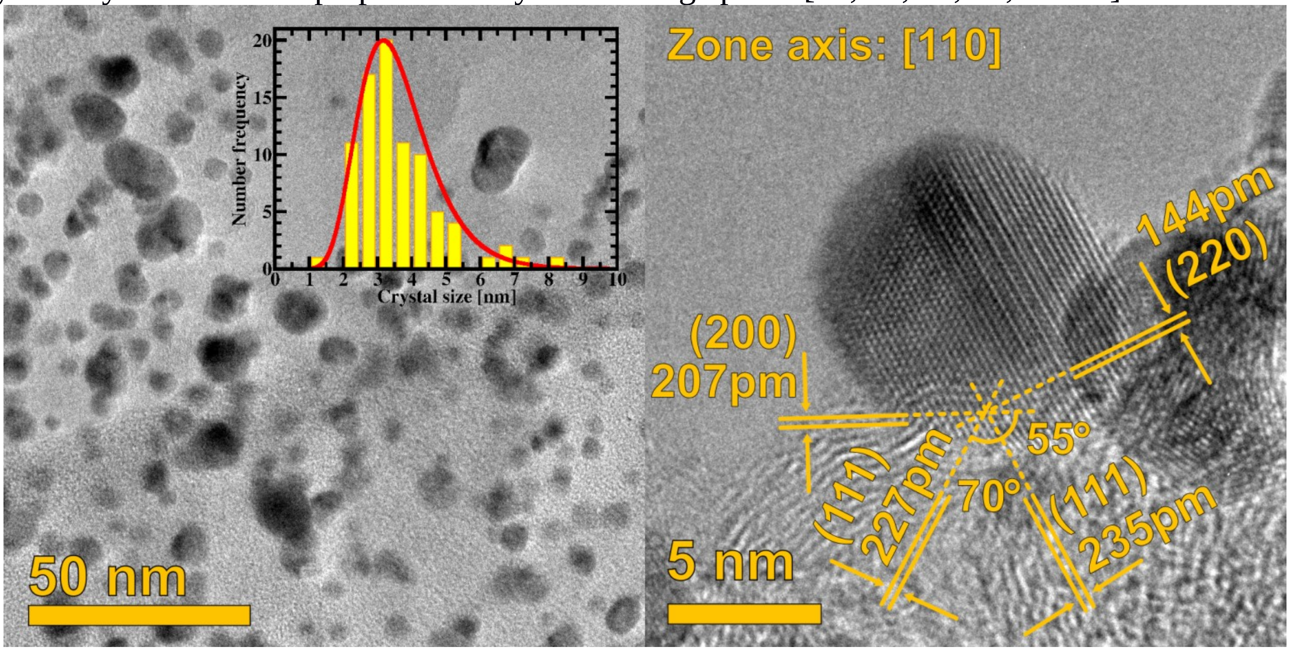

Fig.S5. Transmission Electron Microscopy image of a sample of Au/C. Average crystal size from XRD evolved during experiments from 7.3 to 8.7nm but visible is fraction of smaller crystals..

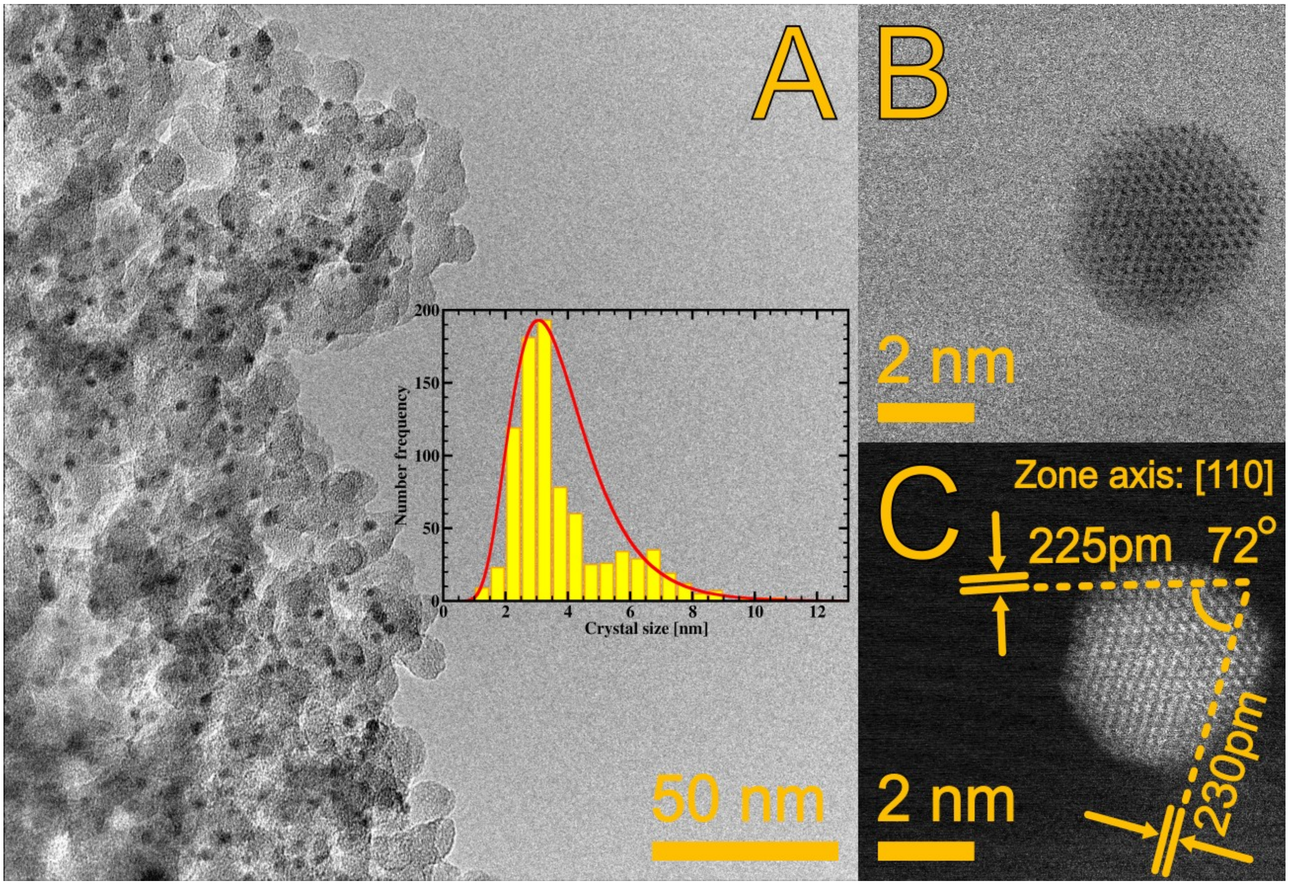

Fig.S6. TEM image of Au/SiO_2 sample. Average crystal size from XRD evolved from 2.6 to 4.3nm. TEM shows narrow size distribution.

Ex-situ TEM studies were conducted on FEI Titan Cubed 80-300 microscope operating at 300 kV, equipped with Energy Dispersive X-Ray Spectrometer for elemental analysis. Powder samples of the catalysts were dispersed in H_2O using ultrasonic bath. Then TEM Cu grids (300 mesh) covered with amorphous holey carbon film were immersed in catalysts' suspensions and left to dry on air. After the grid was mounted in the sample holder, it was pumped under ultra high vacuum (UHV) for at least 0.5 h, preferably overnight. No plasma cleaning was applied in order not to affect fragile Au NPs.
The TEM studies showed gold nanocrystals uniformly distributed on the CeO_2 nanoparticles surface. The vast majority of the observed Au NPs were smaller than 8 nm in diameter (Fig. S1). The biggest population of Au NPs were those with size between 2 and 4 nm (Fig. S2). However, a few AuNP reaching the size approx. 25 nm were also spotted. This fact corresponds well with XRD peak width and shape which is a volume-dependent feature.
The Au and CeO_2 phases can be easily distinguished in the TEM images, because their crystals differ in size and diffraction contrast, but to ensure certainty, an elemental analysis was frequently performed. The EDX spectra (Fig.S3) proved that the phases are purely made of gold or cerium and oxygen.
The most interesting observation concerned the inner structure of Au NP. They often were identified to be quasicrystals – also known as Multiply Twinned Particles (MTPs)- namely icosahedra and decahedra. Icosahedra can be preferred at temperatures lower than that of our in situ experiments where decahedra dominate [27] . In the Fig.S1 the gold icosahedron is visible in the ”edge”-orientation. Next to the ”top”-oriented icosahedron, these were the most feasible orientations to identify icosahedral shape of Au NP.

The second frequently observed quasicrystal shape of Au NP was decahedron presented in Fig.S4. Here it is seen along the only one 5-fold symmetry axis (comparing to 6 such axes in the icosahedron) which is the only orientation proving unambiguously the decahedral shape of the analysed particle. If such a particle is tilted to a side, it usually resembles a twinned crystal with the FCC structure. The truncated decahedron can even mimic the FCC structure with the [111] zone axis, if its 5-fold axis is in the plane of the TEM image and the whole particle adopts a proper position around this preferred axis. Although numerous gold MTPs were spotted during the TEM imaging, it remained impossible to estimate how big are the populations of icosahedra, decahedra and common FCC structure nanocrystals.

Catalytic performance of the empty reactor.

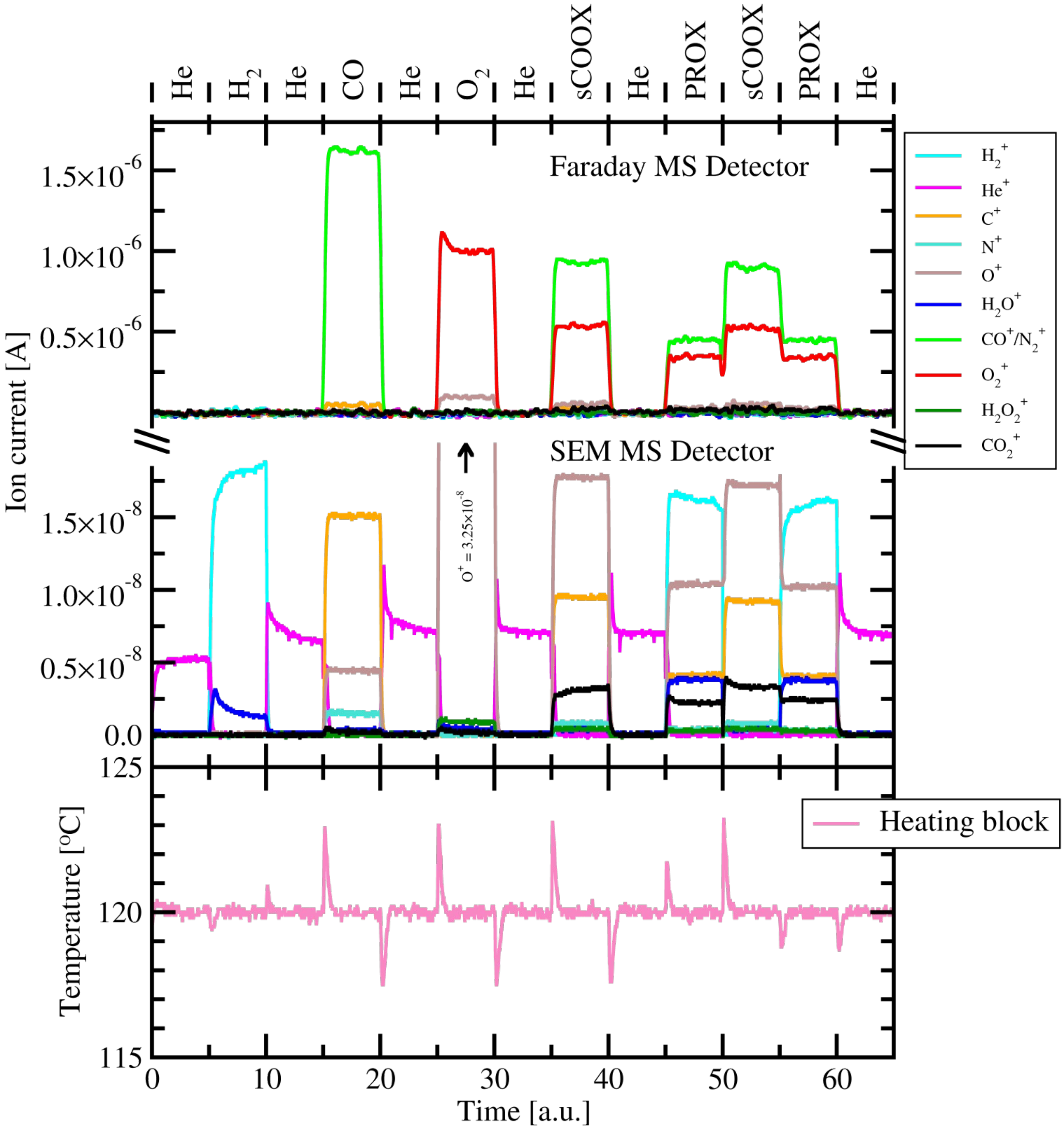

Fig.S7. Mass spectrometry (MS) analysis of composition of the gas stream leaving the PXRD chamber with CeO2 pressed onto sintered glass disk and the actual temperature of the specimen bed. MS signals corresponding to relevant cations are shown. Data are plotted against time normalized to time required to acquire one diffraction pattern (DP; approx. 42 min).

Powder Diffraction patterns of samples

Diffraction patterns of the samples are given in fig.S8.

Fig.S8.

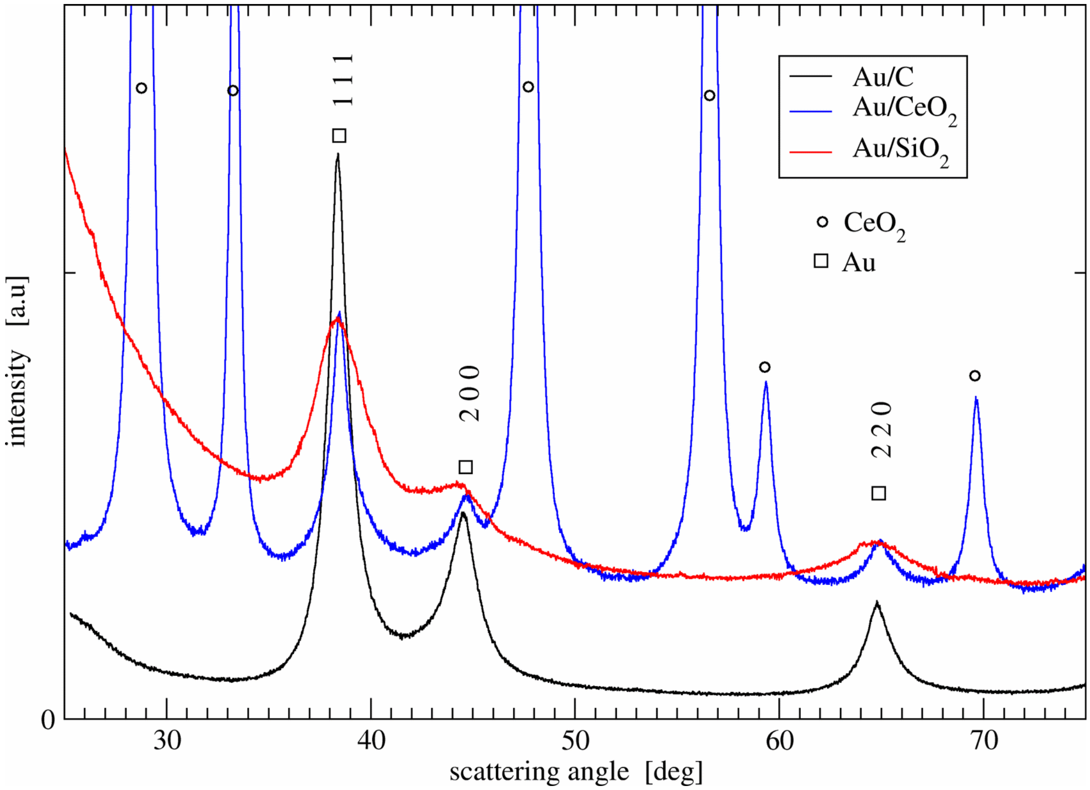

Diffraction patterns of the studied samples (ex-situ, Bragg-Brentano, Cu K_α).

Powder diffraction background estimation and peak fitting.

Rough fitting to Voigt functions (with arbitrary background guess) of the CeO_2 peaks in Au/CeO_2 XRD pattern (fig.S9) and estimation of integral peak widths reveals fairly flat Williamson-Hall plot. We can thus justly assume that microstrain plays insignificant role in the CeO_2 peak broadening. It allows us to model XRD peaks with Voigt function having shape parameter of 1.3 or larger, following [9]. For more detailed background subtraction we have estimated background level beneath each fcc peak of CeO_2 using criterion of the best fit to Voigt function described in [9]. Then the background points were interpolated with smooth function representing the real background to be subtracted from the experimental pattern. The Voigt functions used were doublets of Voigt peaks representing $K_{\alpha1}$ and $K_{\alpha2}$ components of Cu K_α spectral line. The background subtraction was followed by peak fitting (Fityk 1.3.0) resulting in separation of the scattering intensities from gold alone (assuming negligible contribution from Au-CeO_2 distances).

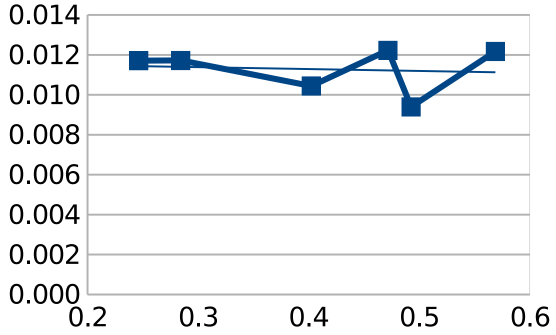

Fig.S9. Williamson-Hall plot peakwidth*cos(θ) vs. sin(θ) for 6 peaks of CeO_2

Background subtraction for the Au/SiO_2 sample involved subtraction of the SiO_2 support pattern. The absorption correction for silica alone and for silica mixed with Au is however different . We have assumed analytic form of it after Milberg [10] and the figure below shows correction of the silica pattern assuming range of absorption coefficients multiplied by the sample thickness. As the sample was spread over porous glass with the layer thickness difficult to estimate we considered a range of a likely values (blue and green curves in the figure below).

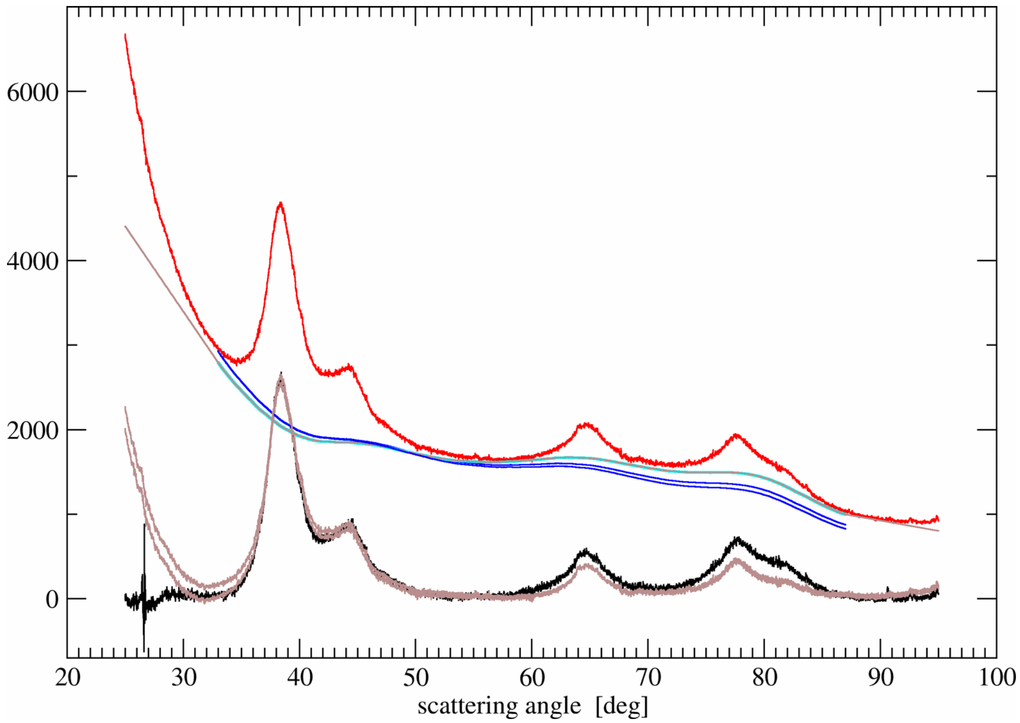

Fig.S10. Background subtraction for sample Au/SiO_2. The background lines correspond to silica XRD pattern corrected after Milberg , applying range of likely values of sample layer thickness (brown lines).

As can be seen, the silica pattern displays marked oscillations in proximity of the gold fcc peaks affecting intensity and shapes of the Au peaks after subtraction. To illustrate this effect a black curve at the bottom of the figure shows guessed Au pattern assuming regular smooth background.
The background subtraction for Au/C sample has been done following different strategy. Due to very high contrast in scattering power of C and Au, no maxima from carbon were noticeable and 10 turbostratic band cannot affect profile of 111/200 peaks. From fig.S8 one can notice that 004 peak expected at about 54 degrees is also absent. As carbon peaks were not well visible between the Au peaks we assumed that the pattern is shaped mostly by strong Au scattering and high absorption A(θ).

If Au-C pairs play minor role in the measured intensity then: $I_{total}(\theta) = A(\theta) \cdot I_{Au}(\theta) + A(\theta) \cdot I_{support}(\theta)$, i.e. the same absorption correction has to be applied to scattering intensities from both phases, where:

$I_{total}(\theta)$ – total observed experimental intensity;
$I_{Au}(\theta)$ – contribution originating from pure gold phase;
$I_{support}(\theta)$ – contribution originating from pure support phase;
$A(\theta)$ – sample absorption correction.

Hence, the gold contribution could be extracted as follows:

$A(\theta) \cdot \left(\frac{I_{Au}(\theta)}{I_{support}(\theta)} + 1 \right) = \frac{I_{total}(\theta)}{I_{support}(\theta)}$, i.e. the smooth line linking areas between gold peaks (where $I_{Au}(\theta) \approx 0$) on the ratio of the intensities corresponds to $A(\theta)$. After determining it one can easily recover $I_{Au}(\theta)$.

The same procedure applied to Au/SiO_2 results in Au pattern equal within the experimental error to the subtracted pattern from fig.S10 (brown line).

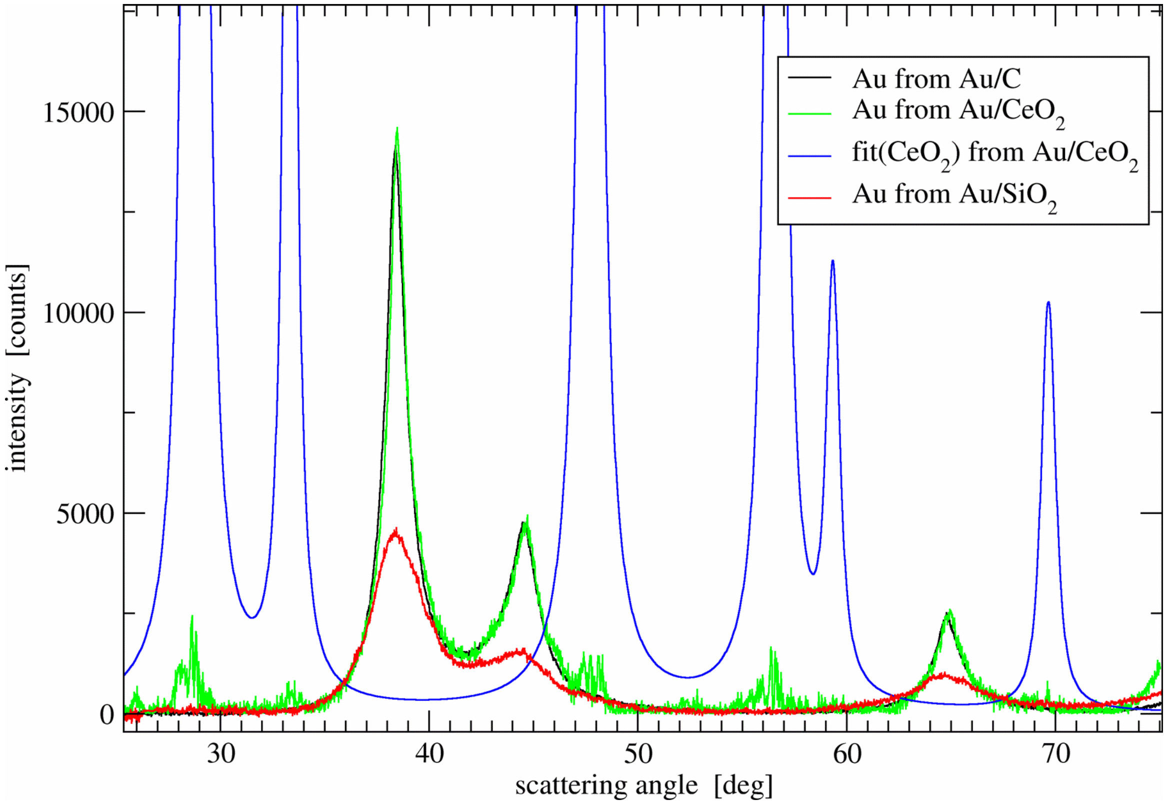

Fig.S11. Subtracted support diffraction pattern from XRD of all three studied samples.

The comparison of the subtracted patterns of Au for the studied three samples is presented in fig.S11. Notably the subtracted pattern of Au from Au/CeO_2 sample (green line with residual misfit intensities from the ceria) coincides very well with the pattern of Au supported on C (black line).

Note that the relative ratio of 111/220 is similar for Au/SiO_2 and Au/CeO_2 and, as it is mostly attributed to thermal-like disorder, it justifies correctness of the Milberg corrected silica pattern taken as the background (fig.S10).

Atomistic modeling of Au nanoparticles

The gold patterns were compared to a number of theoretical models encompassing cubooctahedra, icosahedra and decahedra of size ranging from several hundreds to 30000 atoms. The decahedra

models were Marks decahedra [11] that are energetically favorable and all models were energy relaxed using Sutton-Chen n-body potentials [12]. The structure models were built, relaxed and XRD patterns calculated applying Debye formula, using developed by us program Cluster [13].

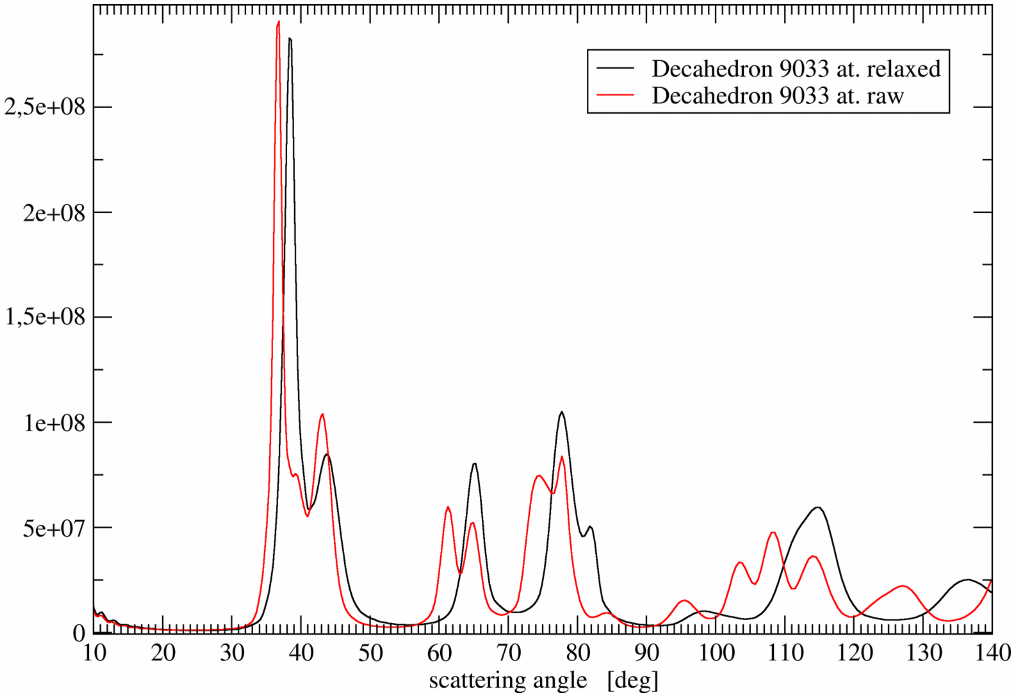

Fig.S12. Comparison between powder diffraction pattern of 9033 atom decahedron as constructed (red line) and energy relaxed (black line).

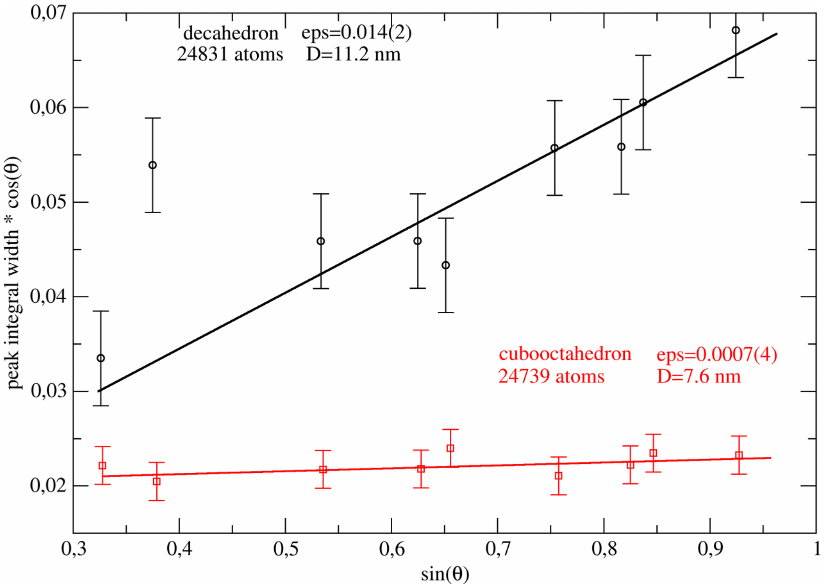

Models of fcc Au crystals can be constructed as a rigid fragments cut off the crystal lattice, The models of icosahedra and, especially, decahedra can be constructed (assuming 5-fold symmetry) only approximately. Arriving on a reasonable representation of their powder diffraction pattern requires energy relaxation to correctly distribute strain appearing between multiply twinned parts. An importance of such procedure is illustrated above on example of 9033 atom decahedron. After relaxation the pattern resembles

Fig.S13. Williamson-Hall plot for XRD peaks of decahedron and cubooctahedron of comparable size.

that of fcc crystals with systematic differences concerning peaks ratios, width and some deviations from Bragg law.

The presence of strain can be visible e.g. on Williamson-Hall plots. It is clear that length of the ordered rows of atoms is smaller in decahedron, ending up on the twinning planes. Nevertheless the method suggests the size that may significantly differ from that of the comparable size cubooctahedron and depends on the number of analyzed peaks.

Decahedra show some residual strain but still small in practical scale.
The decahedron peak positions deviate from the perfect fcc Bragg angles in a systematic way. It can be illustrated on the example of 111 200 pair of peaks (as indexed for fcc). Fig.S14 shows XRD profiles (intensity divided by the number of atoms) for a range of the relaxed Marks decahedra model sizes with peak positions marked. Approaching infinite sizes the peak positions approximate well Bragg fcc angles. For comparison an analogous figure is presented for the relaxed cubooctahedra models – fig.15. The legend lists the number of atoms in the model.

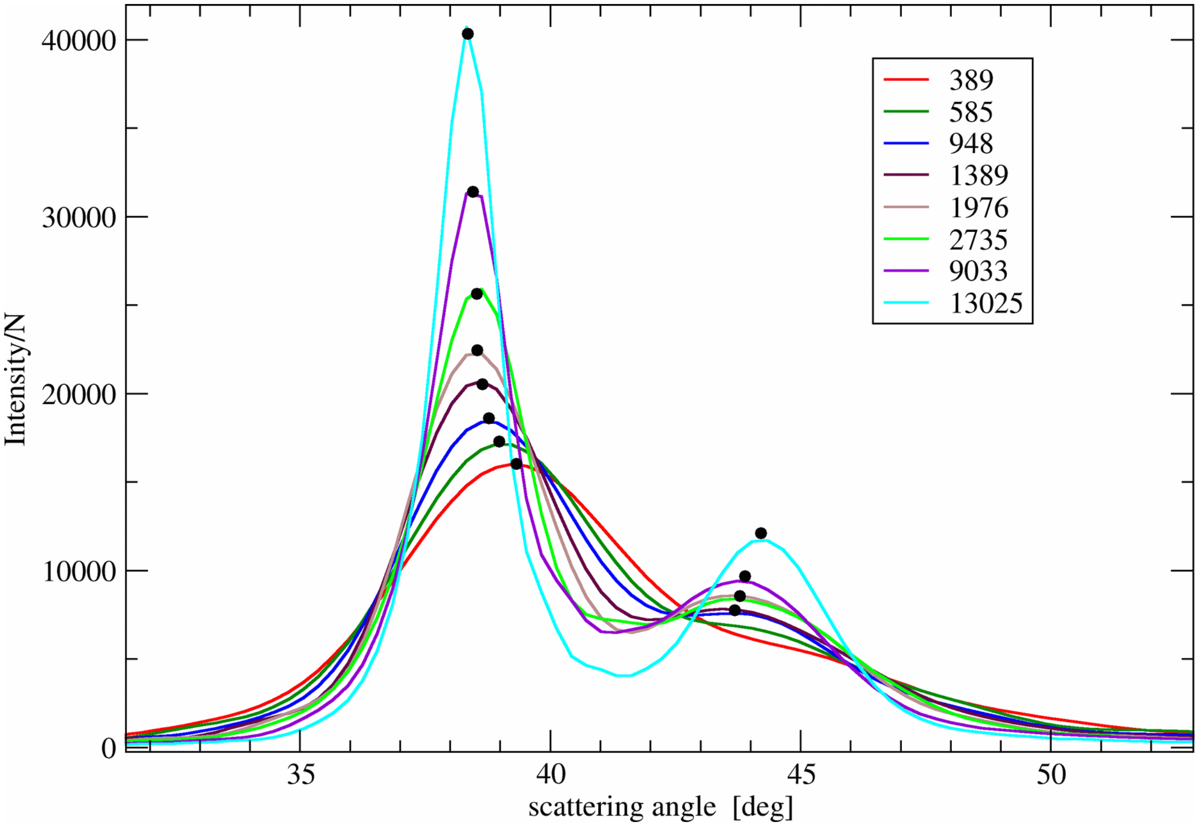

Fig.S14. 111 and 200 powder diffraction profiles for a number of model Marks decahedra.

Powder diffraction patterns of icosahedra (fig.S16) differ markedly from that of cubooctahedra (fig.S15) and decahedra (fig.S14). Increasing the size above 2 nm (400 atoms) the principal maximum at about 38 degrees gains a right hand side shoulder, further (above 6 nm, 8000 atoms) separating into distinct peak at 40 deg. All other peaks display variation in shape and position only roughly resembling their fcc counterparts. Within the Sutton-Chen potential scheme the icosahedra for sizes over 147 atoms (1.5 nm) become less stable than cubooctahedra and decahedra. The last ones have more advantageous cohesive energy per atom than cubooctahedra even for sizes 10 nm. The fcc structure regain advantage for very large sizes of crystals.
Except the structure relaxation, to fit models with the experimentally extracted patterns we ran the molecular dynamics (MD) routine at high temperature approaching 800K. It served purpose of describing experimentally evident disorder assuming it is analogous to the thermal one. The used Sutton-Chen potentials have a convenient feature of being scalable i.e. the energy results do not change if all interatomic distances (R_{ij}) change with the same ratio as the lattice parameter (a). The used starting lattice parameter was that reported for room temperature. The results of MD have thus to be scaled down to the lattice parameter a_0/a_{RT} (where a_0 corresponds to 0K temperature). Referring this result to disorder at experiment temperature (T_{exp}) requires further scaling by $a(T_{exp})/a(800K)$. The

corresponding diffraction pattern has thus to be modified scaling $\sin(\theta)$ inversely to the change of a (conserving intensity in Debye equation being function of $\sin(\theta)R_{ij}$).

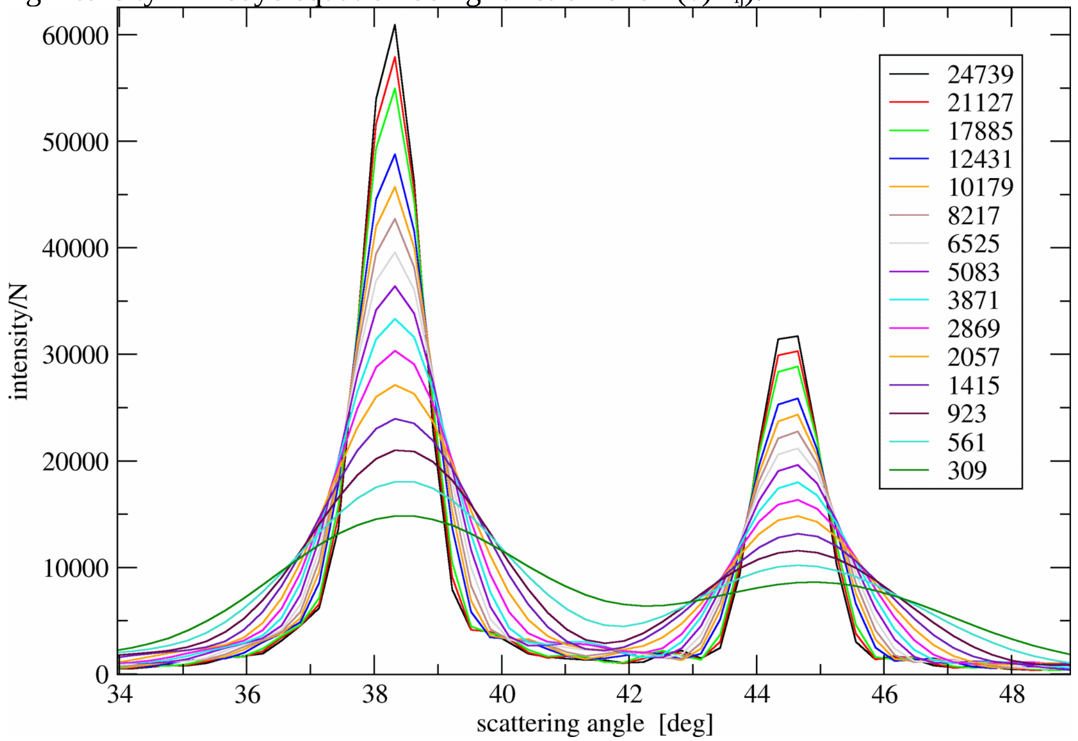

Fig.S15. 111 and 200 powder diffraction profiles for a number of model fcc cubooctahedra.

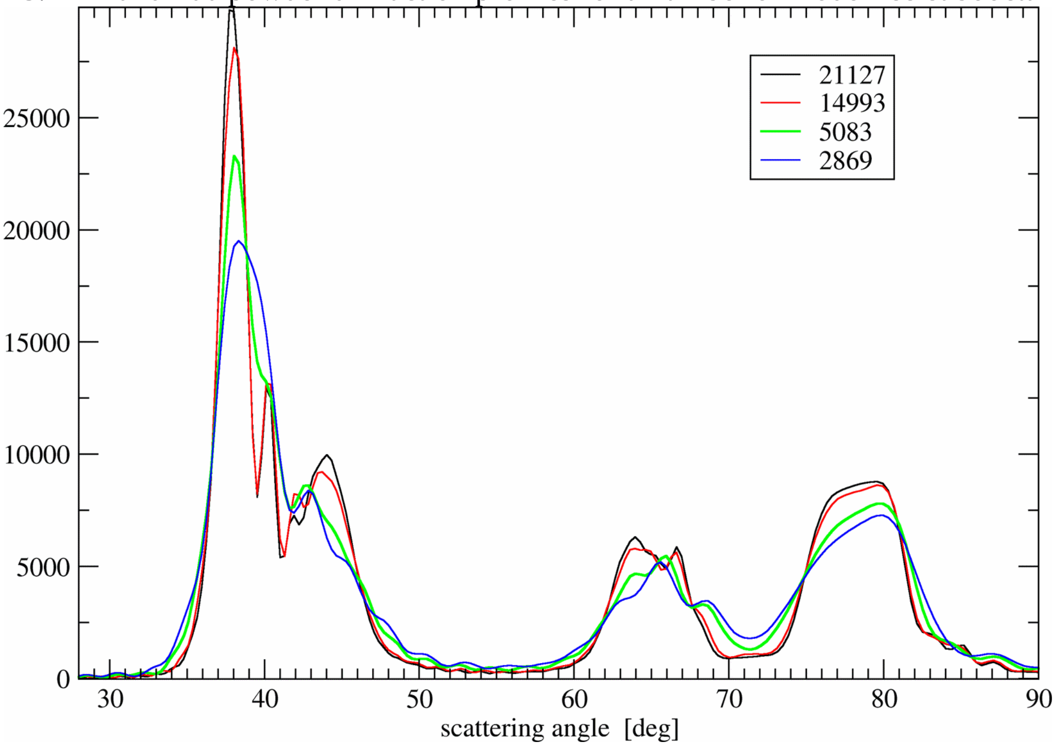

Fig.S16. Powder diffraction patterns of relaxed Au icosahedra.

Although the choice of interatomic potentials was somewhat arbitrary (Sutton-Chen) it provides the same minimum energy structures as other n-body potentials based on the second moment approximation to tight binding theory (EAM like potentials), known to describe well cohesion in metals (e.g. embedded atom model - EAM, Gupta potentials, RGL potentials [14,15]). Different EAM-

like functional forms differ mostly in surface energies, elastic constants or melting temperatures, and the minimum energy structures and the pattern of thermodynamic disorder weakly depend on the potential model used. Its temperature dependence can be however strongly dependent on a shape of the potential repulsive part that is different in different models. To illustrate the disorder effects generated by MD procedure at 800K the figure below displays comparison of diffraction pattern (as observed for CuK_{α} radiation) of a range of Marks decahedra energy relaxed and MD thermalized at 800K. The MD was ran in microcanonical ensemble (step 1 fs) with temperature scaled repeatedly to 800K and the cluster thermalized in 2000 steps.

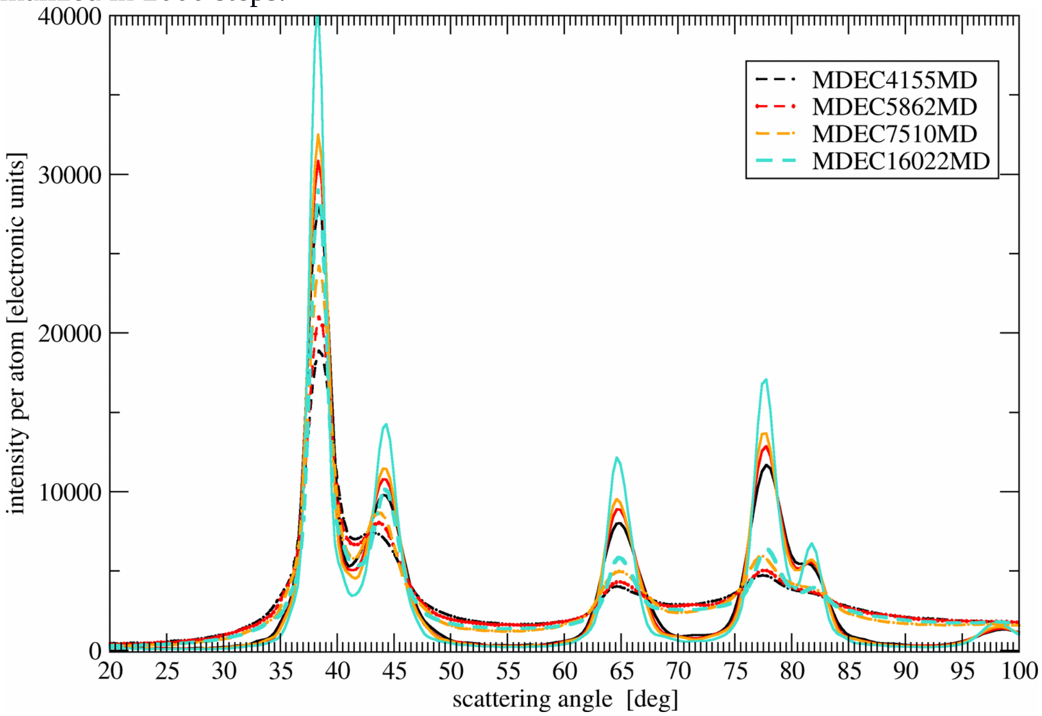

Fig.S17. Comparison of diffraction patterns of energy minimized (full line) and MD thermalized at 800K (dashed line) Marks decahedra (MDEC) built of varying number of atoms.

The figure S17 shows peak attenuation as well as rise of the background with scattering angle which is in qualitative agreement with a classic description of Debye-Waller effect. However, a closer look reveals deviations from the classic picture. Fig.S18 gives an example comparison of the classic DW effect and the simulated one for 7510 atom Marks decahedron. It is clear that the peak background cannot be described by $F^2(\theta)(1-\exp(-B*\sin(\theta)^2))$ function like in classic DW description – the background has a local maximum between 220 and 311 peaks (~70° 2θ). As the experimental background subtraction would include the smooth background originating from atom thermal motions, the figure S18 compares two background subtracted patterns. It is clear that the MD pattern shows marked broadening of most peaks (except 220) and the rate of attenuation disagrees with the DW model. The 220 peak makes an exception from average rules as the [220] direction overlaps with the 5 fold symmetry axis of decahedra. The peak broadening as a result of MD generated disorder is much smaller for a non-twinned models (e.g. cubooctahedra) and suggests that the perfect model description of the experimental pattern involving decahedra should underestimate the model size. An example below shows this effect for 1228 atom Marks decahedron (D=3.5 nm). For the 220 peak there is no effect and it is hardly noticeable for the 311 peak and higher angle peaks so one can conclude that for small decahedra (which have broad peaks) the additional MD related peak broadening is negligible. The most significant MD effect is thus change in the 111/200 profile.

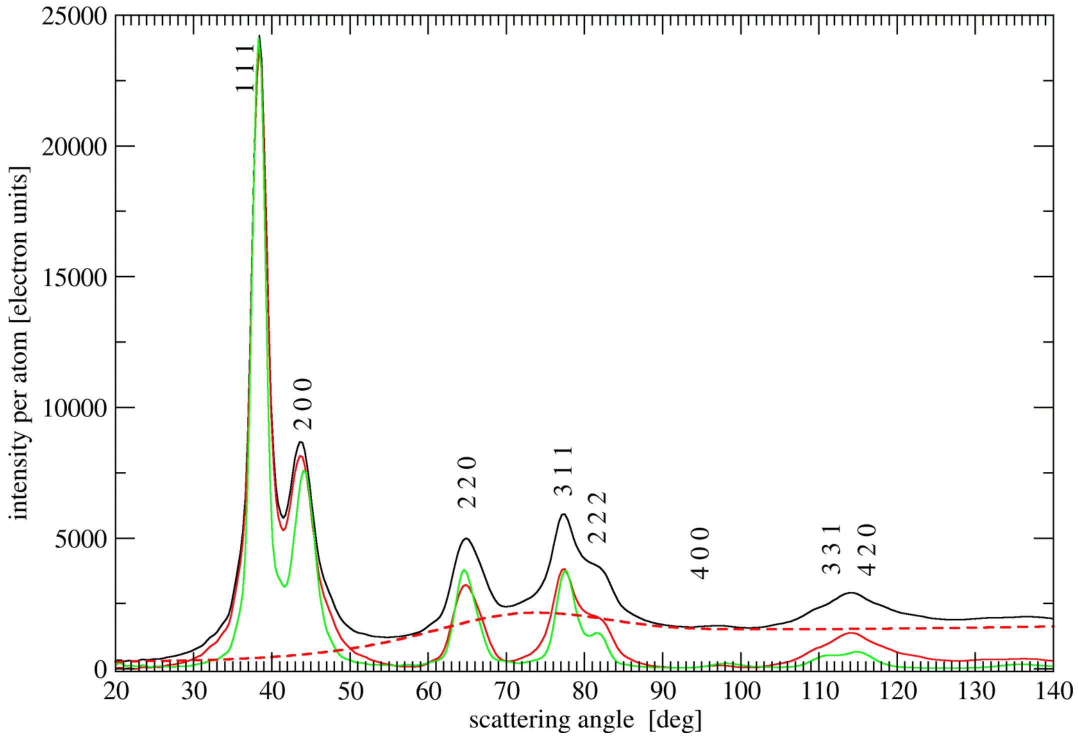

Fig.S18. Peak background (dotted red line) and pattern comparison for 7510 atom Marks decahedron: the energy relaxed model (pattern attenuated by $\exp(-3.5*\sin(\theta)^2)$)- green line, and MD thermalized at 800K (lattice constant scaled down by 1.03) -black line, and after background subtraction - red line.

MD simulation for the 1228 atom model already for temperature 750K shows surface melting so it is likely that the smallest model cluster patterns fitted to the experimental pattern correspond to gold particles with melted surface that in experiment occur at temperatures much smaller than that used in MD run.

The comparison to the attenuated patterns shows not perfect fit but the misfit is mostly related to the shape of 111/200 profile and to change in the arbitrary background.

The misfit can originate from another important structural feature of the samples. Au clusters are bonded to ceria support what is evident from the observed change of ceria properties for Au/CeO2 seen in XRD, TPR and TEM results. The Au-ceria epitaxy hampers the inner Au surface mobility and only the outer surface can be affected. Then the diffraction pattern will show on average less disorder than present on the gold outer surface. It is thus likely that the real Au contribution can be reflected better by mix of MD and relaxed decahedra models.

The overall high rate of decahedral Au peak attenuation with scattering angle seen in the experiments cannot be explained otherwise than by a significant disorder. To some extent it can have a static character but even then the surface Au atoms would have decreased coordination and bonding energy affecting surface mobility. A likelihood of mobile Au surface sheds new light on the possibility of surface Au atom (or dimer, trimers etc. [16]) or Au containing species diffusion to the support forming dynamic active sites on the ceria surface not only in a close perimeter of the Au/ceria boundary as suggested recently [17]. Such diffusion was indeed suggested by ab initio MD simulation [18].

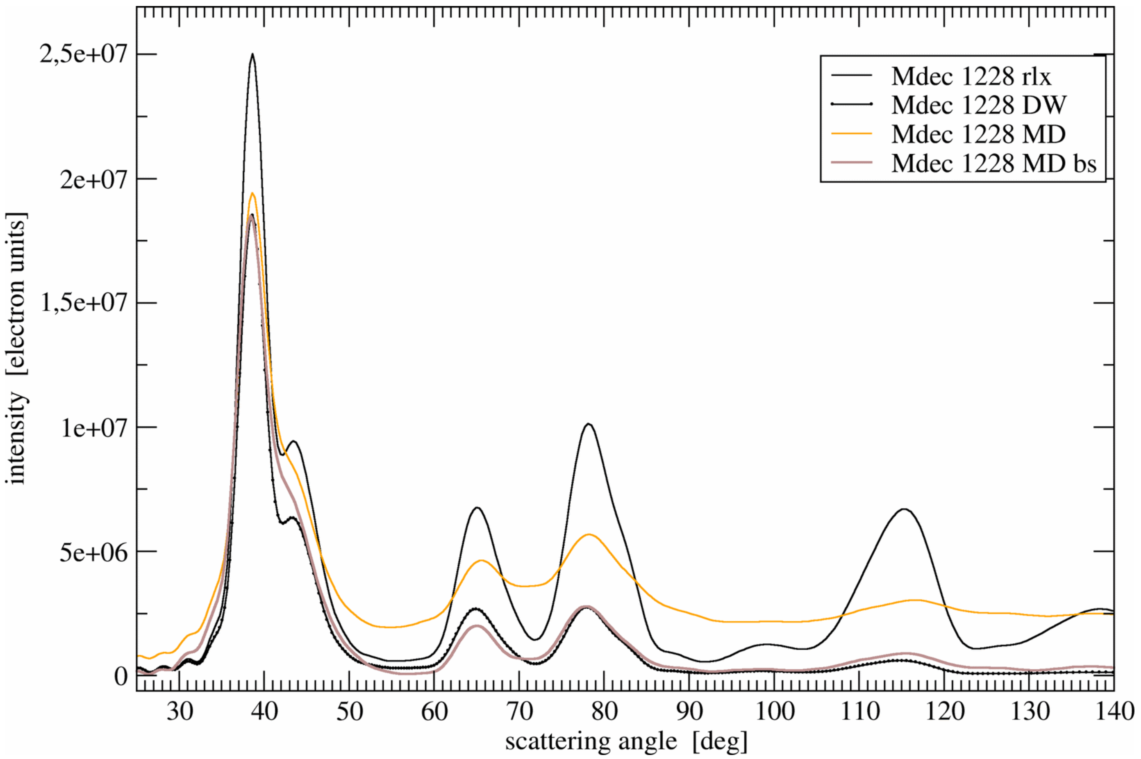

Fig.S19. Model pattern of MD thermalized at 750K 1228 atom Marks decahedron (lattice constant scaled down by 1.03)- orange (relaxed -black), background subtracted – brown, compared to DW attenuated (by exp(-3.5*sin(θ)^2)) pattern of the relaxed model- black dotted.

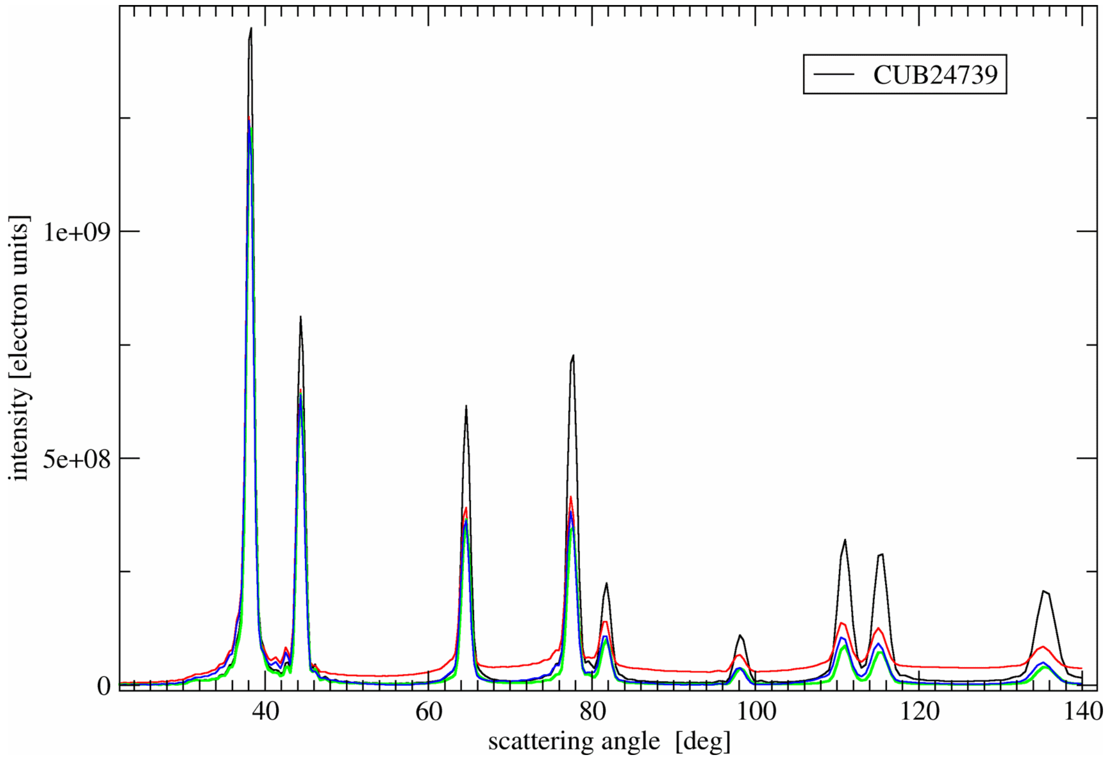

Fig.S20. Model pattern of MD thermalized at 800K 24739 atom cubooctahedron - red (relaxed - black) and background subtracted – blue, compared to DW attenuated (by exp(-3.5*sin(θ)^2)) pattern of the relaxed model- green.

Sample absorption correction.

In the applied measurement geometry a suitable absorption correction is given by Milberg [10]. A principal problem with its application is however a form of the sample being thin powder layer smeared over porous glass holder. As the catalyst fills small pores of the holder its effective thickness is hard to be determined. We applied alternative approach noting that the Milberg absorption correction in the form: $I_{obs}= I_{true}(1-\exp(-2\mu T/\sin(\theta)))$ is correlated with angular dependency of the Debye-Waller factor $I_{obs}=I_{true}*\exp(-2B*(\sin(\theta)/\lambda)^2)$ (μ is a linear absorption coefficient, T – sample thickness, θ – Bragg angle, λ – wavelength) as shown below.

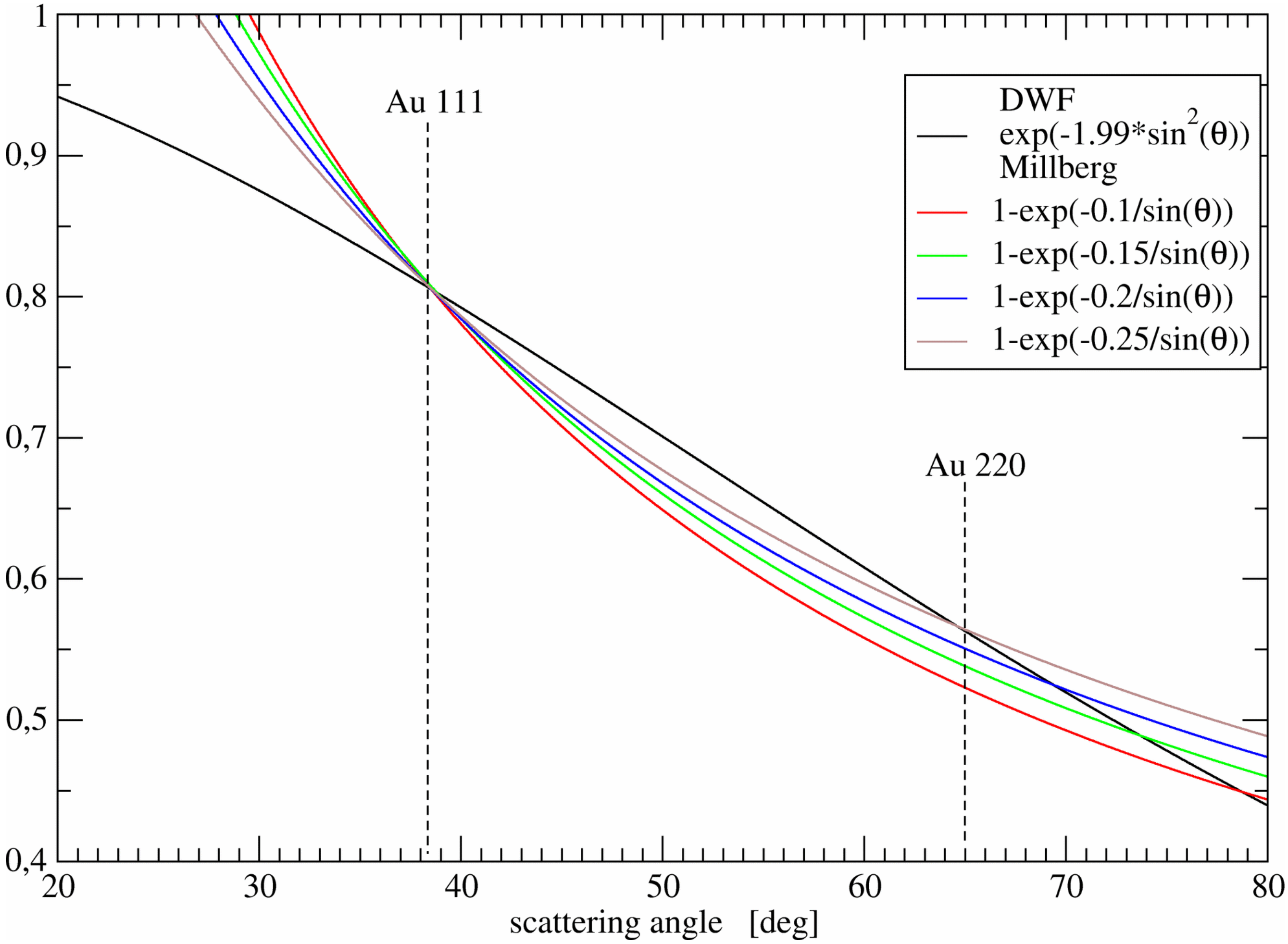

Fig.S21. Comparison of the Milberg correction factor with the Debye-Waller factor for a number of parameters.The positions of Au 111 and Au 220 are marked with vertical lines.

Variation of the Milberg correction in the experimental range is noticeable only for small values of 2μT. For 2μT>7 one can already assume approximation of infinite absorption and all observed Au peaks are not affected. Mass absorption coefficients (μ/ρ, where ρ – density) for gold and ceria have high values (>100 cm^2/g) at energies of Cu K line. Density of the pressed powder catalyst was astimated as ~5 g/cm^3. It results in μ>500 cm^{-1}. The absorption correction shows then visible variability only for the thickness T<0.007 cm = 70 μm ! As in experiments we used about 50 mg of the catalyst to be spread over the surface of ~1cm^2, the assumed density of ~5 g/cm^3 results in the catalyst layer thickness of 100 μm. It would suggest the sample absorption correction to be redundant. The question of uniform surface coverage may however play some role. To solve the question of sample absorption

correction applicability, we have compared DWF determined for a number of measurements performed on samples of varying mass. As the resulting DWF did not differ markedly, the Millberg absorption correction has been assumed to approximate well constant function corresponding to infinite thickness. This is not the case for the bare ceria sample for which ex situ measurement with thick sample layer reveals average square displacement $<u^2>=0.06$ Å^2 , in line with the result for the ceria in the catalyst. In this case the DWF results measured in situ on a thin sample layer spread over the porous glass disc are affected by the small thickness as described above.

Analysis of the gold phase.

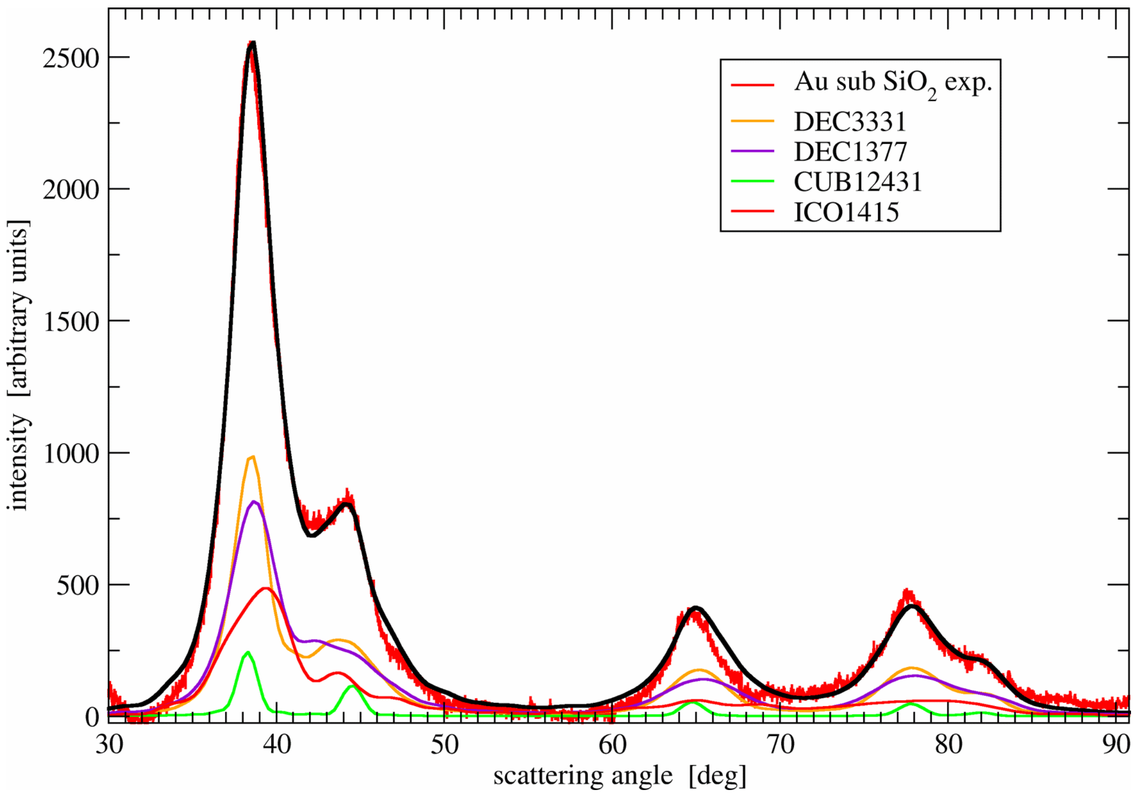

Fig.S22. Simple (consisting of few models) fit of the background subtracted Au/SiO_2 diffraction pattern. Fit – thick black line.

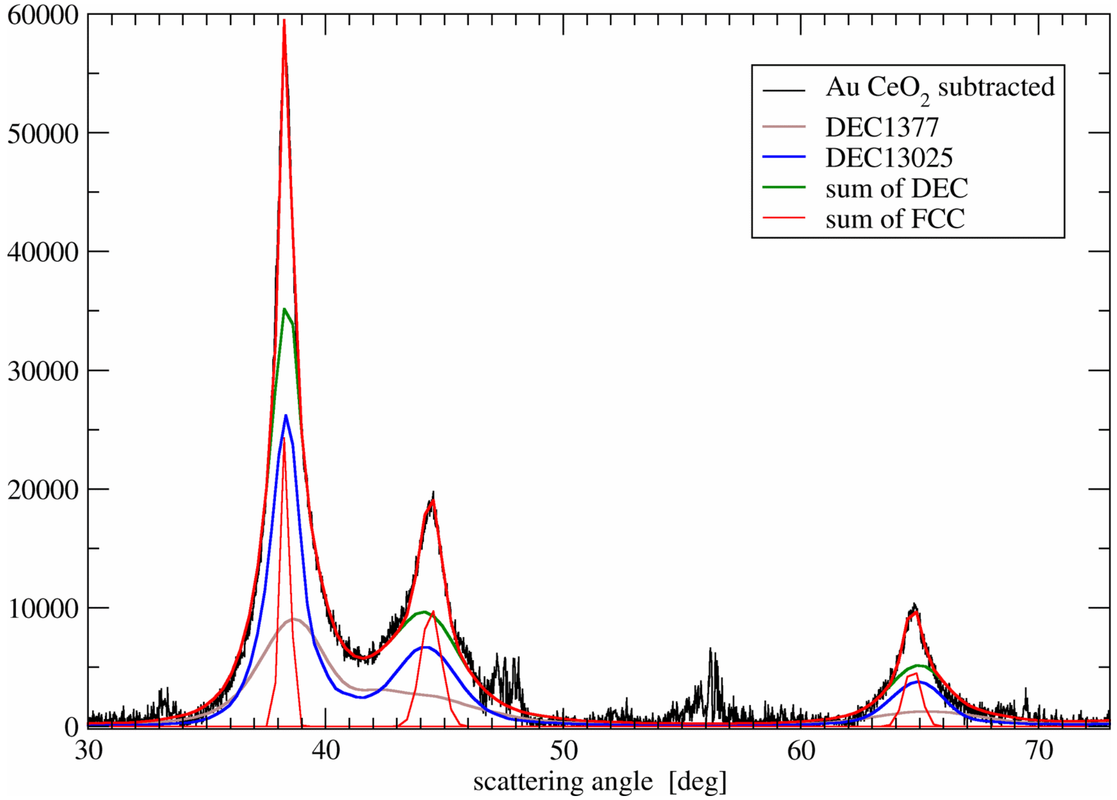

Fig.S23. Background (and support) subtracted XRD pattern of Au/CeO_2 compared to a model diffraction pattern calculated for several decahedra and cubooctahedra. Fit- thick red line.

The model fits presented in fig.S22 and S23 are barely a presentation of ability of several theoretical cluster models to approximate the Au patterns obtained from the experiment. They were not refined and the model clusters were selected intuitively. E.g. fit in fig.S22 required addition of small icosahedra to fill the right hand side asymmetry of principal maximum (referred to as 111) but fit in fig.S23 could be acceptably done without icosahedral clusters. It does not prove that Au/CeO_2 is deprived of icosahedra. Also contribution denoted as FCC in fig.S23 can likely correspond to large decahedra models (beyond size available to our simulation program). Its Gaussian peaks are at fcc gold positions but smaller height of 200 peak and its increased width strongly suggests decahedral structure.

The sizes of decahedra contributing to green line in fig.S23 are in broad range of 3-7 nm. Their relative contribution can be evaluated assuming proportionality of the peak area to the number of atoms (volume of Au crystal). It results in a number of observed 13025 atom decahedra (size about 7 nm) being 7.5 times less than the number of 1377 atom decahedra (3.3 nm). The estimated number of fcc crystals (of size 16 nm or more suggested from the peak width) is about 450 times less than that of 1377 atom decahedra in agreement with the size distribution histogram obtained from TEM. No wonder that in TEM histogram they can be spotted only occasionally for D>10nm, when the overall number of counted crystals is of the order of few hundreds. They however markedly shift a volume averaged crystal size as observed via XRD.

A relative contribution of the model clusters from fig.S22 can be estimated in similar way. Their distribution is dominated by 1377 atom decahedra (approx. 3.3 nm size). The number of 1415 atom icosahedra of comparable size (3.1 nm) is 1.8 times less, of 3331 atom decahedra (4.9 nm) is 2.8 times less, and of 12431 atom cubooctahedra (6.5 nm) is about 90 times less. This distribution broadly agrees with the size distribution observed from TEM.

To fit theoretical diffraction pattern to the observed differential patterns describing Au phase for both samples we had to attenuate peak intensities with angle using Debye-Waller factor (DWF). This necessity is shown in fig. S24. where it can be presented in the simplest form on the example of Au/SiO_2.

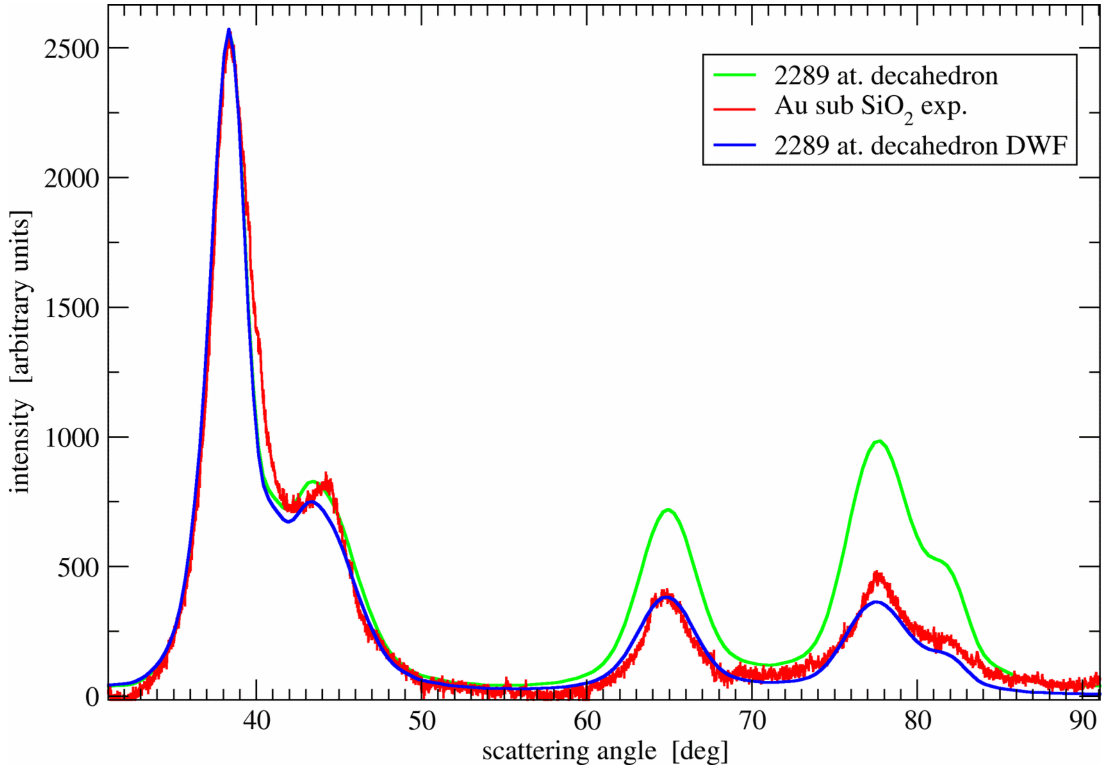

Fig.S24. Comparison of Au diffraction pattern extracted from the experimental Au/SiO_2 data with the pattern of a relaxed 2289 decahedron. Approximate fit requires DWF dumping of the latter by function $\exp(-3.5*\sin(\theta)^2)$ corresponding to a mean square displacement value of 0.18 $Å^2$.

The fit presented in fig.S24 shows directions for improvement. Adding some icosahedral Au will fill excess intensity on the asymmetric right hand side slope of 111 as shown on better fit of fig.S22. Using more realistic MD models of larger decahedra will provide 220 peak narrower than the other Au maxima as presented in fig.S18.
In most cases the used factor corresponded to a mean square displacement of Au atoms from their node positions in the model equal to about 0.18$Å^2$. This is quite large value that is possible to obtain for Au clusters of approx. 3-4 nm size using Molecular Dynamics simulations within Sutton-Chen scheme at 750K. MD reveals then high thermal disorder including melting of the gold cluster at the surfaces located far away of the 5-fold axis ([220] direction) – fig.S25 . As the degree of thermal disorder has to be correlated with melting phenomena we assume gold outer surface to be highly mobile with dynamically changing coordination of atoms.

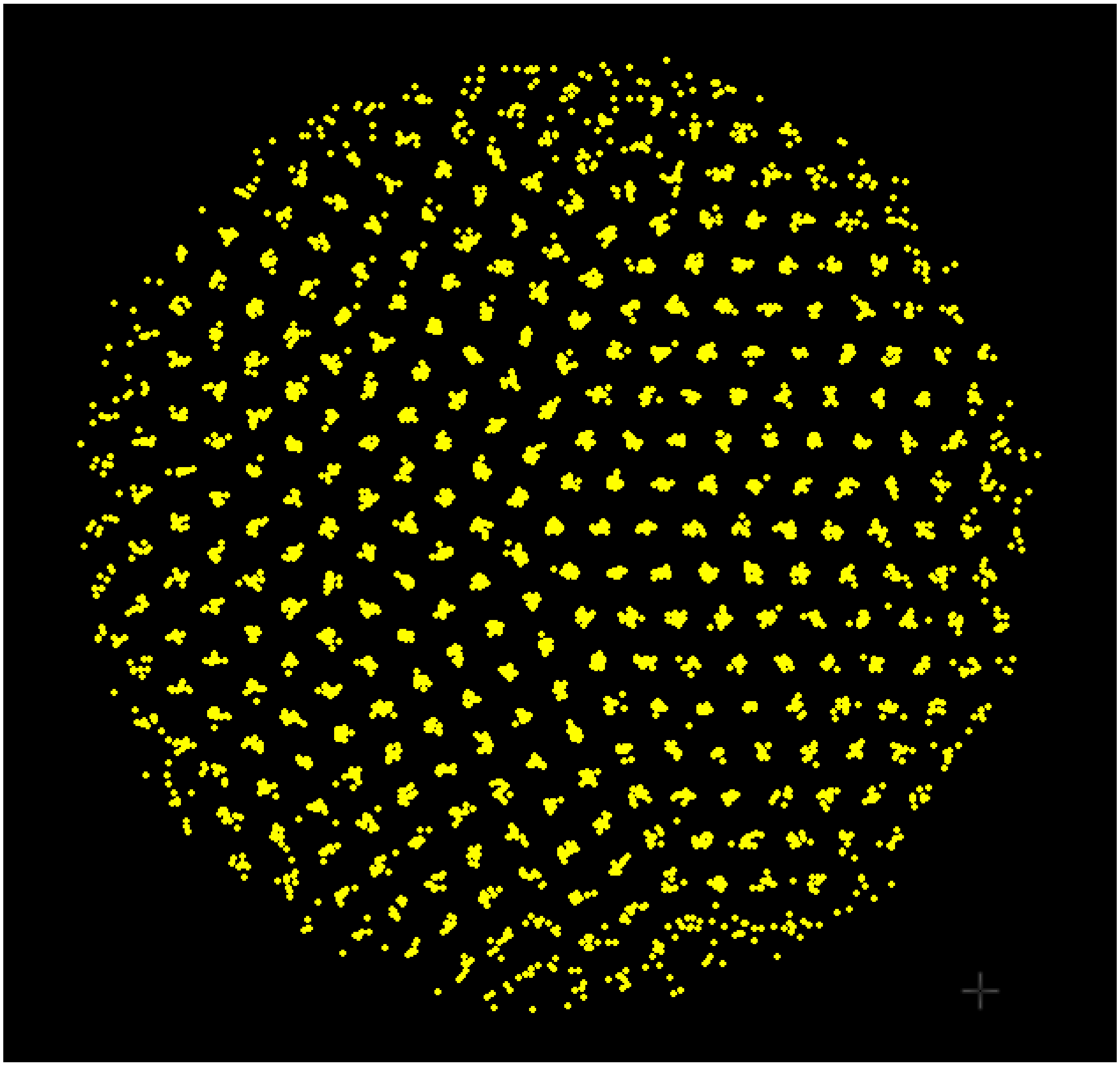

Fig.S25. Projection of 5862 atom Marks decahedron along [220] (5-fold axis). The model was MD equilibrated at 750K for 100 ps (1 fs step).

A very similar Debye-Waller factor value as for Au/CeO_2 applies to the diffraction pattern of sample Au/SiO_2 for which activity in CO oxidation was much lower as well as the detected rise of the temperature during the reaction. It points to the disorder being more general feature of Au catalysts morphology but insufficient to explain its activity.
As the dynamic phenomena change the diffraction profiles, below figure shows an attempt to fit DP of Au/C sample (exposed to He at the final step of the experiment at 423K) to the pattern calculated from MD equilibrated model of 5862 atom Marks decahedron at temperature 750K. The Au/C pattern bear close similarity to Au phase of Au/CeO_2 (fig.S11) with an advantage of displaying 311, 222 profiles non disturbed by the overlapping ceria peaks.

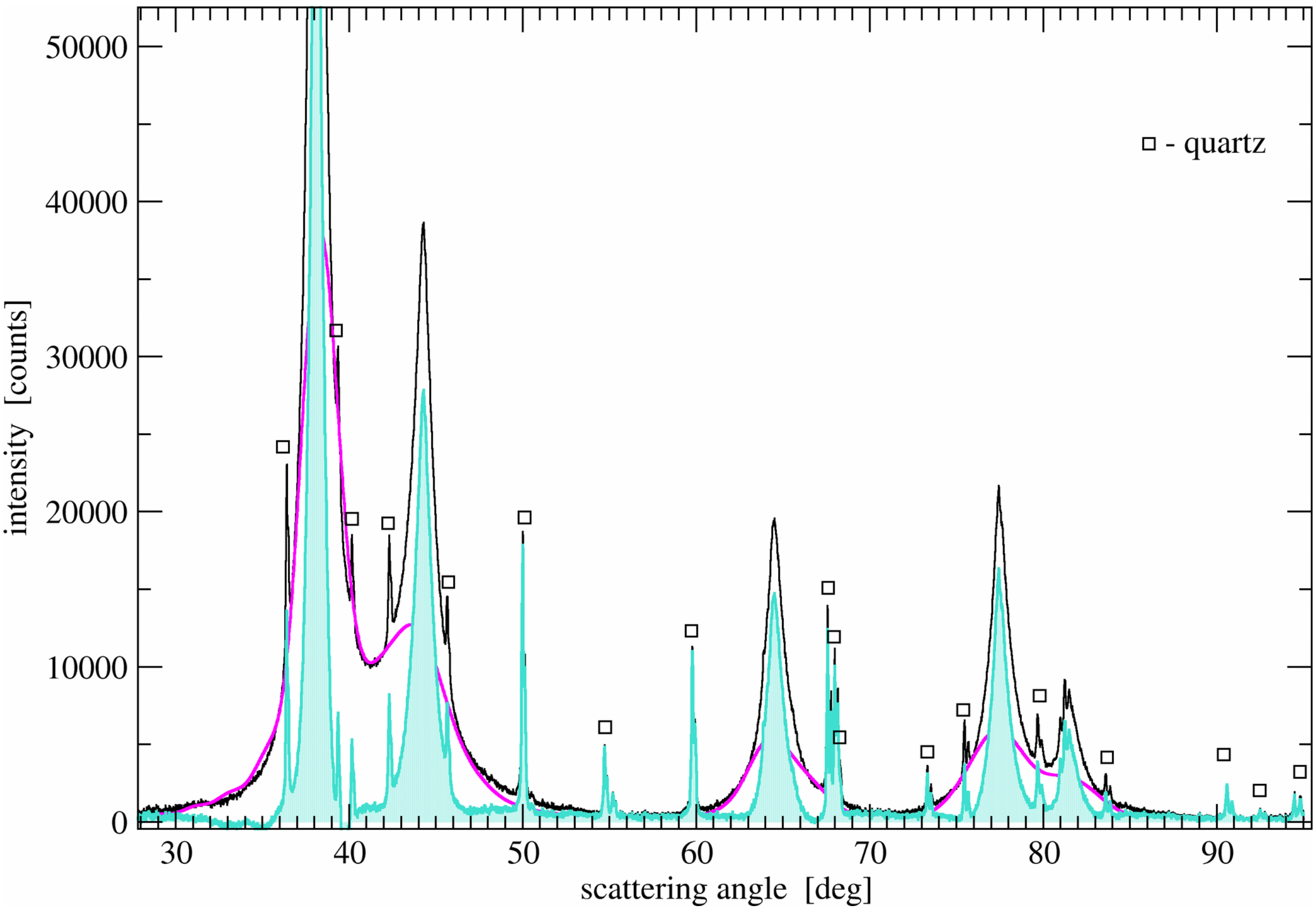

Fig. S26. Background subtracted diffraction pattern of Au/C (mixed with quartz marker – squares)-black line, fitted to the pattern of MD at 750K equilibrated Marks decahedron model (magenta line). The difference pattern (turquoise filled profiles) is a sum of quartz marker lines and a regular fcc Au phase peaks.

The residual fcc Au phase corresponds to larger Au crystals (size estimate from Scherrer eq. gives D>10nm depending on the peak). The peaks have integral intensities in a sequence 1000, 625, 297, 320,105 well resembling database room temperature data (JCPDS-ICDD 40784): 100, 52,32,36, 12 suggesting rather low value of DWF B-parameter. It suggests that the high Au disorder observed for small decahedra does not apply to the larger fcc crystals.

The disorder observed for small Au clusters resembling decahedra is not related to epitaxy since it is observed for various supports. Theoretical predictions of melting and premelting phenomena in metal nanocrystals is based usually on Landau theory applied to semi-infinite systems extended to finite models [19] or on atomistic modelling [20,21]. The former assumes spherical particles with surface free energy dependent on its size, the latter was attempted for more than 100 atoms typically using EAM- like n-body potentials. The former approach disregards surface structure and dependency of the surface energy on the face but still predicts bulk melting of 4nm Au cluster around 600K [22].

Analysis of the pattern evolution

Gold pattern evolution.

Important structural information concerning gold particles can be concluded from fig.S27. The background subtracted, absorption corrected Au 111 peak has been smoothed out (by running average over 7 points). Changes in the peak intensity can be interpreted in terms of a number of Au atoms at crystal lattice nodes and their displacement from the node.

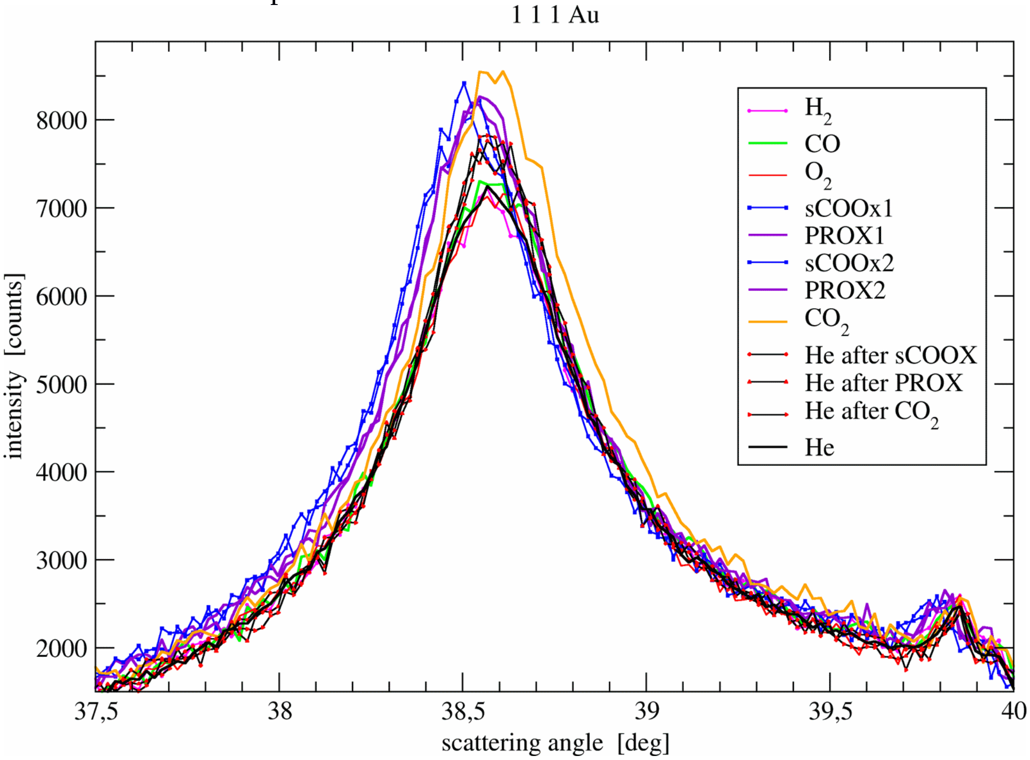

Fig.S27. Absorption corrected and background subtracted Au 111 peak profiles showing relative intensities in different gas atmospheres.

The initial sample in H_2 has the lowest intensity suggesting very mobile reduced surface of gold nanocrystals. Surprisingly the surface seems to be still mobile in CO slightly stabilizing in He in the reaction conditions (sCOOx).

For only three Au broad peaks monitored it is difficult to conclude on the observed changes being related to disorder or to the overall volume of the (quasi)crystal. As the rise in a peak height is often accompanied with fall of the width we have also checked overall integral intensities of 111 profile that broadly confirm differences observed on the peak height. The peak intensities were approximated by the profile integral between 35.5 and 42 deg. (111). To estimate possible change in DWF during the gas treatment history the 220 Au peak was also integrated between 63 and 67.5 deg. Table S4 lists integrated 111 intensities as well as a ratio of 220 and 111 intensities assumed to be indicative of DWF changes.

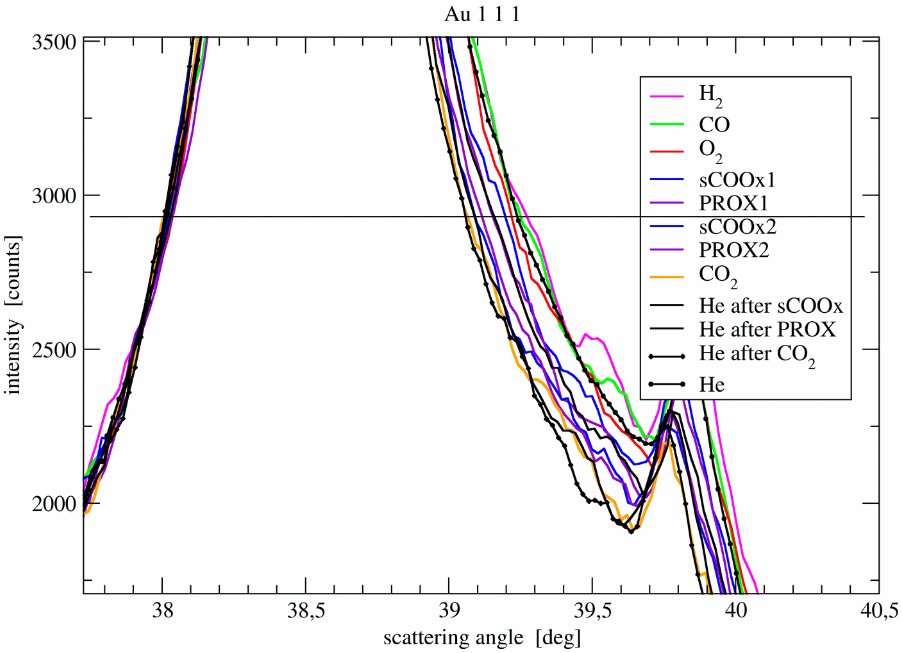

Fig.S28. Averaged DPs of 111 Au of Au/CeO_2 with the same scaled intensity and the left hand slope at the base of each peak moved to the same position to reveal differences in the peak width.

The observed peak narrowing during the experimental sequence is evidently due to slow gold sintering accelerated during steps of exothermic reaction.

Table S4
Au 111 integral peak intensity and a ratio of I(220)/I(111) at different gas atmospheres for Au/CeO_2 and Au/C catalysts

Gas atmosphere	111 Au integral int., Au/CeO_2 [counts*angular step]	I(220) / I(111) Au Au/CeO_2	111 Au integral int., Au/C [counts*angular step]	I(220) / I(111) Au Au/C
He	12047	0.232	186704	0.183
H_2	12357	0.230	185741	0.180
He	11991	0.238	185885	0.183
CO	12395	0.252	190831	0.192
He	11765	0.242	185551	0.187
O_2	11988	0.242	184336	0.189
He	12065	0.244	186556	0.189
sCOOx	13023	0.228	190574	0.188
He	12111	0.244	184966	0.187
PROX	12968	0.242	188110	0.187
SCOOx	12977	0.231	189533	0.192

PROX	12881	0.236	188012	0.190
He	11903	0.249	186360	0.188
CO_2	12953	0.251	207177	0.190
He	11815	0.244	185675	0.189

Analysis of lattice parameters- estimation of the coefficient of thermal expansion.

The measured lattice parameters have to be corrected on thermal expansion effect due to the catalyst temperature. The thermal expansion coefficients (CTE) were initially determined for SiO_2 quartz, Au and CeO_2 in He .In each case, the same in–situ PXRD-MS setup was used as for structure dynamics studies. Powder sample was pressed onto the porous glass plate which was placed inside the measurement chamber with flow of He (20 ml · min-1). Then the temperature was ramped according to the programmed sequence: 22°C (conditioned room temperature), 60°C, 120°C, 180°C, 230°C and 300°C.
For estimation of CTE for CeO_2 and Au, a 9.4% Au/CeO2 catalyst was used. Quartz was also mixed with the catalyst in order to provide an internal standard for better systematic errors correction (thanks to peaks' FWHM ~0.1°) and real specimen temperature.
Experimental patterns were processed using script under the Fityk program. Next, the crystal structures of quartz, Au and CeO2 were refined using least squares method. The lattice constants (or interplanar distances, dhkl) were plotted against the temperature. It was possible to obtain CTE from linearization of a dependence of interplanar distances d_{hkl} from temperature. CTE was determined as a slope of this dependence divided by the initial distance $d_{o\ hkl}$. The results are presented in Table S5.

Table S5

Material (hkl)	10^6 * CTE [1/K]	Δ CTE [%]
SiO_2 quartz (100)	14.23	2.5
SiO_2 quartz (001)	7.97	3.7
Au NP	13.51	2.0
CeO_2	8.03	1.6

The lattice parameters measured in reaction conditions had to be corrected for the determined temperature using the experimental CTE. The corrections are of slightly worse accuracy than that of the experimental ceria lattice parameters. This is why no corrections were applied to results in atmospheres where was no running reaction.

Measured lattice parameter pointing to structure of the crystal interior.

For small bi-elemental crystals when the number of surface atoms of one kind is comparable to the number of those remaining in the bulk (of second kind), the lattice parameter observed via diffraction

can evolve e.g. during surface segregation process without changing of the overall elemental contents. It was shown experimentally [23].

A simple theoretical experiment was performed to show the contribution of the surface and the core of the nanocrystal to the diffraction pattern profile. The model nanoparticle was the ideal cuboctahedron made of 1415 atoms of palladium out of which the outer layers containing the total of 708 atoms were considered as the surface single-layer of the nanocrystal (also called “its shell”) and the remaining 707 constituted the core. In both parts the Pd atoms arrangement agreed with the FCC structure.

Two models were built. In the first one, the core was based on an FCC unit cell with a_{core} = 3.90 Å, while the surface part had the lattice parameter a_{shell} = 3.89 Å. The second model had inversed core-shell properties. Both models were constructed in the Cluster program [13]. The interatomic interactions in the core and the shell (surface) followed the Sutton-Chen n-body potential approach [12]. The only difference in potentials describing each part of the nanocrystals was the elementary cell constant a, as mentioned earlier. The interaction potential between Pd_{core} – Pd_{shell} corresponded to the reference palladium crystal structure (a = 3.89 Å). The crystal structures of both models were relaxed, i.e. energetically minimised.

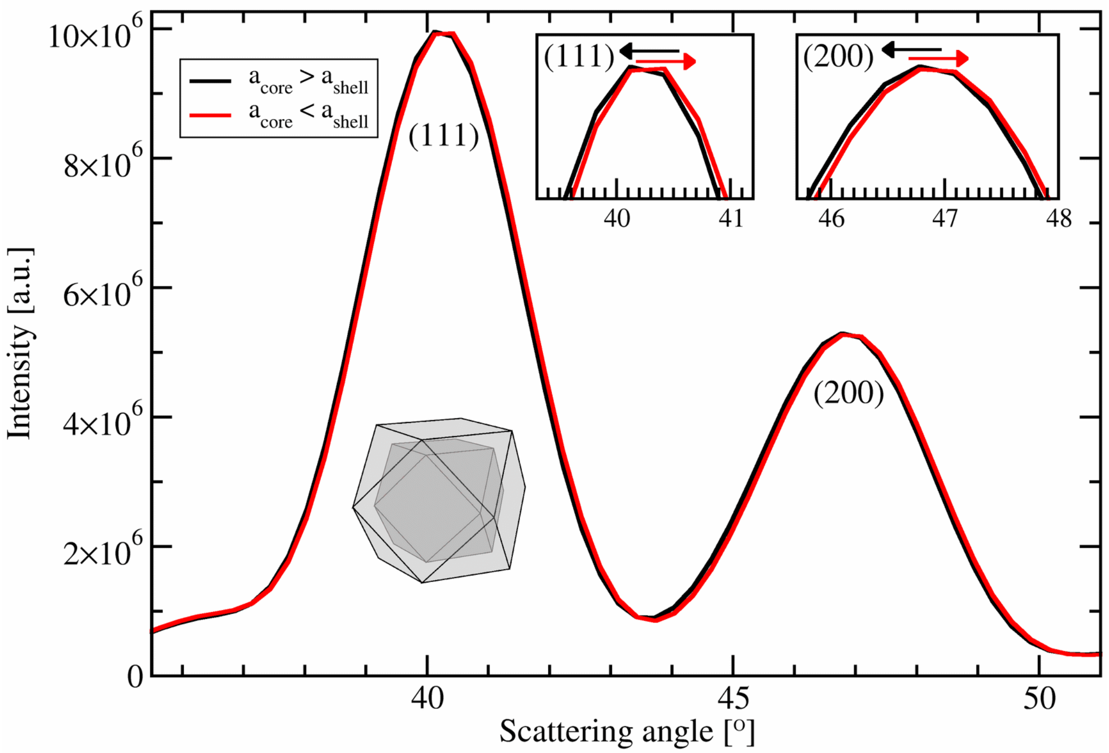

Fig.S29. The diffraction patterns of the relaxed (energetically minimised) cuboctahedral core–shell nanocrystals containing 1415 Pd atoms simulated in the Cluster program [13]. The black curve represents nanoparticles, in which the core was based on 707 Pd atoms ordered in the FCC structure with unit cell parameter a = 3.90 Å, while the shell (corresponding to the surface layers of the particles) was made of 708 FCC-structured Pd atoms with a = 3.89 Å. The red curve represents nanocrystals with inverted core-shell properties.

The calculated diffraction patterns (Fig. S29) showed that the diffraction peaks were shifted to lower or higher scattering angles following the lattice parameter of the core. This example clearly showed that the bulk part of the crystals influences more the diffracted intensity profile than the surface, although the numbers of scattering objects in the core and the shell were the same. Hence, the surface phenomena must involve deeper parts of the nanocrystal structure to make the effect visible in the

experimental diffraction pattern. Similarly, the redistribution of the defects, initially formed on the surface, towards the inner parts of the particle will take bigger effect on the DP than accumulation of these defects near the surface.

Temperature Programmed Desorption (TPD) and Reduction (TPR).

The TPD/TPR experiments used glass apparatus: 1. equipped with TCD using H2 (10%) in Ar carrier gas; 2. using He carrier gas or 20% H_2 in He, and Mass Spectrometer (MA200, Dycor-Ametec, Pittsburgh, USA) detection.

Measurements were done for the Au/CeO_2 sample and for the pure support CeO_2. As we established during the in-operando PXRD-MS studies, hydrogen does not adsorb on the clean CeO_2 surface at least up to temperature 150°C. The situation changes after gold nanocrystals are deposited on ceria surface. Such adsorption of H_2 on Au/CeO_2 is reversible what was confirmed by the change of ALP of CeO_2 back to its original value immediately after the gas atmosphere was exchanged to pure helium. Indeed, the same observations were made during TPR-TCD studies.

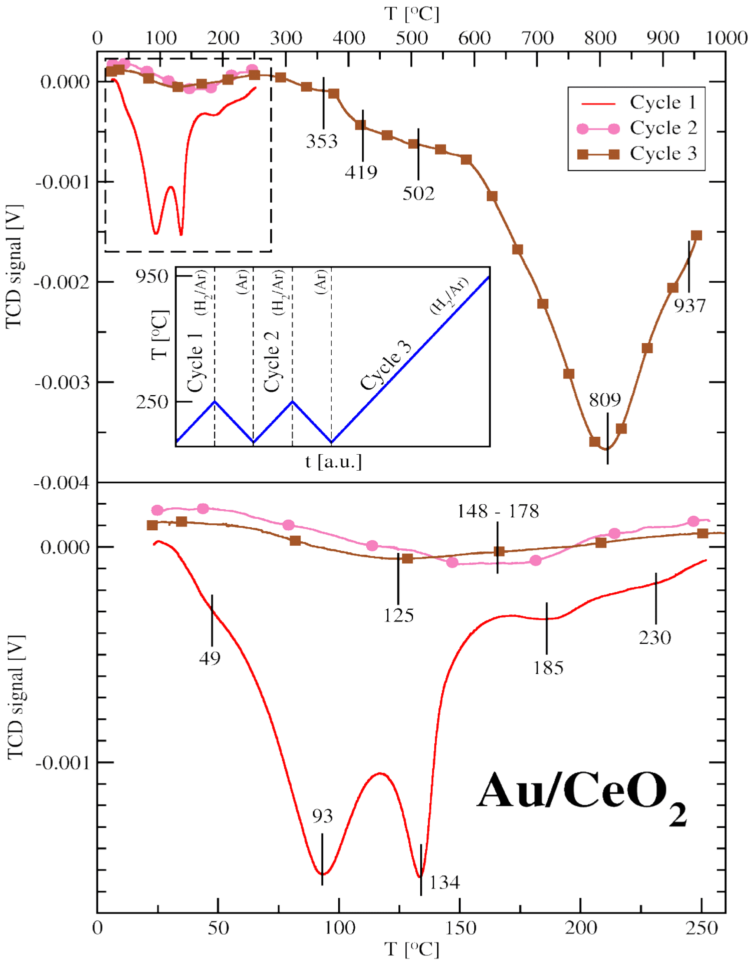

Fig.S30. 3 TPR-TCD cyclic profiles of the Au/CeO_2 catalyst. Cycle 1 and 2 were collected until the temperature reached 250°C and the 3rd cycle was collected up to 950°C. The profiles were acquired under 10% H_2/Ar atmosphere and the sample was every time cooled down to room temperature under pure Ar before the cycle was restarted.

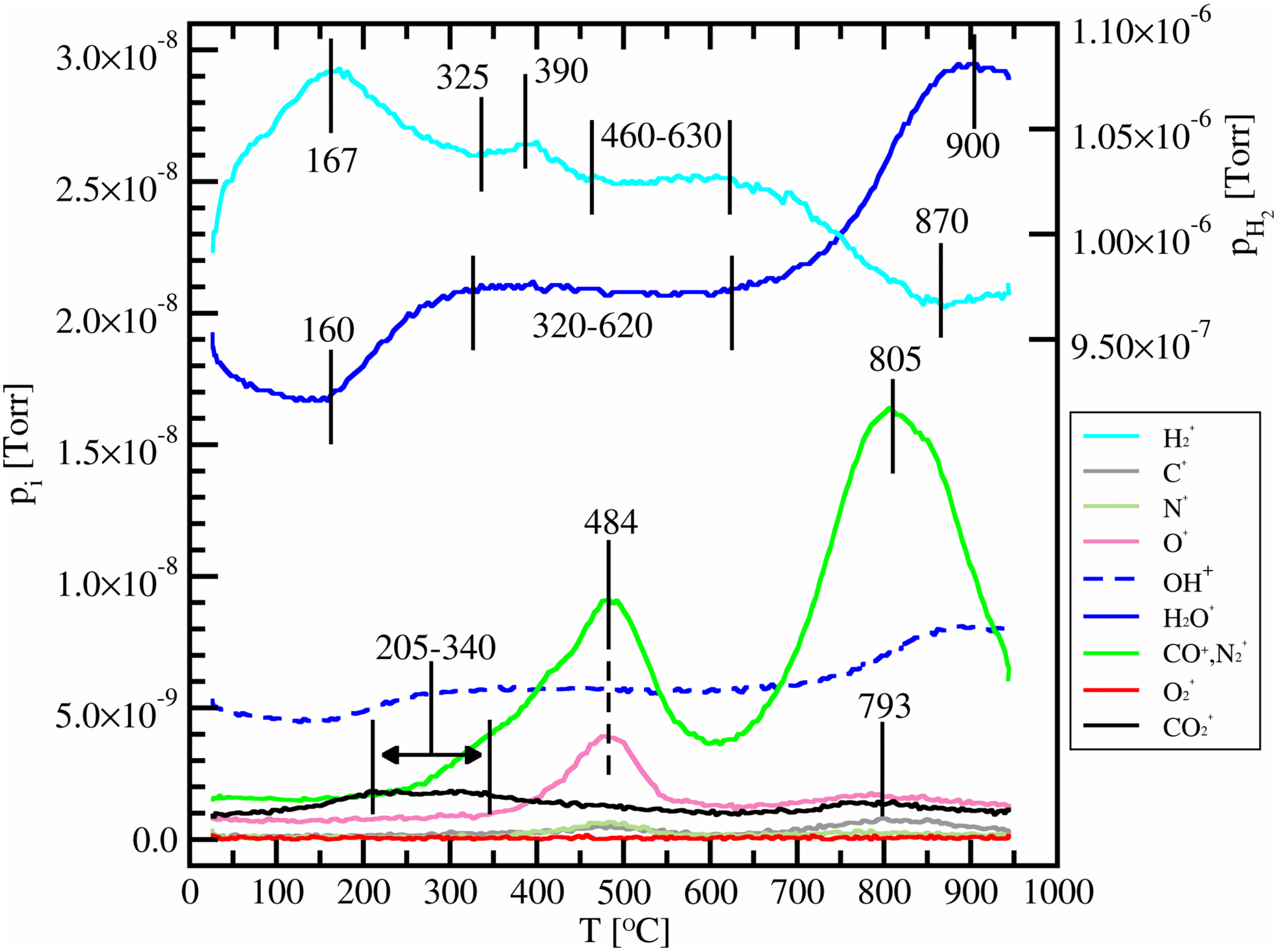

Fig.S31. The mass spectrum TPR profile of the air-exposed Au/CeO_2. The carrier gas was 20% H_2/He.

The Au/CeO2 TPR-TCD profiles (Fig.S30 cycles 2 and 3) acquired cyclically one after another, revealed each time peaks in the 125 - 178°C range due to hydrogen adsorption. This is not the case for pure CeO_2 (Fig.S32 cycles 2 and 4) where no interaction with hydrogen was observed in this moderately low temperature regime.

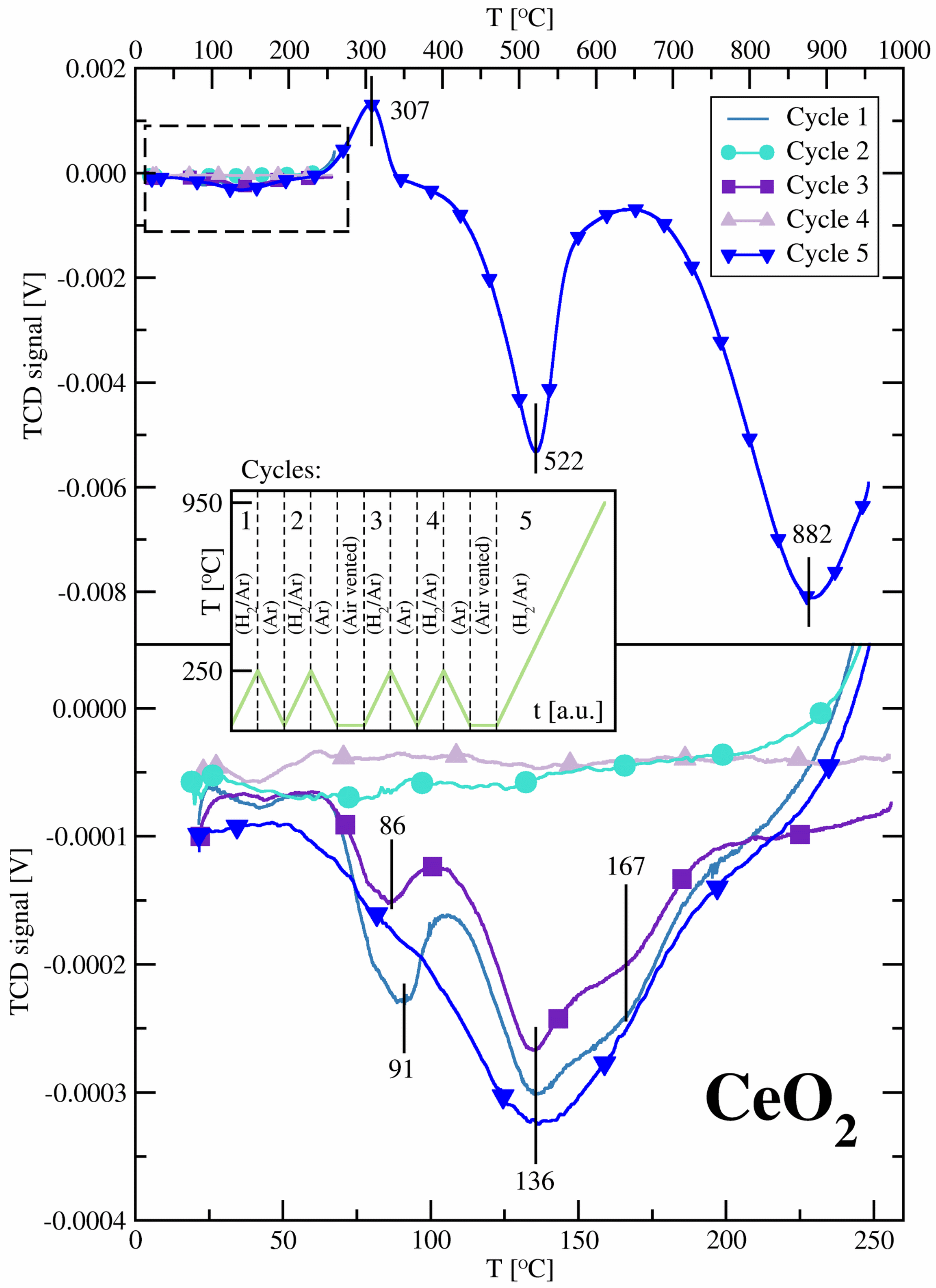

Fig.S32. 4 TPR-TCD cyclic profiles of CeO_2 registered with carrier gas 10% H_2/Ar up to 250°C showing how the adsorbates from air influence the TPR studies. The sample was vented with air for 1 h between cycles 2 and 3 and before the final cycle 5 covering temperatures up to 950°C.

In order to preserve the nanocrystalline form of gold on the surface of ceria and to keep experimental conditions the same for all materials, none of the samples was preheated under inert atmosphere before the TPR profiles were collected. Such procedure imposed certain phenomena to be observed during each first cycle of the TPR experiments before the temperature exceeded approx. 250°C.
Hence, in the case of pure CeO_2 the first cycle of TPR always involved desorption of CO_2 at around 130 - 150°C and it was not visible any more unless the sample was exposed to air again (see cycle 3 in Fig.S32). This fact was further confirmed in the TPR-MS studies (Fig.S33) by the corresponding rise of the m/z=44 signal.

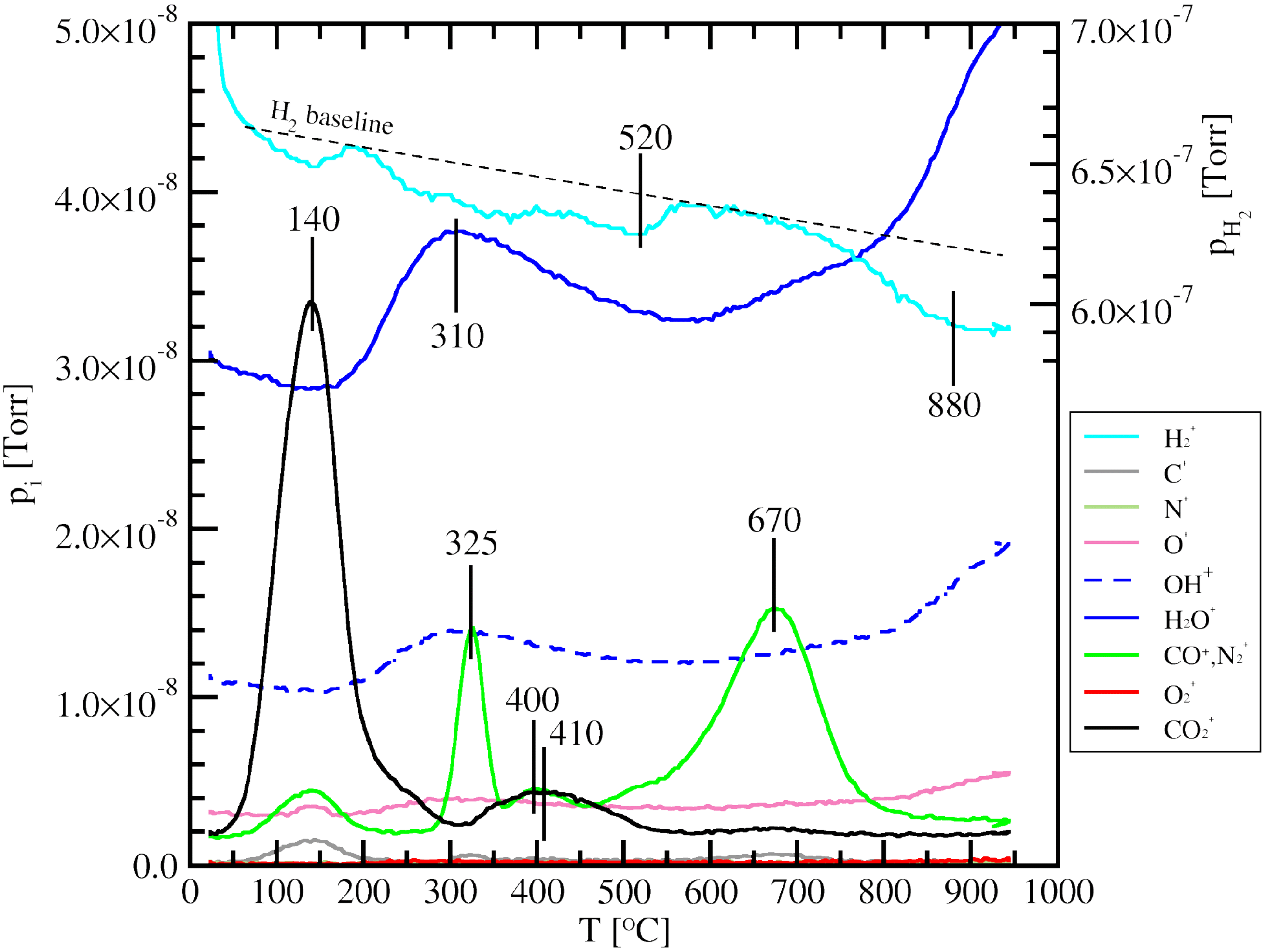

Fig.S33. The mass spectrum acquired during TPR run showing the desorption products of the air-exposed CeO_2. The carrier gas was 20% H_2/He.

Additionally, at approx. 95°C another signal on the TPR-TCD profile can be spotted. It can be attributed to only slight consumption of hydrogen from the stream as it may be suggested from the MS data. Despite the lack of hydrogen adsorption on clean CeO_2 at such low temperatures, the surface containing residues of air is presumably capable to exchange them for small amount of hydrogen. This weakly adsorbed H_2 can be easily flushed out by inert gas. e.g. He.

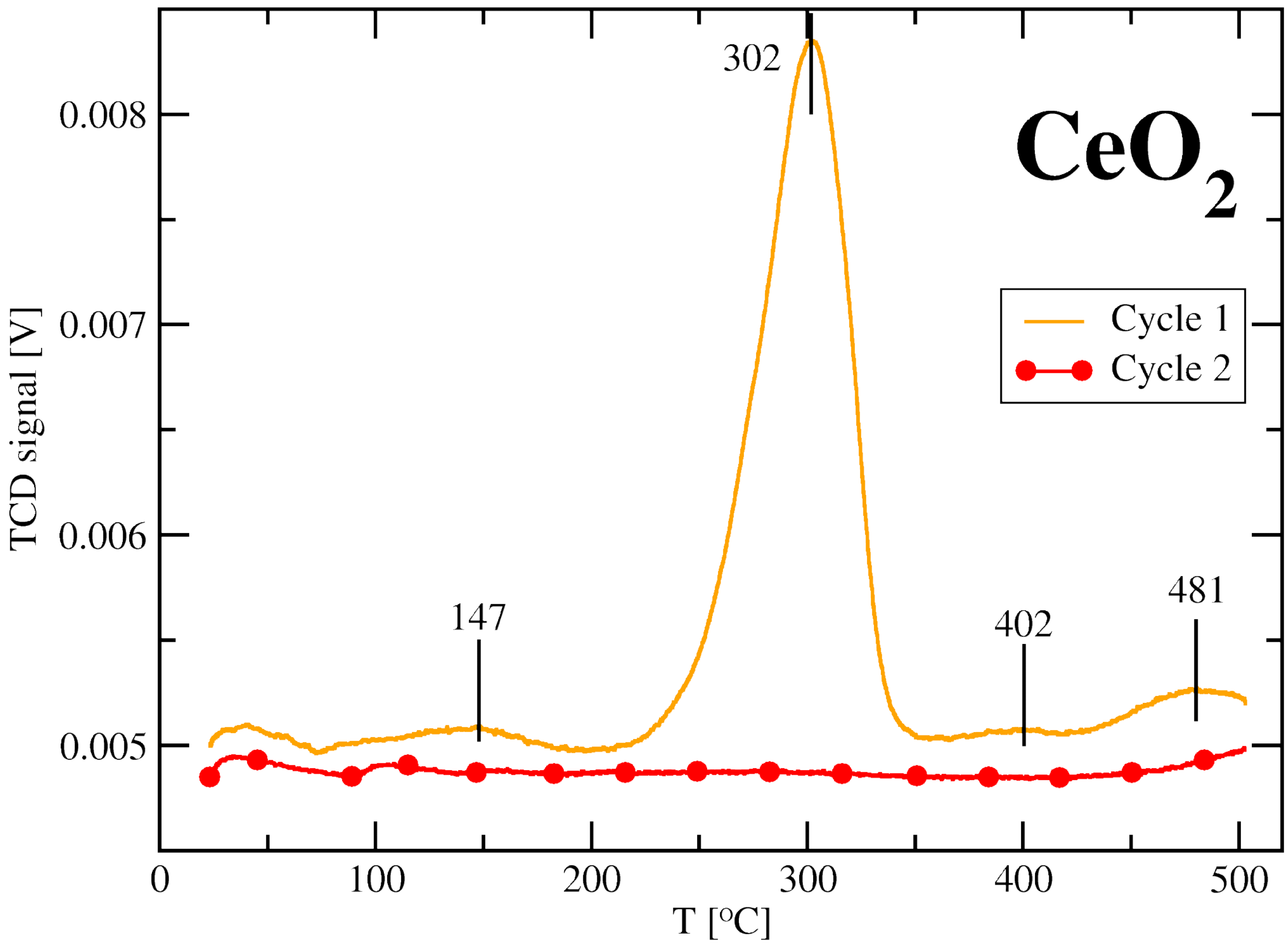

Fig.S34. The TPD-TCD profile of pure CeO_2 in Ar.

The high temperature TPR studies revealed that there is also CO_2 strongly bound to the pure CeO_2 surface and it desorbs only above 300°C after decomposition to CO (see Fig.S33 and Fig.S34). Following the TPD-TCD and TPD-MS results (Fig.S34 and Fig.S35), this is thermally induced decomposition which proceeds above approx. 500°C only under H_2-containing atmosphere - when phase reduction of CeO_2 starting at its surface begins. Otherwise, the thermal energy provided to the system above 500°C causes the remaining CO_2 to desorb molecularly. Formation of CO leaves oxygen at the CeO_2 surface and, thus, makes the reduction peak observed at 520°C in the TPR-TCD studies so intense [24] (Fig.S32, upper curve).

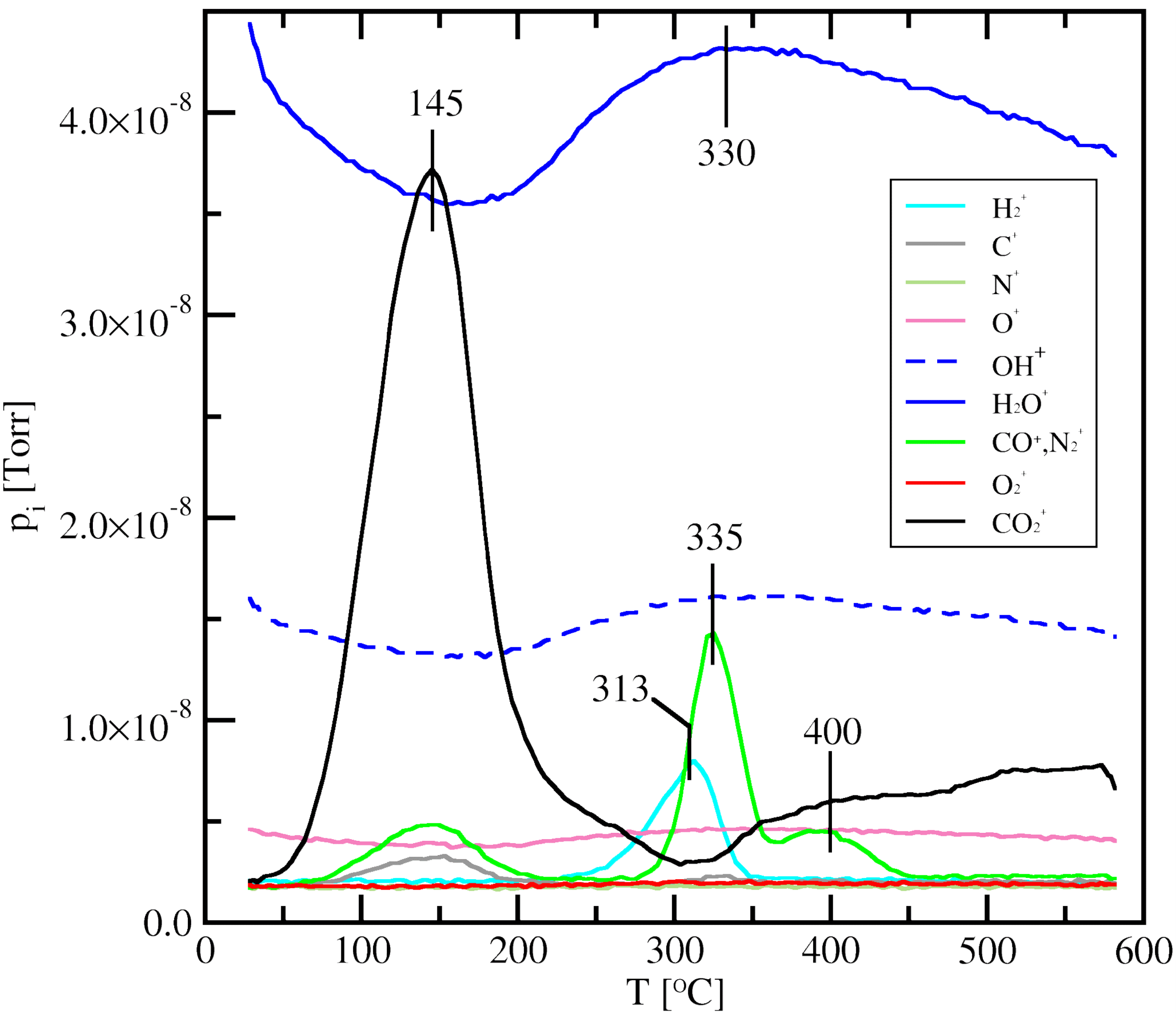

Fig.S35. The mass spectrum desorption profile of the air-exposed CeO_2 in He.

Further TPR-TCD peaks correspond to both: CO release diluting the carrier gas stream and consumption of H_2 due to phase reduction of CeO_2 which starts at the surface - the peak at approx. 520°C - and then propagates to the bulk - the peak at approx. 880°C) (Fig.S32). After the measurement, the initially pale yellow CeO_2 turns into blue, but it is then extremely sensitive to air and oxidizes rapidly after venting, turning back to yellowish colour.

On the contrary to pure CeO_2, the interaction of H_2 with Au/CeO_2 catalyst is markedly different. Au facilitates adsorption of H_2 (supposedly rather chemisorption) on CeO_2 at low temperatures (below 300°C) that is essentially not observed for pure CeO_2. H_2 bound to the catalyst seems to undergo two processes: 1) adsorption on CeO_2 surface, that is weak and reversible (compare cycle 2 and the beginning of cycle 3 with cycle 1 in TPR-TCD experiment in Fig.S32);
2) chemisorption followed by subsequent reactions with species previously adsorbed on the surface of CeO_2 (namely air components, mainly CO_2).

Different H_xCO_y species have been already identified to exist on the surface of CeO_2 under favourable conditions (e.g.[24,25,26]).
Fixation of CO_2 in the form of its derivatives with hydrogen on the surface of Au/CeO_2 catalyst might be the reason why desorption of CO_2 (eventually released as CO) is markedly shifted to higher temperatures. The m/z=28 amu peaks' tops are recorded at around 484°C and 805°C (Fig.S31) while the pure CeO_2 released CO in two steps as evidenced by the peaks at 325°C and 670°C on the TPR-MS spectrum (Fig.S33).
We believe that, despite the expected complex interactions between H_2, CO_2 and the Au/CeO_2 catalyst surface, some small amount of CO_2 can be still freely released following the observed increase of the m/z=44 amu MS signal between 205 and 340°C (Fig.S31).

Similarly to the case of the pure CeO_2, the TPR-TCD profile of the catalyst above 300°C is a sum of two effects: CO release of lesser influence on the TPR-TCD profile and the more dominating reduction of CeO_2 starting at the surface and propagating towards the particles core, what is evidenced by rising H_2 consumption in TPR-MS. In the case of the catalyst, the TPR-TCD peaks' tops at high temperatures are shifted to lower values (e.g. from 880°C to 810°C) or are largely broader - e.g. the peak at 520°C for pure CeO_2 turns into wide hump starting from approx. 300°C for Au/CeO_2 and smoothly connects with the slope of the next peak.

X-ray Photoelectron Spectroscopy.

The XPS data (Fig. S36) were collected using PHI 5000 VersaProbe (ULVAC-PHI, Japan) spectrometer using monochromatic Al K_α −radiation (1486.6 eV). The element's high-resolution (HR) spectra were measured with the energy step 0.1 eV at the pass energy 23.5 eV. The X-ray source operated with 100 µm spot size, 25W and 15 kV. The samples were pressed into thin wafers, loaded and degassed in a load lock chamber. The data were analyzed by CasaXPS software with sensitivity factors supplied by PHI. HR spectra deconvolution included Shirley background subtraction with Gaussian-Lorentzian or LF(90.76, 1.5, 55, 400) peak shape. The peak position corrections due to charging were done by adjusting Ce $3d_{3/2}$ peak (usually described as the u''' peak) to a position of 917.00 eV [58–60] . The analyzis of Au/CeO_2 catalyst data suggests Au crystals in metallic state with peak Au $4f_{7/2}$ at 84.03 eV [61] .
The Ce 3d spectra show characteristic three doublet peaks of Ce^{+4} located at 882.62 eV ($3d_{5/2}$ − v) and 901.07 ($3d_{3/2}$ − u), 889.02 eV ($3d_{5/2}$ − v'') and 907.47 eV ($3d_{3/2}$ − u''), 898.55 eV ($3d_{5/2}$ − v''') and 917.00 eV ($3d_{3/2}$ − u'''). 2 doublets characteristic of Ce^{+3} are located at 881.28 eV ($3d_{5/2}$ − v^0) and 899.74 eV ($3d_{3/2}$ - u^0), 884.12 eV ($3d_{5/2}$ − v') and 902.57 eV ($3d_{3/2}$ − u') [58–60,62] .The contribution of Ce^{3+} was estimated from a ratio of peak areas: (v^0 + v' + u^0 + u')/(v^0 + v'+ v + v'' + v''' + u^0 + u' + u + u'' + u'''). The calculated contribution of Ce^{+3} to Ce is 21.1 %.

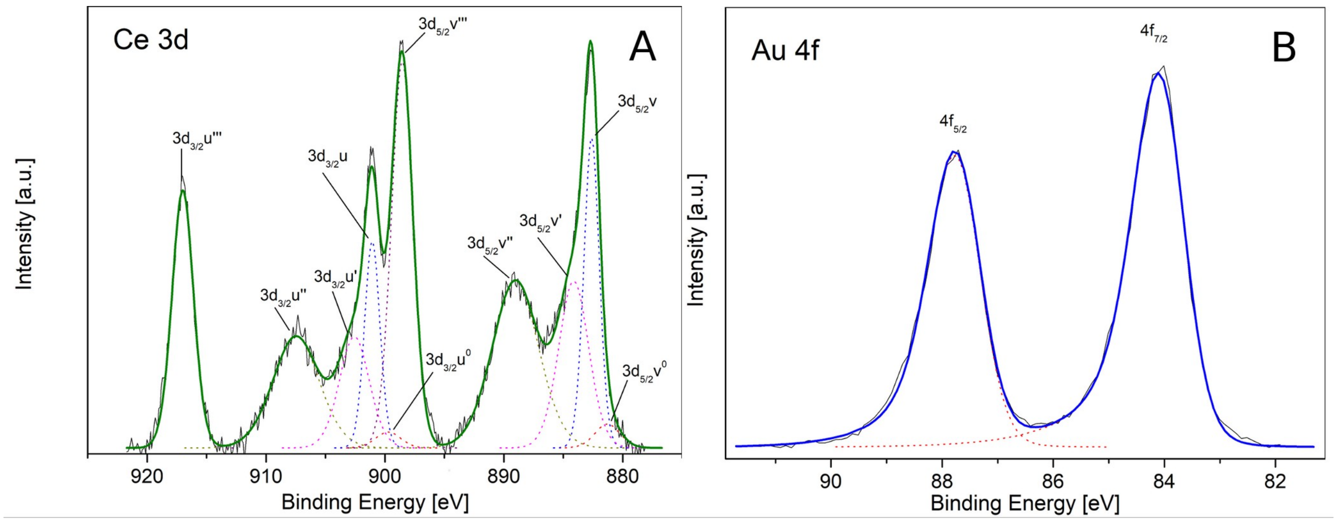

Fig.S36. XPS Ce 3d (A) and Au 4f (B) spectra for the Au/CeO_2 catalyst.

Gas absorption within the x-ray camera- chemical reactor.

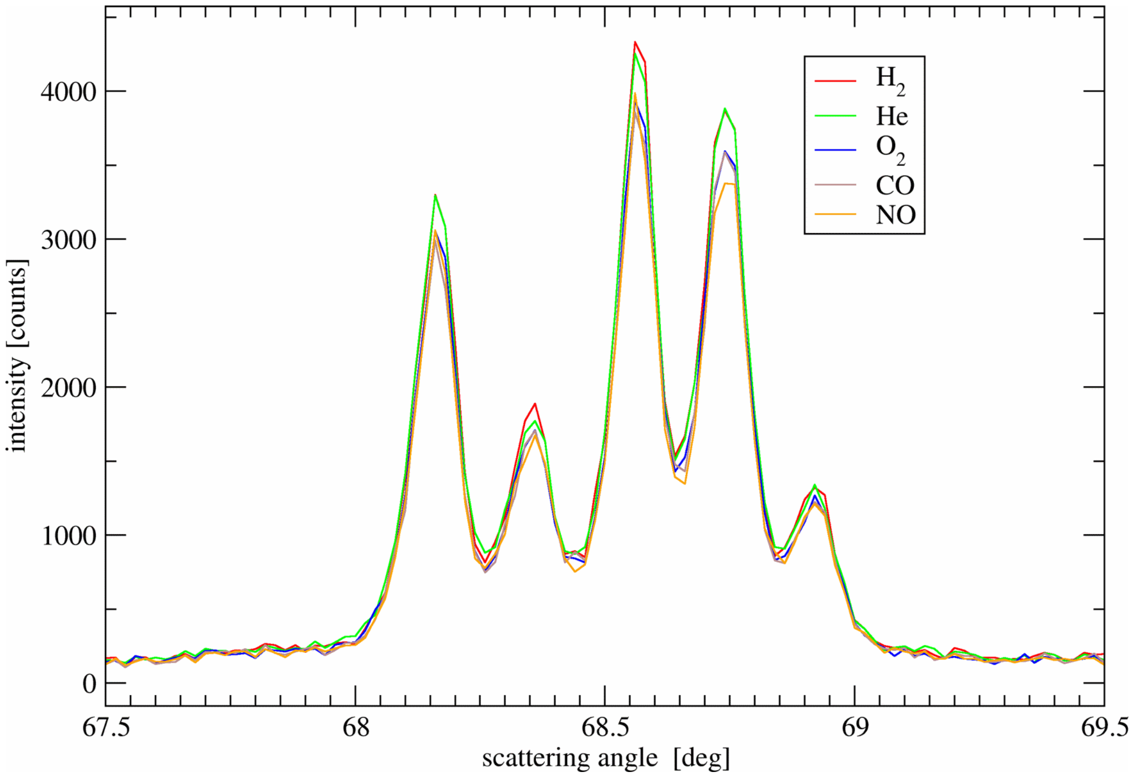

Fig.S37. Illustration of the gas absorption within the in-situ reactor for a sample of alpha quartz – diffraction standard supplied by Bruker. For clarity the pattern restricted to quartz ‘fingers’.

To increase precision of the correction it was applied to the total measured intensity within the range 15-100 deg. equal about 39100 counts. The differences between the corrected intensities scatter within the range of 85 counts – less than statistic error of 198 counts.

Precision and repeatability of the peak position.

Fig. S38. Repeatable measurements of the NIST LaB_6 standard 210 peak position. The data collected after 1 hour tube heat up period for more than 1 day. The position standard deviation of σ= $9*10^{-5}$ degree corresponds to the lattice constant error of $7*10^{-7}$ nm. All data fall within the ±3*σ range.

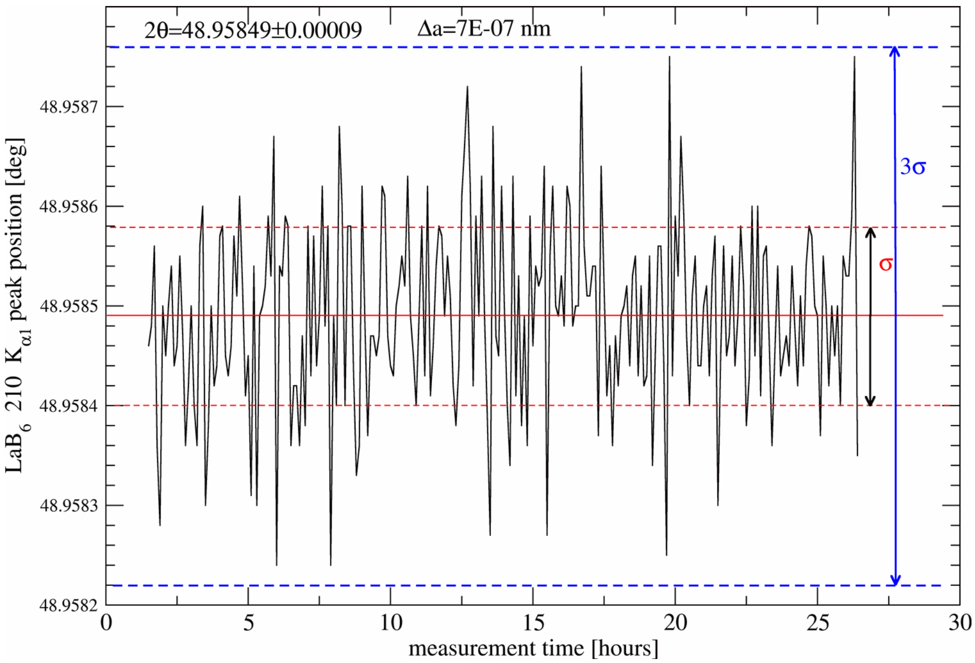

Mass spectrometry monitoring of the blind experiments on the sample holder (and porous glass) alone.

Fig. S39. Mass spectrometry (MS) analysis of composition of the gas stream leaving the PXRD chamber with the sintered glass disk alone. The actual temperature of the steel holder (bottom) and MS signals corresponding to relevant cations are shown. Data are plotted against time with unit of approx. 44 min. The sCOOX phase corresponds to 7 ml/min of O_2 and 14 ml/min of CO, PROX- 10 ml/min of H_2, 5 ml/min of CO and 5 ml/min of O_2. Evident is non-linear response of the Faraday cup detector.

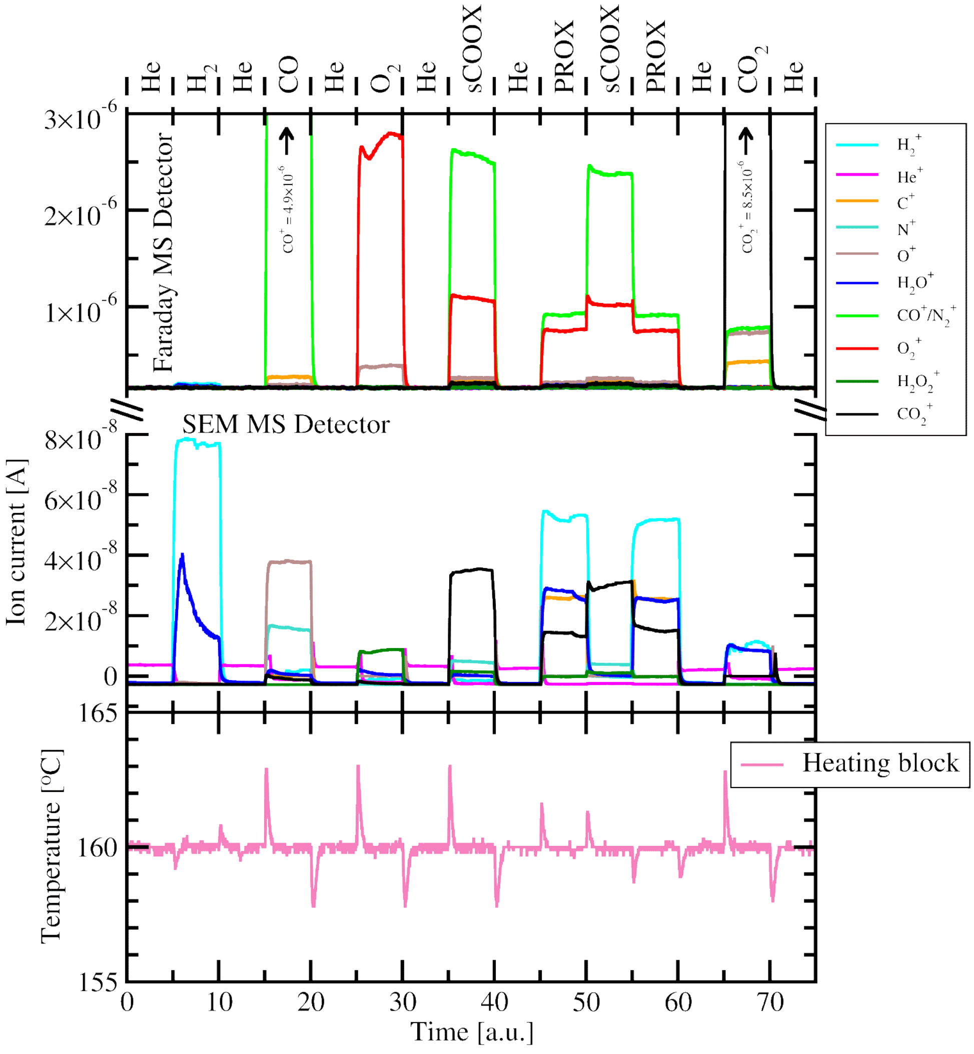

Fig. S40. Mass spectrometry (MS) analysis of composition of the gas stream leaving the glass U-tube reactor with the sintered glass disk alone. The actual temperature of the disk measured with glass shielded thermocouple (bottom) and MS signals corresponding to relevant cations are shown. Data are plotted against time with unit of approx. 36 min. The sCOOX phase corresponds to 7 ml/min of O_2 and 14 ml/min of CO, PROX- 10 ml/min of H_2, 5 ml/min of CO and 5 ml/min of O_2. Evident is non-linear response of the Faraday cup detector.

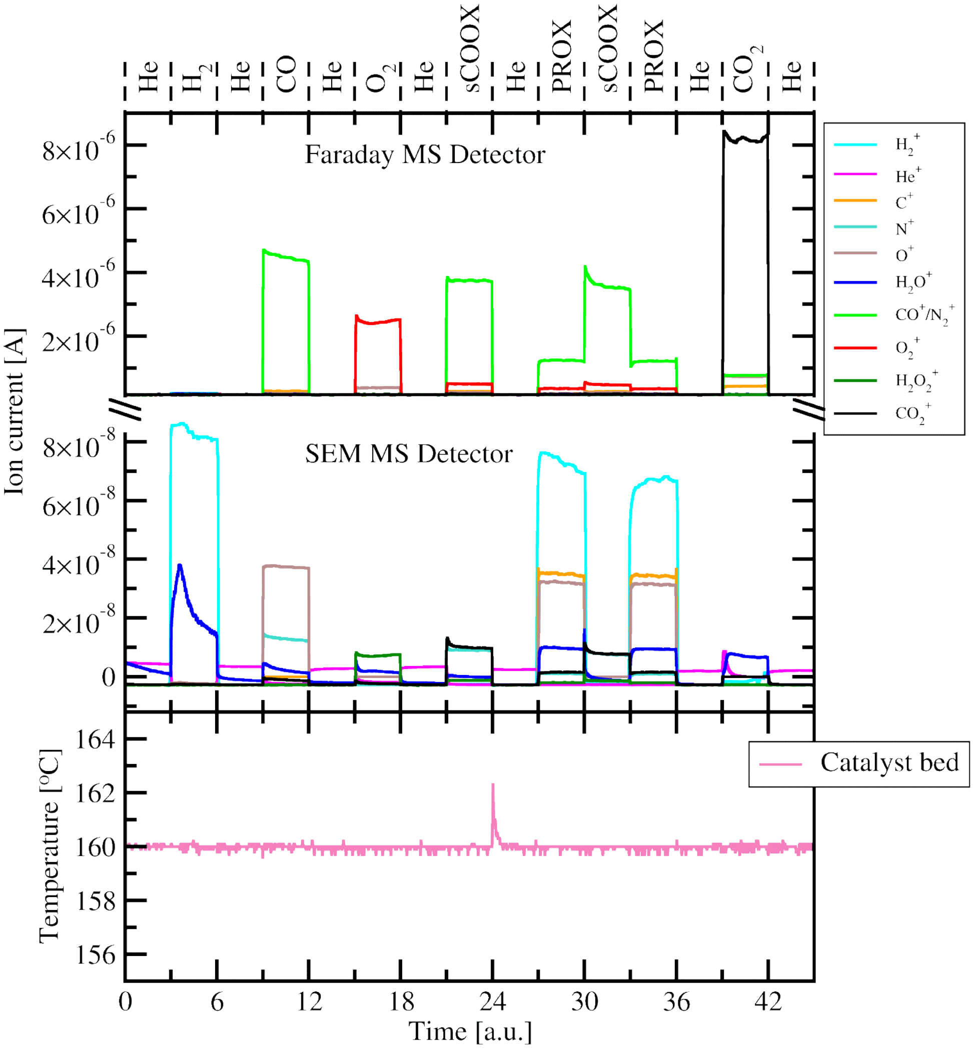

References

[1] J. Turkevich, P. C. Stevenson, J. Hillier, Disc. Farad. Soc., vol. 11, p. 55, (1951).
[2] K. C. Grabar, K. J. Allison, B. E. Barker, R. M. Bright, K. R. Brown, R. G. Freeman, A. P. Fox, C. D. Keating, M. D. Musick, M. J. Natan, Langmuir, vol. 12, no. 10, p. 2353, 1996.
[3] N. Kristian, X. Wang, Electrochem. Commun. 10 (2008) p.12, vol. 10, p. 12, 2008.
[4] E. Frota Jr., A. Purgatto, J. J. Linares, Chem. Eng. Trans., vol. 41, p. 253, 2014.
[5] Y. L. Lam, M. Boudart, J. Catal., vol. 50, p. 530, 1977.
[6] B. P. Block, J. C Bailar, J. Am. Chem. Soc., vol. 73, p. 4722, 1951.
[7] W. J. Louw, W. Robb, Inorg. Chim. Acta, vol. 3, no. 1, p. 29, 1969.
[8] K. Kitada. US Patent 6087516.
[9] Z. Kaszkur, M. Zielinski, W. Juszczyk, J. Appl. Cryst., vol. 50, p. 585, 2017.
[10] M. E. Milberg, J.Appl.Phys., vol.29, p.64, 1958.
[11] L.D. Marks, Philos.Mag.A, vol.49, p.81, 1984.
[12] A. P. Sutton, J. Chen, Phil. Mag. Lett., vol. 61, p. 139, 1990.
[13] B. Mierzwa, Z. Kaszkur, “Combined XRD-EXAFS Software Tools for Metal Nanoclusters,” in Applied Crystallography: Proceddings of the XIX Conference, Singapore, World Scientific Publishing Co. Pte. Ltd., 2003, p. 162. The program CLUSTER is available from Kaszkur, Z., and Mierzwa, B. (2021), http://kaszkur.net.pl/program-cluster/.

[14] V. Rosato, M. Guillopé, and B. Legrand, Philos. Mag. A59, p.321, 1989.
[15] F. Cleri and V. Rosato, Phys. Rev. B48, p.22, 1993.
[16] F. Baletto, C. Mottet, R. Ferrando, Surface Science, 446, p.31, 2000.
[17] M.F. Camellone, S. Fabris, J. Am. Chem. Soc. , vol.131, 30, p.10473, 2009.
[18] G.Antczak, G.Ehrlich, Surface Diffusion: Metals, Metal atoms, and Clusters, Cambridge University Press, ISBN 978-0-521-89983-3, 2010.
[19] J. Chang and E. Johnson, Philos.Mag., vol.85(30), p.3617, 2005.
[20] C.L. Cleveland, U. Landman, T.G. Schaaff, M.N. Shafigullin, P.W. Stephens, and R.L. Whetten, Phys.Rev.Lett., vol.79(10), p.1873,.1997.
[21] L.J. Lewis, P. Jensen, and J-L. Barrat, Phys. Rev. B, vol.**56**, 2248, 1997.
[22] Castro, T.; Reifenberger, R.; Choi, E.; Andres, R. P. Phys. Rev. B, vol.42, p.8548, 1990.
[23] Z. Kaszkur, W. Juszczyk, D.Łomot, Phys. Chem. Chem. Phys., vol. 17, p. 28250, 2015.
[24] D.G. Araiza, A. Gomez-Cortes, G. Diaz, Catal. Sci. Technol., vol.7, p.5224, 2017.
[25] C. Binet, A. Badri, M. Boutonnet-Kizling, J-C. Lavalley, J. Chem. Soc., Faraday Trans., vol.90, p.1023, 1994.
[26] C. Schilling, M. Ziemba, C. Hess, V. Ganduglia-Pirovano, Catal.Sci.Technol., vol.7, p.264, 2020.
[27] A.S. Barnard, N.P. Young, A.I. Kirkland, M.A. van Huis & H. Xu. ACS Nano, vol.3, p.1431, (2009).
[28] A.I. Kirkland, D.A. Jefferson, D. Tang, P.P. Edwards & J.E. Enderby, Proc. Royal Soc. London. Ser. A: Math. Phys. Sci., vol. 434, p.279, 1991.
[29] P.-A. Buffat, M. Flüeli, R. Spycher, P. Stadelmann & J.-P. Borel, Faraday Discuss., vol. 92, p.173, 1991.
[30] H. Hofmeister, Encyclopedia of Nanoscience and Nanotechnology, vol. 10, American Scientific Publishers, Valencia, CA, USA, 2003.
[31] K. Koga, T. Ikeshoji & K.-i. Sugawara, PhysRev. Lett., vol. 92, p.115507, 2004.
[32] L. D. Marks, Reports on Prog. Phys., vol.57, p.603, 1994.
[33] L. Marks & D.J. Smith, J.Cryst. Growth, vol.54, p.425, 1981.
[34] J. M. Penisson & A. Renou, Zeitschrift für Physik D Atoms, Mol. Clust., vol.12, p.113, 1989.
